# TGFβ1–TNFα-regulated secretion of neutrophil chemokines is independent of epithelial–mesenchymal transition in breast tumor cells

**DOI:** 10.1091/mbc.E25-07-0340

**Published:** 2025-09-12

**Authors:** Shuvasree SenGupta, Erez Cohen, Joseph Serrenho, Kaleb Ott, Pierre A. Coulombe, Carole A. Parent

**Affiliations:** ^a^Life Sciences Institute, University of Michigan, Ann Arbor, MI 48109; ^b^Department of Pharmacology, University of Michigan Medical School, Ann Arbor, MI 48109; ^c^Department of Cell and Developmental Biology, University of Michigan Medical School, Ann Arbor, MI 48109; ^d^Undergraduate Research Opportunity Program, University of Michigan, Ann Arbor, MI 48109; ^e^Rogel Cancer Center, University of Michigan Medical School, Ann Arbor, MI 48109; ^f^Department of Dermatology, University of Michigan Medical School, Ann Arbor, MI 48109; University of Wisconsin, Madison

## Abstract

Neutrophils exert tumor-promoting roles in breast cancer and are particularly prominent in aggressive breast tumors. The proinflammatory signals TGF-β1 and TNF-α are upregulated in breast tumors and induce epithelial-to-mesenchymal transitions (EMT), a process linked to cancer cell aggressiveness. Here, we investigated the roles of TGF-β1 and TNF-α in the recruitment of neutrophils by breast cancer cells. Dual-treatment with TGF-β1 and TNF-α induces EMT signatures in premalignant M2 cells, which are part of the MCF10A breast cancer progression model. Conditioned media (CM) harvested from M2 cells treated with TGF-β1/TNF-α gives rise to amplified neutrophil chemotaxis compared with CM from vehicle-treated M2 cells. This response correlates with higher levels of the neutrophil chemokines CXCL1 and CXCL8, in a p38MAPK-dependent manner, and is attenuated by CXCL8-neutralizing antibodies. We combined gene editing, immunological, and biochemical assays to show that neutrophil recruitment and EMT are uncoupled in treated M2 cells. Finally, analysis of transcriptomic databases of cancer cell lines revealed a significant correlation between CXCL8 and TGF-β1/TNF-α–regulated or effector genes in breast cancer. These findings establish a novel role for the TGF-β1/TNF-α/p38 MAPK signaling axis in regulating neutrophil recruitment in breast cancer, independent of their profound impact on EMT.

## Abstract (executive summary)

- Neutrophils promote breast cancer progression. However, the mechanisms regulating neutrophil recruitment to breast tumors are not clear.
- The authors combined gene editing, immunological and biochemical assays and identified a role of TGF-β1/ TNF-α/ p38MAPK in regulating neutrophil recruitment mediated by the secretion of the chemokines CXCL1/8. This regulatory action of TGF-β1/ TNF-α is independent of their role in promoting epithelial-to-mesenchymal transition and cancer cell aggressiveness. These findings are further strengthened by computational analyses of gene expression datasets from human cancer cell lines.
- This study reveals key molecular and signaling players that regulate neutrophil recruitment to tumors and underscores potential therapeutic opportunities.

## INTRODUCTION

The early and prominent role of neutrophils in response to infections or injuries is well-established. In addition, neutrophils are emerging as a key component of the tumor-microenvironment in a broad array of malignancies with multifaceted cancer-regulating functionalities. For instance, tumor-associated neutrophils impact all stages of tumor progression, including tumor initiation, growth, and metastasis by secreting reactive oxygen/nitrogen species, proteases, growth factors, proangiogenic factors, and neutrophil extracellular traps, and have been linked with poor patient prognosis (SenGupta *et al.*, 2019: Shaul and Fridlender, 2019; Martins-Cardoso *et al.*, 2020; Long *et al.*, 2021; Quail *et al.*, 2022; Yu *et al.*, 2024; Camargo *et al.*, 2025). In breast cancer, neutrophils and neutrophil-derived products are more frequently detected in the tumor niche of the highly aggressive triple-negative breast cancer (TNBC) subtype compared with the less aggressive hormone receptor–positive (HR^+^) subtype (Park *et al.*, 2016; Soto-Perez-de-Celis *et al.*, 2017; Wang *et al.*, 2019) and have been shown to promote cancer cell metastasis (Park *et al.*, 2016; Wang *et al.*, 2019). When comparing the neutrophil-recruiting abilities of cancer cell lines from different breast cancer subtypes, we showed that tumor-conditioned media (TCM) isolated from TNBCs induce greater neutrophil chemotaxis relative to TCM from HR^+^ breast cancer cells. We also found that chemokines specific to the CXCR2 receptor and transforming growth factor-β1 (TGF-β1) secreted from TNBC cells work in tandem to recruit neutrophils (SenGupta *et al.*, 2019; 2021a; 2021b). These findings indicate a link between cancer cell aggressiveness and neutrophil-recruiting capabilities.

A well-established response linked to cancer cell aggressiveness is the epithelial-to-mesenchymal transition (EMT) (Suarez-Carmona *et al.*, 2017; Pastushenko and Blanpain, 2019; Brabletz *et al.*, 2021). EMT is a complex cellular process, where epithelial cells within a tissue lose polarity and cell–cell adhesion and gain invasive properties as they adopt a mesenchymal character. This transition is dynamic, with cells oscillating between interconvertible hybrid epithelial/mesenchymal states and expressing varying degrees of EMT signature markers. EMT promotes cell motility, which plays a crucial role in mediating aggressiveness and metastasis (Bakir *et al.*, 2020; Grasset *et al.*, 2022; Sample *et al.*, 2023; Celià-Terrassa and Kang, 2024). In addition, positive correlations between EMT signatures and the composition of tumor-infiltrated immune cells have been observed in various tumors (Wang *et al.*, 2021; Heryanto and Imoto, 2023). In breast cancer, the increased presence of myeloid cells has been reported in TNBC tissues featuring higher expression of EMT markers (Suarez-Carmona *et al.*, 2015). Here, we sought to explore the relationship between EMT signatures and the ability of cancer cells to recruit neutrophils in the context of breast cancer.

EMT signatures are induced when epithelial cells are exposed to inflammatory mediators commonly encountered in tumor niches, such as cytokines, chemokines, and growth factors (Suarez-Carmona *et al.*, 2017; Lambert and Weinberg, 2021). The EMT-inducing effects of such inflammatory mediators are regulated by the activation of signaling pathways that induce the expression of classical EMT transcription factors (TF), including snail, twist, and zeb (Graham *et al.*, 2008; Li *et al.*, 2012; Gonzalez and Medici, 2014; Lee *et al.*, 2020). These EMT-driving TFs then control the expression of several mesenchymal signature markers such as vimentin (Vim), N-cadherin (N-Cad), and fibronectin (Fn), and epithelial signature marker E-cadherin (E-Cad) (Xu *et al.*, 2009; Ribatti *et al.*, 2020). In addition, activation of signaling pathways, for instance SMAD and nuclear factor κ-light-chain enhancer of activated B cells (NF-κB), can directly regulate the expression of EMT signature markers independently of the classical EMT TFs (Lilienbaum and Paulin, 1993; Huber *et al.*, 2004; Yang *et al.*, 2015; Usman *et al.*, 2021).

TGF-β, which belongs to a multifunctional cytokine family, is a well-established EMT inducer (Xu *et al.*, 2009). TGF-β exists in three isoforms, TGF-β1, TGF-β2, and TGF-β3, with TGF-β1 being the most prevalent in many cancer types (Martin *et al.*, 2020). TGF-β1 signals through the TβRI/TβRII heterotetrameric receptor complex, resulting in the activation of canonical SMAD-dependent and multiple noncanonical, SMAD-independent signaling pathways that include c-Jun NH2-terminal kinase (JNK), p38 mitogen-activated protein kinase (MAPK), extracellular signal–regulated kinase (ERK), phosphatidylinositol 3-kinase (PI3K)-protein kinase B (AKT), and NF-κB (Liu *et al.*, 2021a). TGF-β1 promotes EMT through both SMAD-dependent and -independent pathways (Gottumukkala *et al.*, 2024). Furthermore, TGF-β1 is produced by both cancer cells and stromal cells present in the tumor niche (Batlle and Massagué, 2019), and the presence of TGF-β1 in breast tumors has been associated with increased lymph node metastasis (Walker *et al.*, 1994). We also found that aggressive TNBC cell lines secrete high levels of TGF-β1 (SenGupta *et al.*, 2021b). Whether the activation of TGF-β1 signaling pathways impacts the ability of cancer cells to recruit neutrophils to breast tumors remains, however, unknown.

Another EMT-inducing factor, tumor necrosis factor-α (TNF-α), is an important proinflammatory cytokine that is highly upregulated in breast cancer cells and secreted by stromal cells (Soria *et al.*, 2011; Cruceriu *et al.*, 2020). TNF-α belongs to the TNF/TNFR superfamily of cytokines and binds TNFR1 to activate JNK, MAPK, and NF-κB signaling pathways (Gough and Myles, 2020). TNF-α also coordinates with TGF-β to induce EMT in multiple cancer types, including breast cancer (Kawata *et al.*, 2012; Saito *et al.*, 2013; Liao *et al.*, 2019; Liu *et al.*, 2021b). The combined action of TNF-α and TGF-β1 can potentiate the activation of signaling pathways and induce robust EMT signatures and cancer cell invasiveness (Liao *et al.*, 2019). Yet, how the combined action of TGF-β1 and TNF-α controls the secretion of neutrophil guidance cues in breast tumors has not been studied.

In this study, we used cell lines of the MCF10A breast cancer progression model (Dawson *et al.*, 1996; Santner *et al.*, 2001) as well as human breast cancer cell lines (Neve *et al.*, 2006) to identify the mechanisms by which aggressive breast cancer cells recruit neutrophils. In particular, we assessed the role of TGF-β1/TNF-α treatments and EMT-associated changes on this response and validated our experimental findings by conducting a comprehensive analysis of transcriptomics datasets from breast cancer cell lines. We found that the neutrophil recruitment ability is not regulated by EMT signatures. Instead, these two processes appear to occur in parallel and can be triggered by distinct signaling pathways downstream of TGF-β1/TNF-α.

## RESULTS

### TGF-β1/TNF-α–treated M2 cells recruit neutrophils by inducing chemokine secretion

**FIGURE 1:**
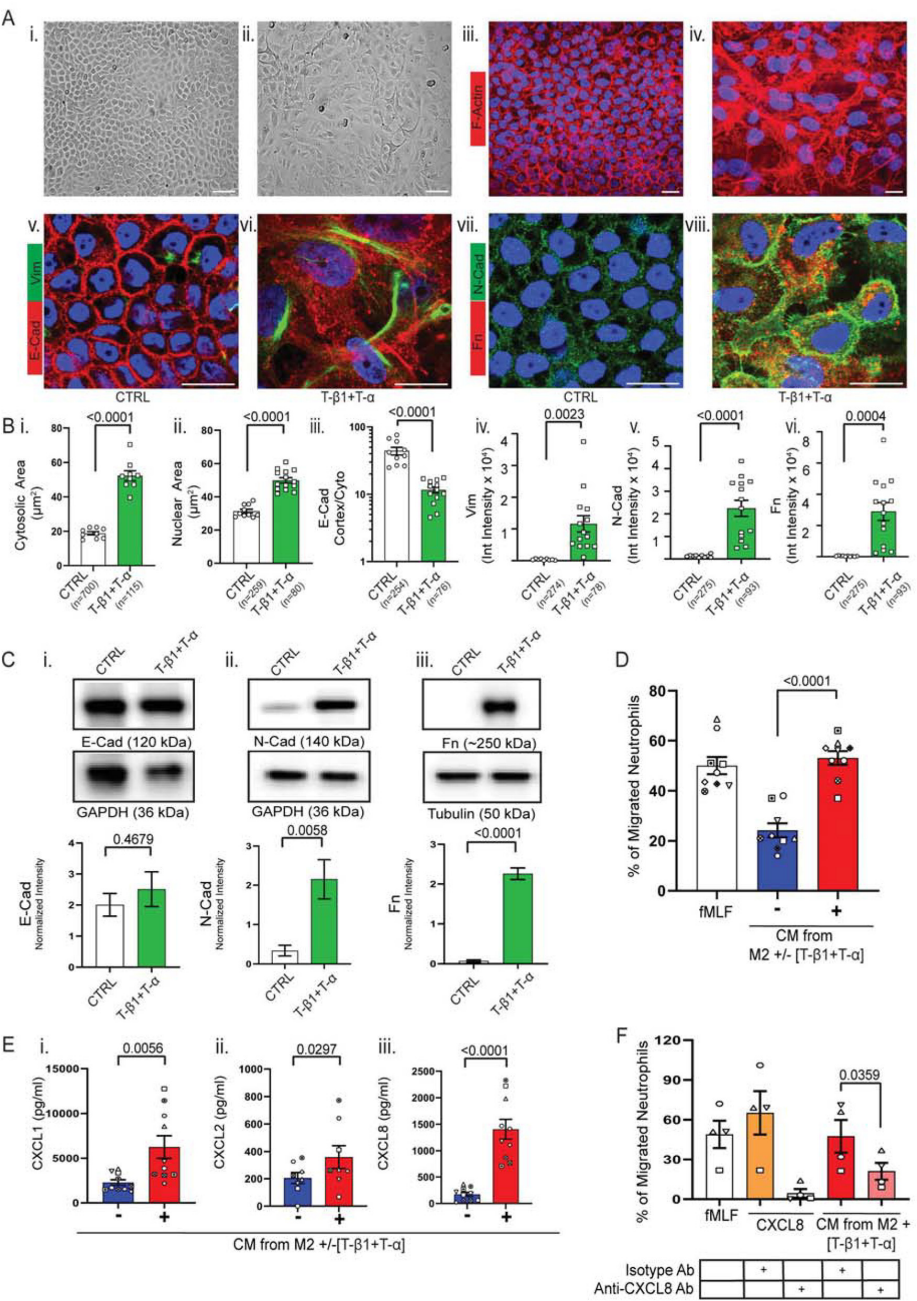
TGF-β1/TNF-α treatment amplifies the neutrophil-recruiting activity of M2 cells by inducing chemokine secretion. (A) Representative bright-field (i and ii) and IF images (iii–viii) (*n* = 3) of control (CTRL) M2 cells (i, iii, v, and vii) or M2 cells treated with a combination of 20 ng/ml T-β1 (TGF-β1) and 100 ng/ml T-α (TNF-α) [T-β1+ T-α] for 72 h (ii, iv, vi, and viii). Airyscan confocal microscopy images showing MIP (iii and iv) or a single *z* image (v–viii) of fixed M2 cells stained for F-actin with phalloidin-TRITC (red) (iii and iv), E-Cad (red)/Vim (green) (v and vi), N-Cad (green)/Fn (red) (vii and viii), and nuclei with DAPI (blue). Individual channel images are provided in Supplemental Figure S4, C–E. Scale bar 50 µm for bright-filed images and 20 µm (iii–viii) for IF images. (B) Graphs depicting cytosolic (i) and nuclear (ii) area or cortex/cytosolic intensity ratio of E-Cad (iii) or integrated intensity measures of Vim, N-Cad, Fn (iv, v, and vi) in control versus T-β1/T-α–treated M2 cells. Each dot represents an average of all cells in each image (≥3 images/condition/experiment). Total number of cells (*n*) analyzed is reported under each condition. (C) Top: Representative Western blots of the respective markers from CTRL or T-β1/T-α–treated cells. Bottom: Graphs showing band intensities of the markers normalized to the loading controls (mean values ± SEM from *n* = 3). (D) Graph depicting the percentage of neutrophils that migrated into the bottom chamber of Transwells containing equal volume of CM from CTRL or T-β1/T-α–treated M2 cells or positive control fMLF (mean values ± SEM from *n* = 9). Each dot represents the response of neutrophils from an independent donor. (E) Graphs showing the amount (pg/ml) of CXCL1 (i), CXCL2 (ii), and CXCL8 (iii) secreted by CTRL or T-β1/T-α–treated M2 cells (mean values ± SEM from *n* = 8–10). Each dot represents the value from one experiment. (F) Graphs showing the percentage of neutrophils that migrated in response to CXCL8 or CM derived from T-β1/T-α–treated M2 and preincubated with anti-CXCL8 antibody or isotype control or to control fMLF (mean ± SEM from *n* = 4). *P* values were determined using unpaired *t* test (B and C) or paired *t* test (D–F).

We first used the nonmalignant MCF10A cells, the M1 cells in the breast cancer progression model (Dawson *et al.*, 1996; Santner *et al.*, 2001) to investigate the effect of TGF-β1/TNF-α treatment on their neutrophil recruitment ability. However, these cells failed to survive when subjected to serum-free conditions for the collection of the CM, a step necessary to minimize neutrophil activation by the factors present in the serum (SenGupta *et al.*, 2021b). We therefore used M2 cells, an H-Ras transformed, premalignant derivative of the M1 cells that undergo minimal cell death under serum-free condition (Dawson *et al.*, 1996; Santner *et al.*, 2001). Furthermore, M2 cells can be directly compared with their malignant counterpart M4 cells, because both originate from the same patient and therefore share a common genetic background—unlike TNBC cell lines, which come from different breast cancer patients. We first characterized the effect of TGF-β1/TNF-α dual-treatment on the morphology of M2 cells. Although control, nontreated cells retain a tightly packed, cuboidal and epithelial-like morphology (Figure 1Ai), cells treated for 72 h become loosely organized and acquire an elongated, spindle-like shape with front–back polarity (Figure 1Aii), consistent with a mesenchymal phenotype (Yang *et al.*, 2020; Celià-Terrassa and Kang, 2024). This effect on morphology is detected as early as 24 h after TGF-β1/TNF-α addition and is independent of cell density (Supplemental Figure S4A). The treated cells also display dramatic changes in actin filament organization and an increase in cytosolic and nuclear areas compared with control cells (Figure 1, Aiii–iv and Bi–ii). Immunostaining revealed that expression of the mesenchymal markers Vim, N-Cad, and Fn are upregulated with TGF-β1/TNF-α dual-treatment, consistent with EMT-associated changes (Figure 1, Av–viii and Biv–vi). TGF-β1/TNF-α treatment also induced a profound redistribution of E-Cad, from being strictly localized to cell–cell junctions in control cells (Figure 1Av) to a more diffused cytosolic distribution in treated cells (Figure 1Avi), along with a decrease in the cortex to cytosolic ratio of E-Cad intensity (Figure 1Biii). Although treated cells retain a similar level of total E-Cad compared with control cells (Figure 1Ci), increases in the total protein levels of N-Cad and Fn were confirmed by Western blotting (Figure 1, Cii–iii).

We next characterized the neutrophil recruitment ability of CM harvested from control and TGF-β1/TNF-α–treated M2 cells. We measured similar protein content in CM from control and treated cells (Supplemental Figure S4B). Using a neutrophil Transwell migration assay, we found that the CM from treated cells give rise to a robust neutrophil chemotactic response, comparable with the activity of the formylated bacterial tripeptide fMLF (Figure 1D)—a potent neutrophil chemoattractant (Schiffmann *et al.*, 1975a; 1975b). To identify the neutrophil-recruiting factors secreted from treated M2 cells, we first used ELISA and screened for CXCR2 ligands and TGF-β1 both abundantly secreted by aggressive M4 cells and involved in the regulation of neutrophil chemotaxis induced by M4-derived CM (SenGupta *et al.*, 2021b). Although there was undetectable or minimal TGF-β1 present in the CM regardless of treatment (Supplemental Table S1), we detected >8-fold increase in CXCL8, a 3-fold increase in CXCL1 and a slight increase in CXCL2 levels in the CM from treated cells relative to CM from control cells (Figure 1, Ei–iii). Because CXCL8 is a highly potent neutrophil chemokine, we tested whether blocking CXCL8 with a specific neutralizing antibody (Ab) in the Transwell system would reverse the effect of CM from treated cells on neutrophil migration. Incubation of CM from treated cells with anti-CXCL8 Ab at a dose that was sufficient to inhibit recombinant CXCL8-induced neutrophil migration significantly reduced the ability of CM from TGF-β1/TNF-α–treated cells to induce neutrophil chemotaxis, compared with the activity of the same CM incubated with a corresponding isotype Ab (Figure 1F). Taken together, these findings establish a role for TGF-β1/TNF-α treatment in inducing changes associated with EMT-like states and in regulating neutrophil chemotaxis by stimulating the release of neutrophil-recruiting chemokines, CXCL8 in particular, from M2 cells.

### TGF-β1/TNF-α–treated M2 cells recruit neutrophils in a snail- and twist-independent manner

Several TFs, including snail, twist, slug, and zeb1/2 serve as EMT master regulators (Zheng and Kang, 2014; Saitoh, 2023). Here, we focused on snail and twist as they both promote breast cancer metastasis (Soini *et al.*, 2011; Tran *et al.*, 2014; Cao *et al.*, 2018) and have been implicated in regulating cytokine/chemokine expressions in cancer cells (Sullivan *et al.*, 2009; Suarez-Carmona *et al.*, 2015). Furthermore, snail has been reported to stimulate myeloid cell recruitment to murine tumor models of lung and ovarian cancer (Faget *et al.*, 2017; Taki *et al.*, 2018). We investigated the role of snail and twist on neutrophil recruitment by exogenously expressing an active mutant form of snail (eGFP-snail6SA) tagged with green fluorescent protein (GFP) (Zhou *et al.*, 2004) or twist in M2 cells and, in parallel studies, by depleting these TFs from M2 and TNBC cell lines. Although M2 and M2-GFP control cells had low endogenous expression of snail and twist (Supplemental Figure S1, A–C; see high exposure images), we measured a robust expression of eGFP-snail and twist in M2-eGFP-snail and M2-twist–expressing cells (Supplemental Figure S1, A–C). We also detected a strong nuclear localization of eGFP-snail in M2-eGFP-snail cells and of twist in M2-twist cells (Supplemental Figure S1D). Conversely, using shRNA, we obtained an ∼70% knockdown (KD) of snail in the invasive breast cancer BT549 cells (Neve *et al.*, 2006; SenGupta *et al.*, 2021b) (Supplemental Figure S1E) and a depletion of twist in M2, M4, and BT549 twist knockout (KO) cell lines (Supplemental Figure S1, Fi–iii).

**FIGURE 2:**
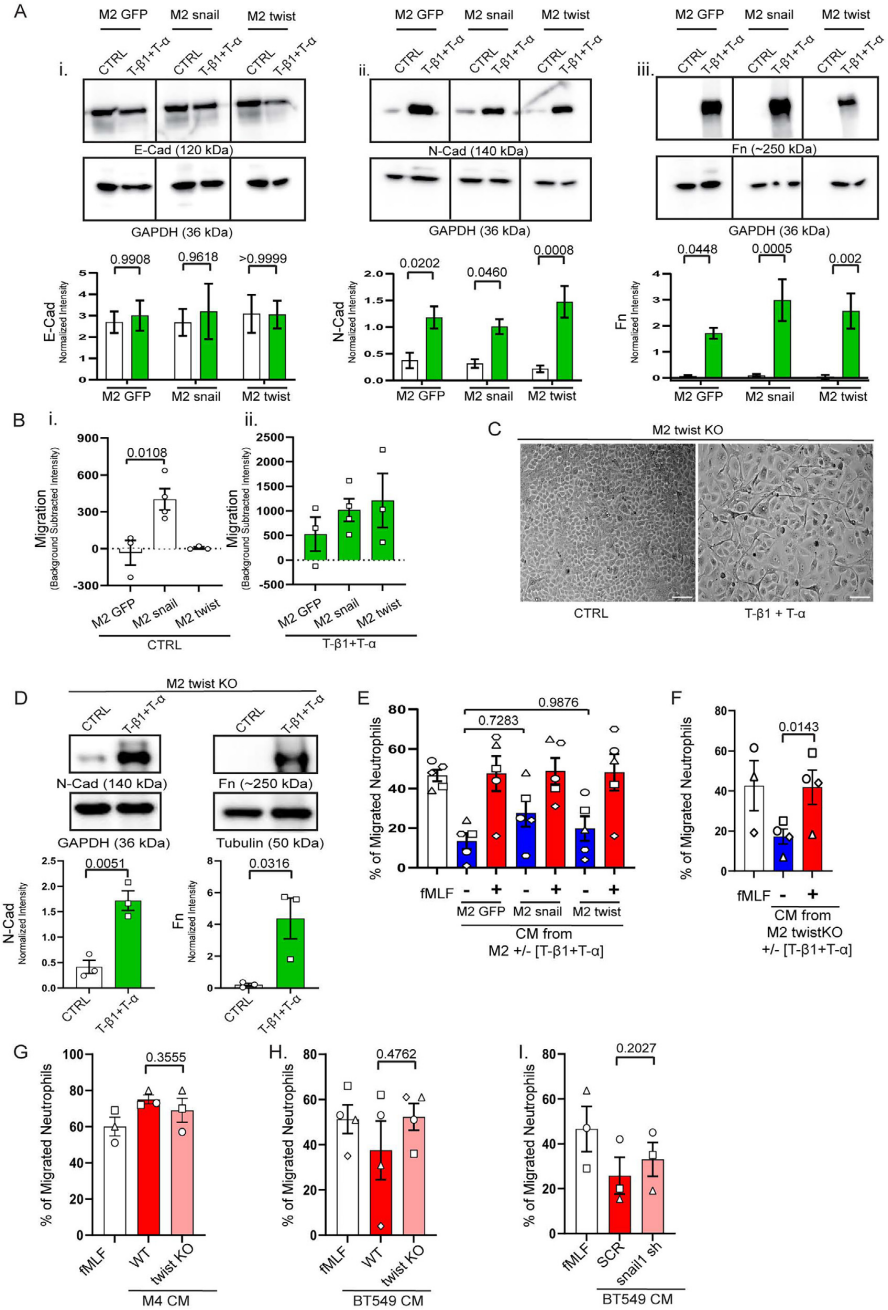
Neutrophil recruitment by TGF-β1/TNF-α–treated M2 cells is independent of snail and twist. (A) Top: Representative Western blots showing the expression of (i) E-Cad, (ii) N-Cad, and (iii) Fn in CTRL versus T-β1/T-α–treated cells. Bottom: Graphs showing band intensities of the markers normalized to the respective loading controls (mean values ± SEM from *n* = 3). (B) Graphs depicting normalized fluorescence intensity measurements of migrated CTRL or T-β1/T-α–treated cells (mean values ± SEM from *n* = 3–4). (C) Representative (*n* = 3) bright-field images showing morphological changes in M2 twist KO cells with T-β1/T-α treatment. Bar, 50 µm. (D) Top: Representative Western blots of N-Cad and Fn expression in M2 twist KO cells. Bottom: Graphs showing band intensities of the markers normalized to the respective loading controls (mean values ± SEM from *n* = 3). (E and F) Graphs depicting the percentage of neutrophils that migrated into the bottom chamber of Transwells containing equal volume of CM from CTRL or T-β1/T-α–treated cells or positive control fMLF (mean values ± SEM from *n* = 3–5). Each dot represents response of neutrophils from independent donors. (G–I) Graphs depicting the percentage of neutrophils that migrated into the bottom chamber of Transwells containing equal volume of CM from WT or twist KO cell lines for M4 (G) and BT549 (H), and from the SCR or snail sh cell line for BT549 (I) or positive control fMLF (mean values ± SEM from *n* = 3–4). Each dot represents response of neutrophils from independent donors. *P* values were determined using two-way ANOVA with Sidak (A) or one-way ANOVA with Dunnett's (B) or Turkey (E) multiple comparisons test or unpaired (D) or paired (F–I) *t* test.

We found that expression of E-Cad, N-Cad, and Fn is similar in M2-GFP, M2-eGFP-snail, and M2-twist cells in the absence of TGF-β1/TNF-α (Figure 2, Ai–iii), suggesting that expression of either snail or twist does not suffice to induce EMT in M2 cells. Dual-treatment with TGF-β1/TNF-α upregulated the expression of Fn and N-Cad without affecting E-Cad expression (Figure 2, Ai–iii). In agreement with the established role of snail in promoting cell motility (Zhou *et al.*, 2004; Lyons *et al.*, 2008; Smith *et al.*, 2014; Henderson *et al.*, 2015), we also observed a greater proportion of untreated M2-eGFP-snail cells migrating toward serum compared with the M2-GFP or M2-twist cells (Figure 2Bi). All three cell lines similarly migrated toward serum with dual-treatment (Figure 2Bii). Surprisingly, we found that TGF-β1/TNF-α treatment of M2-twist KO cells induced a mesenchymal cell-like morphology (Figure 2C) and significantly increased the expression of both N-Cad and Fn (Figure 2D). Together, these findings suggest that snail and twist are dispensable for the expression of EMT markers in M2 cells.

We next evaluated the neutrophil-recruiting ability of CM harvested from M2 cells with altered levels of snail and twist expressions. We found that CM harvested from M2-eGFP-snail cells recruit a marginally greater percentage of neutrophils isolated from four out of five donors, compared with M2-GFP cell-derived CM. There was no difference in neutrophil migration induced by the CM harvested from M2-twist cells relative to CM from M2-GFP cells (Figure 2E). CM harvested from all three cells lines after TGF-β1/TNF-α treatment induced similar levels of neutrophil migration (Figure 2E), as did CM harvested from M2-twist KO cells treated with TGF-β1/TNF-α (Figure 2F). Next, we compared the neutrophil-recruiting ability of CM derived from two aggressive breast cancer cell lines, M4 and BT549, and their twist KO counterparts. We found that CM from both twist KO cell lines retain robust neutrophil-recruiting activity (Figure 2, G and H). We did, however, find that twist is required for the invasion ability of BT549 cells in a Transwell invasion assay setup (Supplemental Figure S1G), suggesting that the neutrophil-recruiting ability of these cells is not linked to their ability to invade. Similarly, there was no difference in the neutrophil-recruiting ability of CM harvested from snail KD BT549 cells compared with CM derived from SCR control cells (Figure 2I). Together, these results provide evidence that snail or twist have no direct role of in regulating the ability of breast cancer cells to recruit neutrophils.

### TGF-β1/TNF-α treatment does not impact the neutrophil-recruiting activity of TNBC cell lines

We previously reported that CM harvested from the TNBC cell lines M4, MDA-MB-231, and BT549 induces strong neutrophil migration (SenGupta *et al.*, 2021b). Here, we examined whether TGF-β1/TNF-α treatment further stimulates the neutrophil-recruiting abilities of these cells. First, we characterized the effect of the dual-treatment on EMT-associated markers. Although the amount of E-Cad remained unchanged (Supplemental Figure S2Ai), we measured an increased in both N-Cad and Fn expressions in dual-treated M4 cells (Supplemental Figure S2, Ai–iii). In MDA-MB-231 cells, TGF-β1/TNF-α treatment marginally decreased expression of Fn (Supplemental Figure S2B). In contrast, we detected an increase in Fn expression in BT549 cells with dual-treatment (Supplemental Figure S2C). Finally, we found that the neutrophil-recruiting activity of CM harvested from TNBC cells was unaffected by the TGF-β1/TNF-α dual-treatment (Supplemental Figure S2, D–F). Together, these results show that TGF-β1/TNF-α treatment elicits variable changes in the expression of EMT-associated markers in various TNBC cell lines and does not impact the neutrophil-recruiting ability of TNBC-derived CM.

### TNF-α amplifies the neutrophil-recruiting activity of M2 cells

**FIGURE 3:**
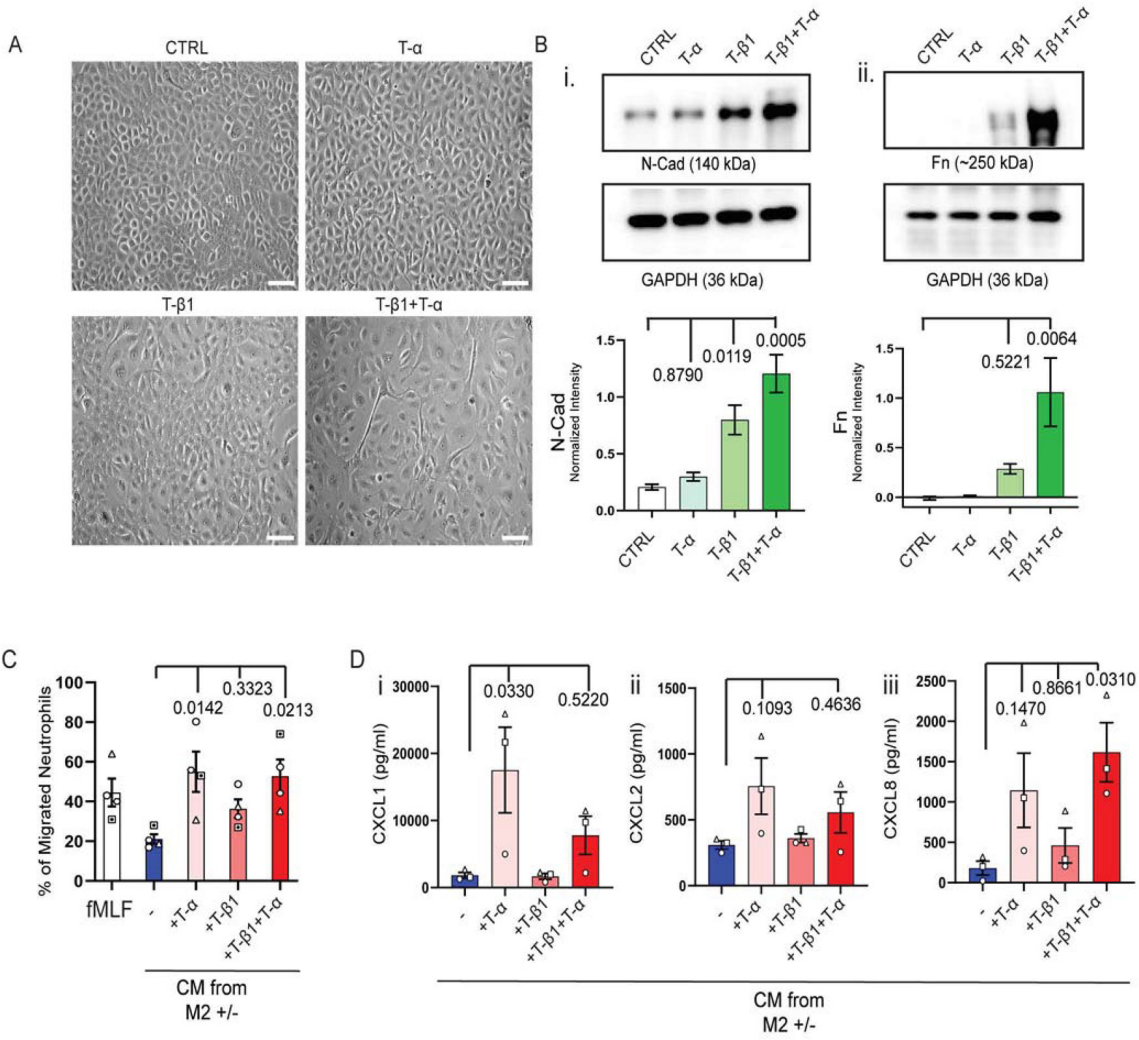
TNF-α treatment amplifies the neutrophil-recruiting activity of M2 cells without inducing EMT-associated changes. (A) Representative (*n* = 3) bright-field images showing the morphology of CRTL, T-β1, T-α, or T-β1/T-α–treated M2 cells; scale bar, 50 µm. (B) Top: Representative Western blots showing the expression of (i) N-Cad and (ii) Fn in CTRL and T-β1, T-α, or T-β1/T-α–treated cells. Bottom: Graphs showing band intensities of the markers normalized to the respective loading controls (mean ± SEM from *n* = 3). (C) Graph depicting the percentage of neutrophils that migrated into the bottom chamber of Transwells containing equal volume of CM from CTRL, T-β1, T-α, or T-β1/T-α–treated M2 cells (mean ± SEM from *n* = 4). Each dot represents response of neutrophils from independent donors. (D) Graphs showing the amount of CXCL1 (i), CXCL2 (ii), and CXCL8 (iii) secreted from CTRL, T-β1, T-α, or T-β1/T-α–treated M2 cells (mean ± SEM from *n* = 3). *P* values were determined using one-way ANOVA with Dunnett's multiple comparisons test (B–D).

TGF-β1 and TNF-α have regulatory roles during tumor progression (Montfort *et al.*, 2019; Dash *et al.*, 2021; Liu *et al.*, 2021b). Both acts synergistically to promote EMT-associated changes in noninvasive breast cancer cells (Liao *et al.*, 2019). To assess whether TGF-β1 and TNF-α synergize toward regulating neutrophil recruitment, we compared M2 cell responses with dual versus single TGF-β1 or TNF-α treatments. We found that only dual-treated cells undergo dramatic morphological changes, losing epithelial sheet-like alignment and acquiring spindle-shaped mesenchymal features (Figure 3A). When analyzing EMT-associated markers, we found that while TNF-α failed to increase the expression of N-Cad and Fn, TGF-β1 upregulated the expression of both markers—a response that was further amplified with dual-treatment (Figure 3, Bi–ii). Surprisingly, CM from TNF-α–treated M2 cells induced neutrophil migration as robustly as CM from dual-treated cells, while only a moderate increase in neutrophil migration occurred when using CM from TGF-β1–treated M2 cells (Figure 3C). Next, we evaluated how the various treatments modulate the profiles of neutrophil chemokines secreted from M2 cells. We measured a significant increase in the amount of CXCL1 in CM harvested from TNF-α–treated M2 cells compared with control cells and no significant increase when cells were dual-treated (Figure 3Di). We also noted a mild increase in the amount of CXCL2 in the CM collected from TNF-α–treated M2 cells (Figure 3Dii). In contrast, we detected a gradual increase in the amount of CXCL8 in the CM, with a moderate increase following TNF-α treatment and a significant increase following dual-treatment compared with control cells (Figure 3Diii). However, TGF-β1 alone failed to induce the secretion of CXCL1,CXCL2 or CXCL8 from M2 cells (Figure 3, Di–iii). Together, these results provide evidence that TGF-β1 and TNF-α do not synergistically amplify the neutrophil-recruiting activity of M2 cells and suggest that the changes in neutrophil-recruiting activity of M2 cells and alterations in EMT-associated markers are not interdependent.

### p38MAPK regulates the secretion of neutrophil-recruiting chemokines in TGF-β1/TNF-α–treated M2 cells

**FIGURE 4:**
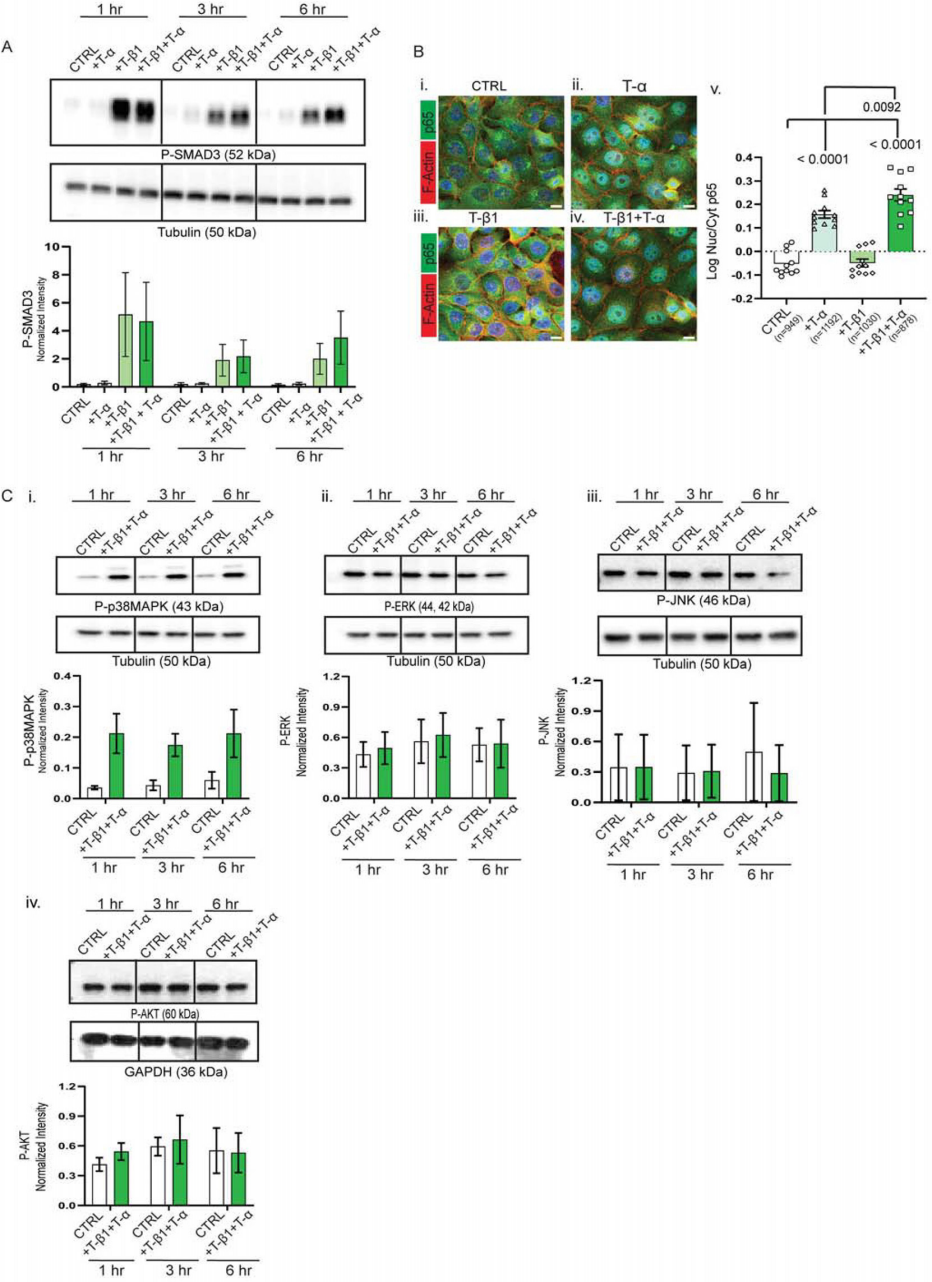
Signaling pathways activated in M2 cells treated with TGF-β1/TNF-α. (A) Top: Representative Western blots showing P-Smad3–specific bands over the course of 1, 3, and 6 h of treatments. Bottom: Graphs depicting band intensities of P-Smad3 normalized to the loading control (mean ± SEM from *n* = 3). Western blot of total Smad3 is provided in Supplemental Figure S3. (B) Representative IF images showing MIPs of fixed CRTL M2 cells or M2 cells treated for 30 min with T-β1, T-α, or T-β1/T-α and stained for p65 (green), F-actin with phalloidin-TRITC (red), and nucleus (blue) (i–iv); scale bar, 10 µm. Graph (v) depicting the nuclear/cytosolic intensity ration for p65. Each dot represents average of all cells in each image (three images/condition/experiment). Total number of cells (*n*) analyzed is depicted under each condition. Individual channel images are provided in Supplemental Figure S4F. (C) Representative Western blots showing P-p38MAPK (i), P-ERK (ii), P-JNK (iii), or P-AKT (iv) specific bands over the course of 1, 3, and 6 h of treatments. Graphs show band intensities of the respective phospho-proteins normalized to the loading control (mean values ± SEM from *n* = 3). Western blots of total p38MAKP, ERK, JNK, and AKT are provided in Supplemental Figure S3. *P* values were obtained using one-way ANOVA with Dunnett's multiple comparisons test (Bv).

To further understand the mechanisms underlying the amplified neutrophil-recruiting activity of TGF-β1/TNF-α–treated M2 cells, we examined key signaling pathways activated downstream of TGF-β1, TNF-α or TGF-β1/TNF-α dual-treatments. We first assessed the activation status of the TGF-β1–specific effector SMAD3 (Ikushima and Miyazono, 2010; Huang *et al.*, 2022; Miyazawa *et al.*, 2024) and the TNF-α–specific effector NF-κβ (Xia *et al.*, 2014; Gough and Myles, 2020; Yu *et al.*, 2020) in M2 cells. As expected, we confirmed that TGF-β1 gives rise to SMAD3 phosphorylation (P-SMAD3) in M2 cells, while TNF-α does not, and that the amount of P-SMAD3 remains similar in dual-treated versus TGF-β1–treated M2 cells over periods of 1, 3, and 6 h after treatment (Figure 4A). We also confirmed that total SMAD3 levels are similar with both treatments (Supplemental Figure S3A). To assess the activity status of NF-κβ, we monitored the nuclear translocation of p65, a component of the p50/p65 heterodimer (Pires *et al.*, 2017). We found that TNF-α treatment induces a strong nuclear translocation of p65 in M2 cells—quantified as a high nuclear/cytosolic p65 ratio (Figure 4, Bii and v). In contrast, p65 remains cytosolic in TGF-β1–treated M2 cells and, as expected, in control cells (Figure 4, Bi, iii, and v). Interestingly, we detected a significant increase in the nuclear/cytosolic p65 ratio when M2 cells are dual-treated with TGF-β1/TNF-α compared with a single TNF-α treatment, suggesting cross-talk between the two pathways (Figure 4, Biv and v). Next, we evaluated the activation of MAPK and AKT signaling pathways in dual-treated M2 cells, as these downstream signaling events are known to promote EMT-associated changes in cancer cell lines (Gonzalez and Medici, 2014; Xu *et al.*, 2015; Liao *et al.*, 2019). Over periods of 1, 3, and 6 h after treatment, we measured a strong and sustained p38MAPK phosphorylation (P-p38MAPK) signal in response to TGF-β1/TNF-α dual-treatment in M2 cells (Figure 4Ci), while total p38MAPK levels were similar irrespective of treatment (Supplemental Figure S3B). In contrast, no change in the phosphorylation amount or total amount of either ERK, JNK, or AKT with TGF-β1/TNF-α treatment was measured (Figure 4, Cii–iv; Supplemental Figure S3, C–E).

**FIGURE 5:**
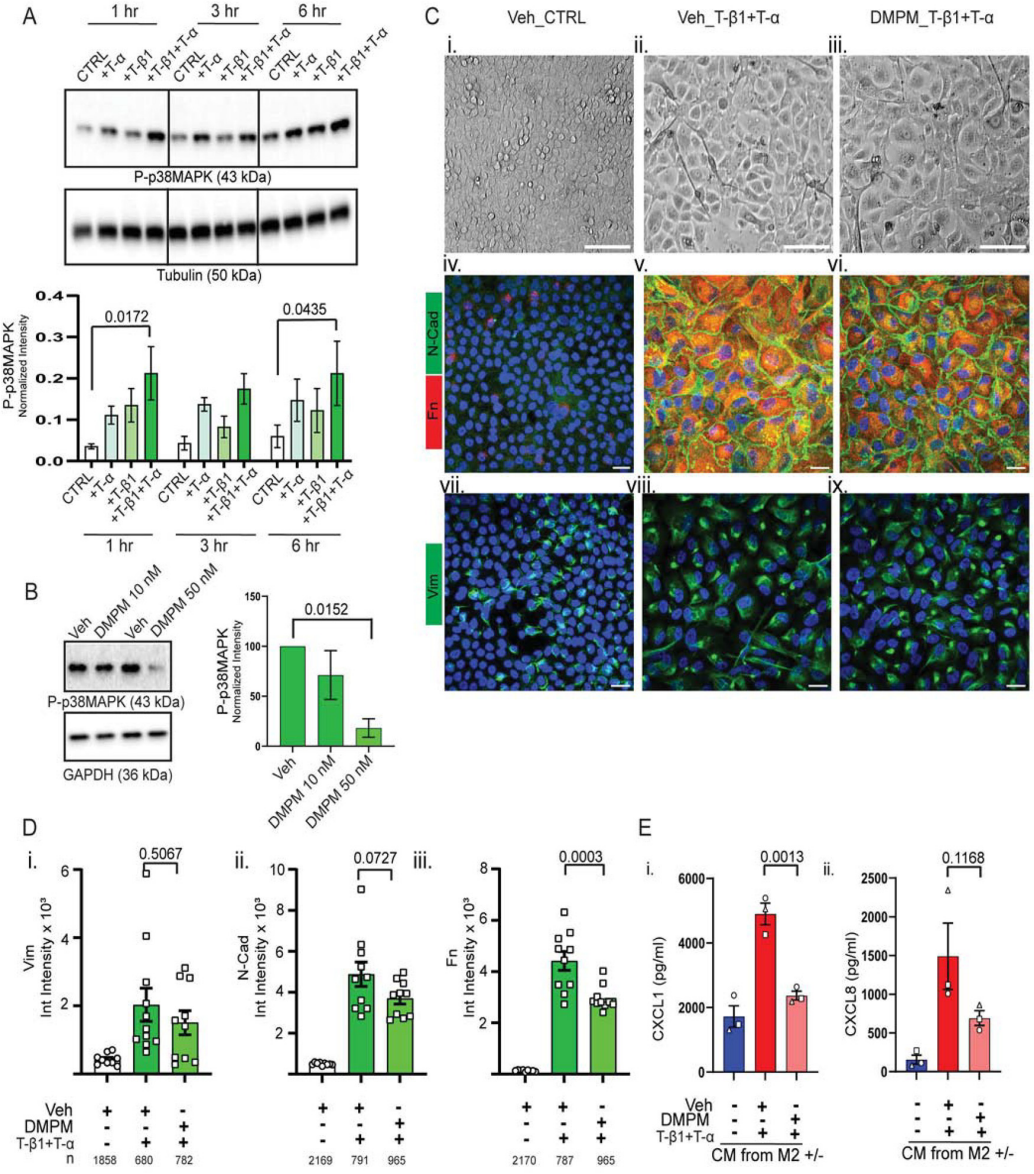
P-p38MAPK regulates the secretion of neutrophil-recruiting chemokines in TGF-β1/TNF-α–treated M2 cells. (A) Top: Representative Western blot showing P-p38MAPK–specific bands in CTRL M2 cells or M2 cells treated with T-β1, T-α or T-β1+T-α over the course of 1, 3, and 6 h. Bottom: Graph depicting band intensities of P-p38MAPK normalized to the loading control (mean ± SEM from *n* = 3). Representative Western blot of total p38MAKP is provided in Supplemental Figure S3. (B) Left: Representative Western blot showing P-p38MAPK–specific bands in M2 cells pretreated with 10 or 50 nM of DMPM or vehicle control and stimulated with T-β1+T-α for 72 h. Right: Graph depicting band intensities of P-p38MAPK normalized to loading control and represented as the percentage of vehicle control (mean ± SEM from *n* = 3). Representative Western blot of total p38MAKP is provided in Supplemental Figure S3. (C) Representative bright-filed (i–iii) or IF (iv–ix) images (*n* = 3) showing MIPs of M2 cells pretreated with DMPM or vehicle control and stimulated with T-β1+T-α for 72 h. Cells were stained for N-Cad (green)/Fn (red) (iv–vi) or Vim (green) (vii–ix) and nucleus (blue); scale bar, 100 µm (bright-field) or 20 µm (IF). Individual channel images are provided in Supplemental Figure S4, G and H. (D) Graphs depicting integrated intensity measures of Vim (i), N-Cad (ii), and Fn (iii). Each dot represents average of all cells in each image (≥3 images/condition/experiment). Total number of cells (*n*) analyzed is depicted under each condition. (E) Graphs showing amount of CXCL1 (i) and CXCL8 (ii) secreted by T-β1+T-α–treated M2 cells in the presence of DMPM or vehicle control (mean values ± SEM from *n* = 3). *P* values were determined using two-way (A) and one-way (B, D, and E) ANOVA with Dunnett's multiple comparisons test.

Next, we dissected the effect of TGF-β1 and TNF-α on p38MAPK activation. Although individual TGF-β1 or TNF-α treatments induced a moderate but nonsignificant increase in P-p38MAPK without affecting total protein levels, dual-treated M2 cells exhibited a significant increase in P-p38MAPK relative to control cells at 1 and 6 h poststimulation (Figure 5A; Figure S3F). As p38MAPK activity is known to regulate the expression of EMT-associated markers in breast epithelial and cancer cell lines (Werden *et al.*, 2016) and also controls the expression of several cytokines and chemokines (Hashimoto *et al.*, 1999; Parhar *et al.*, 2003; Petrova *et al.*, 2020; Canovas and Nebreda, 2021), we next assessed the effect of p38MAPK inhibition on TGF-β1/TNF-α–mediated EMT markers and chemokine production in M2 cells. We analyzed the morphology of cotreated M2 cells in the presence of vehicle control or doramapimod (DMPM), a potent p38MAPK inhibitor (Kuma *et al.*, 2005; Zhao *et al.*, 2021), at a dose that effectively inhibits P-p38MAPK (Figure 5B) but maintains total p38MAPK levels (Supplemental Figure S3G). Interestingly, we found that the mesenchymal phenotypes induced by dual-treatment is retained in the presence of the inhibitor (Figure 5C). Although we measured a decrease in the expression of Fn with the inhibitor treatment (Figure 5, C and Diii), we observed no changes in the upregulation of N-Cad and Vim expressions in cotreated M2 cells in the presence of the inhibitor (Figure 5, C and Di–ii). In contrast, we measured a substantial decrease in the amount of both CXCL1 and CXCL8 in the CM harvested from dual-treated M2 cells in the presence of the inhibitor compared with the vehicle control (Figure 5, Ei and ii). Together, these findings indicate that p38MAPK activity occurring downstream of TGF-β1/TNF-α dual-treatment regulates the expression of neutrophil-recruiting chemokines independently of EMT-associated changes. Furthermore, these findings add to the observation that EMT can be uncoupled from the secretion of neutrophil chemokines in M2 cells.

### Analysis of RNA sequencing datasets from breast cancer cell lines identifies correlations between *CXCL1*, *CXCL8*, TGF-β, and TNF-α pathways

To expand our findings to additional cancer cell lines *ex vivo* we analyzed the following two complementary datasets: i) the Harvard Medical School LINCS Breast Cancer Profiling Project (HMS LINCs dataset), which includes 35 breast cancer cell lines of known tumor subtype, and (ii) the Broad Institute Cancer Dependency Map Project (DepMap), containing a dataset of gene expression profiles for 1450 cancer cell lines of varying lineages, including 68 breast cancer cell lines.

**FIGURE 6:**
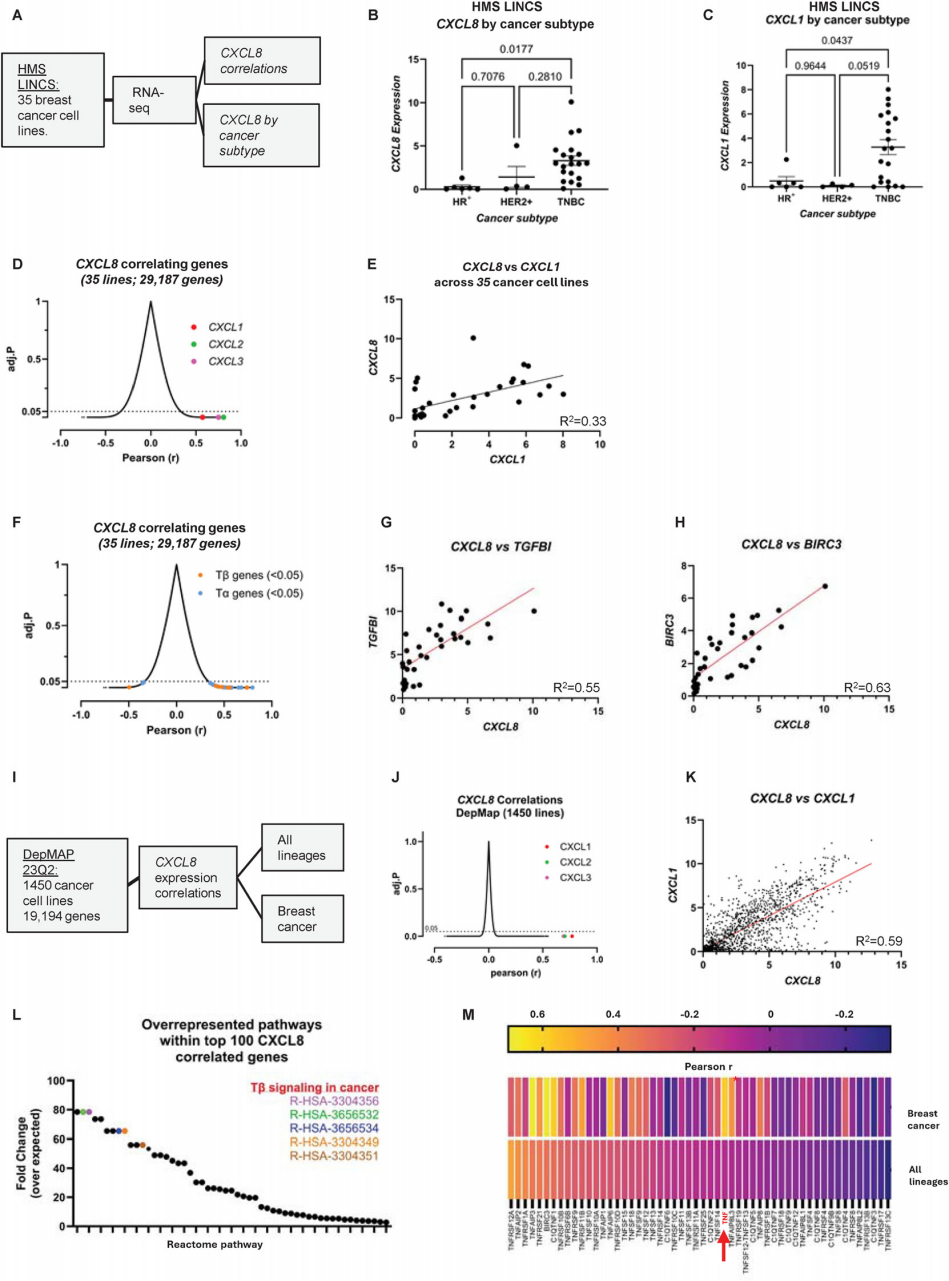
Computational analysis of gene expression datasets identifies the association between CXCL8 and members of TGF-β1/TNF-α pathways. (A) Workflow for the analysis of RNAseq dataset from the Harvard Medical School Breast Cancer Profiling Project (ID: 20352). (B and C) Expression level of CXCL8 (B) and CXCL1 (C) in HR^+^, HER2^+^, or TNBC cell lines available in the LINCs dataset. (D–H) Distribution of linear correlations (Pearson *r*) of all genes surveyed in the LINCs dataset against CXCL8 (D and F). Individual gene correlation between CXCL8 and CXCL1 (E), CXCL8 and TGFBI (TGFBI) (G) and CXCL8 and BIRC3 (H) across the 35 cell lines. (I) Workflow for the analysis of Broad institute DepMap gene expression profiles. (J and K) Distribution of linear correlations (Pearson *r*) between CXCL8 and all genes surveyed in the DepMap dataset, across 1450 available cell lines (J). Correlation between CXCL1 and CXCL8 across available cell lines (K). (L) Analysis of the top 100 CXCL8 correlation genes using panther overrepresentation assay. T-β–related pathways are marked in color and their Reactome pathway ID provided. (M) DepMap Analysis of CXCL8 correlations against T-α genes in breast cancer lines versus all lineages. The heatmap presents correlation coefficients of (Pearson *r*). TNF gene is highlighted in red.

First, we tracked *CXCL8* and *CXCL1* expressions across different breast cancer molecular subtypes using the HMS LINCS dataset (Figure 6A). We measured a significant increase in *CXCL8* and *CXCL1* mRNA transcripts in TNBC cell lines, compared with HR^+^ cell lines (Figure 6, B and C). We then tested whether this increase is due to a correlative relationship between *CXCL1* and *CXCL8* by calculating the correlation coefficient (reported as Pearson *r*) of all 29,187 surveyed genes against *CXCL8* across the 35 available cell lines. Supporting our findings in M2 cells, we found that the expression of CXCL8 correlates with CXCL1 (*R*^2^ = 0.33′), CXCL2 (*R*^2^ = 0.65) and CXCL3 (*R*^2^ = 0.53) (Figure 6, D and E). Within a total of 29,187 genes (across 35 cell lines), we found 4936 genes showing a significant correlation with CXCL8 (*P*_adj_ < 0.05). When focusing on the top correlating genes, considering both “correlation level” and “significance,” we found that several genes with key roles in the TGF-β signaling pathway are positively correlated with CXCL8 (Figure 6F), including TGFΒ-induced (TGFΒI, *R*^2^ = 0.55) (Figure 6G), TGFΒR2 (*R*^2^ = 0.54) and, to a lesser extent, SMAD3 (*R*^2^ = 0.31). Similarly, we identified members of the TNF-α signaling pathway to significantly correlate with *CXCL8* (Figure 6F), including *BIRC3* (*R*^2^ = 0.63) (Figure 6H), a TRAF1/2 binding and antiapoptotic protein that is upregulated with breast cancer metastasis (Ivagnès *et al.*, 2018; Srour *et al.*, 2020; Frazzi, 2021), TNF-α–induced protein 3 (*TNFAIP3*, *R*^2^ = 0.55), known to regulate NFκB and protect breast cancer cells from TNF-α–induced cell death (Lee *et al.*, 2019), and *CD137* (*TNFRSF9*, *R*^2^ = 0.46), which is associated with breast cancer metastasis (Jiang *et al.*, 2019) (the full list of *CXCL8*-correlating genes is given in Supplemental Table S2). Together, the HMS LINCS dataset analysis validates our experimental findings by identifying an association between *CXCL8* and *CXCL1* and key effectors of TGF-β and TNF-α signaling.

We next analyzed gene expression data from DepMap (Figure 6I). In agreement with our experimental data and the HMS LINCS analysis, we found that *CXCL8* expression is strongly correlated with *CXCL1* (*R*^2^ = 0.59), followed by *CXCL2* (*R*^2^ = 0.49) and *CXCL3* (*R*^2^ = 0.48), across all DepMap cancer cell lines (Figure 6, J and K). We then analyzed the top *CXCL8* correlating genes for statistical overrepresentation of signaling pathways using the Panther and Reactome datasets (Thomas *et al.*, 2022; Milacic *et al.*, 2024). We found that TGF-β–related genes show high fold-enrichment within the top 100 *CXCL8*-correlated genes across all cancer cell lines. Specifically, we noted a 78-fold increase above expected in “*Reactome pathway SMAD2/3 Phosphorylation Motif Mutants in Cancer*” (R-HSA-3304356), and the related “*TGFΒR1 KD Mutants in Cancer*” (R-HSA-3656532), “*Loss of Function of TGFΒR1 in Cancer*” (65-fold, R-HSA-3656534), “*Loss of Function of SMAD2/3 in Cancer*” (65-fold, R-HSA-3304349), and “*TGF-β Receptor Complex in Cancer*” (56-fold, R-HSA-3304351) (respectively, highlighted in magenta, green, blue, orange, and brown in Figure 6L). Interestingly, no TNF-α–related genes were overrepresented in the top *CXCL8-*correlated gene from DepMap. This contrasted with the HMS LINCS dataset, where only breast cancer cell lines are included. We therefore tested whether correlations between *CXCL8* expression and TNF-α or TGF-β–related genes were stronger in breast cancer cell lines relative to all the cell lines tested in DepMap. We found that overall correlations between TGF-β–related genes and *CXCL8* across all cell lines are not significantly different compared with breast cancer cell lines alone (*P* = 0.52, paired *t* test). These include *TGFBI* (all lines *R*^2^ = 0.2 vs. breast cancer *R*^2^ = 0.17), and *TGFBR2* (all lines *R*^2^ = 0.18 vs. breast cancer *R*^2^ = 0.19), as detailed in Supplemental Table S2 as Pearson *r* values. To test whether this trend persists for TNF-α family members, we examined *CXCL8* correlations with genes belonging to TNF Receptor Superfamily Members (TNFRSF), TNF-α–induced proteins (TNFAIP) and known TNF regulators (e.g., BIRC3). Interestingly, we identified many TNF-α pathway genes to associate significantly more strongly with *CXCL8* when comparing breast cancer cell lines with the entire dataset (*p* = 0.02, paired *t* test). Specifically, we observed significant associations between *CXCL8* and TNF-α genes (Figure 6M) in breast cancer cell lines. Besides, we measured an increased association between *CXCL8* and expression of *TNF* itself in breast cancer cell lines (*R*^2^ = 0.34) versus all cell lines (*R*^2^ = 0.01). The increased correlation with *CXCL8* expression was also observed for genes found in the HMS LINCS dataset, including *TNFAIP3*, *BIRC3*, and to a much lesser extent, *TNFRSF9* (see Supplemental Table S2). These findings complement and provide a broader context for the interpretation of our findings when analyzing the HMS LINCS dataset and further suggests a regulatory interaction between TGF-β, TNF-α, and *CXCL8* in breast cancer.

Finally, we tested whether *CXCL8* and *CXCL1* expressions correlate with EMT-associated genes, including *VIM* (gene for Vim), *SNAI1* (gene for Snail1), and *TWIST1* (gene for Twist1) in breast cancer cell lines using the HMS LINCS dataset. As expected, we measured a significantly higher *VIM* expression in the TNBC subtypes compared with others (Supplemental Figure S5A) (Grasset *et al.*, 2022). However, we observed low correlations for both *CXCL8* (*R*^2^ = 0.11) and *CXCL1* (*R*^2^ = 0.01) expressions with *VIM* in the TNBCs (Supplemental Figure S5B). Similarly, we measured weak correlations for both *CXCL8* and *CXCL1* expressions with *SNAI1* (*R*^2^ = 0.01 *CXCL8*, *R*^2^ = 0.01 *CXCL1*) and *TWIST1* (*R*^2^ = 0.03 *CXCL8*, *R*^2^ = 0.10 *CXCL1*) (Supplemental Figure S5, C and D) in the TNBCs. When we expanded our analysis to the DepMap dataset, we again observed weak to no correlations between *VIM* and *CXCL8* (*R*^2^ = 0.01) or *CXCL1* (*R*^2^ = 0.00) (Supplemental Figure S5, E and F). In contrast with the chemokines, VIM expression showed higher correlation with *CDH2* (gene for N-Cad) (*R*^2^ = 0.22) (Supplemental Figure S5G). When we focused on the 68 breast cancer cell lines from the DepMap dataset, we measured increased correlation of *VIM* expression with *CDH2* (*R*^2^ = 0.5) (Supplemental Figure S5J). However, as seen with the HMS dataset, we observed low correlations between VIM and either *CXCL8* (*R*^2^ = 0.11) or *CXCL1* (*R*^2^ = 0.13) (Supplemental Figure S5, H and I). These computational analyses further substantiate the lack of an association between the regulation of *CXCL8* and *CXCL1* expressions and EMT-associated changes in breast cancer cells and confirm the existence of a TGF-β/TNF-α regulatory axis for *CXCL1* and *CXCL8* that is, again, independent of EMT induction in breast cancer cells.

## DISCUSSION

In this study, we aimed to address whether TGF-β and TNF-α, two proinflammatory factors frequently detected in tumor niches, impact neutrophil recruitment to breast tumors. We found that dual-treatment with TGF-β1 and TNF-α enhances the secretion profile of neutrophil chemokines from human breast epithelial cell lines in a manner that is independent of the parallel induction in EMT markers. We also discovered that, unlike the expression of EMT markers, the TGF-β1/TNF-α–dependent upregulation of the neutrophil chemokines CXCL1 and CXCL8 relies on activation of the p38MAPK pathway. Finally, by analyzing transcriptomic databases for a wide array of human cancer cell lines representing different types of solid tumors, including breast, we uncovered significant correlations between *CXCL8* and a number of TGF-β1/TNF-α–associated genes. Furthermore, these computational analyses did not support a link between *CXCL8* or *CXCL1* and EMT-associated genes, thus broadening the significance of our studies involving the M2 breast epithelial cell line.

EMT-associated changes transform cancer cells into more aggressive and invasive types. For instance, it has been reported that progression to hybrid epithelial/mesenchymal states in TNBC tumors is important to drive cancer cell invasion (Grasset *et al.*, 2022). The presence of immune cells such as tumor-associated macrophages and other myeloid cells has been linked to expression of EMT markers in various cancer types (Suarez-Carmona *et al.*, 2017). Here, we set out to determine whether EMT-associated changes are involved in regulating neutrophil recruitment in breast cancer. We confirmed the occurrence of dramatic changes in EMT marker expression in M2 cells treated with TGF-β1/TNF-α. Treated M2 cells also acquired robust neutrophil recruitment activity. However, our study provides five lines of evidence showing that such EMT-associated changes are not essential for neutrophil recruitment. First, the ability of M2 cells to recruit neutrophils is greatly amplified with TNF-α treatment even though the latter fails to bring EMT-associated changes. Second, ectopic expression or depletion of snail and twist in M2 cells does not perturb the neutrophil recruitment ability of the CM obtained from these cells. Furthermore, depleting these TFs from aggressive breast cancer cell lines, for example, M4 and BT549, has no effect on their robust neutrophil recruitment activity. Third, the robust neutrophil recruitment activity of aggressive breast cancer cell lines, for example, M4, MDA-MB-231, and BT549, is not further amplified by TGF-β1/TNF-α treatment, even though the latter enhances expression of key EMT markers. Fourth, blocking p38MAPK activation drastically reduces the amount of neutrophil guidance cues in the CM obtained from TGF-β1/TNF-α dual-treated M2 cells and yet, mesenchymal morphology and levels of EMT markers are maintained under these conditions (except for Fn, which showed a partial decrease). Finally, transcriptomic analysis of large datasets of cancer cell lines of different lineages, including breast cancer provided additional evidence of a lack of correlation between neutrophil guidance cues (*CXCL8* and *CXCL1*) and EMT-associated genes. Together, these findings indicate that EMT-associated changes and amplified neutrophil recruitment are two distinct outcomes of TGF-β1/TNF-α stimulation.

The amplified neutrophil recruitment ability of treated M2 cells was accompanied by increases in secreted CXCL1 and CXCL8. CXCL8 is a potent ligand for CXCR1 and CXCR2, both of which are expressed in human neutrophils (Sabroe *et al.*, 2005; Ha *et al.*, 2017), whereas CXCL1 and CXCL2, which are members of the GRO family of chemokines, are CXCR2-specific ligands. All three chemokines have well-established role in mediating neutrophil recruitment to tumors (Acharyya *et al.*, 2012; Kaunisto *et al.*, 2015; Manfroi *et al.*, 2017), and the increased expression of all three chemokines is associated with more aggressive tumors, including glioblastoma, breast and colorectal cancer, and malignant melanoma (Varney *et al.*, 2006; Ha *et al.*, 2017; De Boeck *et al.*, 2020; Hu *et al.*, 2021). We also found that expression of *CXCL8* mRNA is significantly higher in aggressive TNBC cell lines compared with HR^+^ ones. Furthermore, we discovered that the expression of *CXCL8* is highly correlated with that of *CXCL1* transcripts in breast cancer cell lines. We previously described a key role for CXCL1/2 chemokines secreted by aggressive breast cancer cell lines to induce robust neutrophil recruitment (SenGupta *et al.*, 2021b). In the current study, we found that neutralizing CXCL8 action did not completely abrogate the ability of CM from TGF-β1/TNF-α–treated M2 cells to induce a neutrophil chemotactic response. Therefore, CXCL1/2 and CXCL8 may act in a synergistic manner to control neutrophil trafficking to breast tumors.

Although the role of TGF-β1/TNF-α in regulating the expression of EMT markers is well-established, how the combined action of the two factors impacts immune cell recruitment to tumors has been largely unexplored. TNF-α induces the expression and secretion of CXCL1, CXCL10 and several CCL chemokines in immune cells, endothelial cells and cancer cells (Hornung *et al.*, 2000; Lo *et al.*, 2014; Kochumon *et al.*, 2021). TGF-β controls chemokine availability either by inducing or suppressing the expression and secretion of chemokines (Wang *et al.*, 2009; Rodríguez *et al.*, 2015; Haider *et al.*, 2019). For instance, TGF-β promotes neutrophil recruitment by upregulating the expression and secretion of CXCL5 in hepatocellular carcinoma (Haider *et al.*, 2019), while it suppresses the secretion of CXCL1 by mesenchymal stromal cells (Rodríguez *et al.*, 2015). Because we previously reported that CXCL5, CCL2, CCL3, and CCL5 occur at low levels in CM collected from aggressive breast cancer cell lines, here we focused on other abundantly secreted chemokines (SenGupta *et al.*, 2021b). When we dissected the effect of TGF-β1 and TNF-α through individual versus dual-treatment, we found interesting changes in the chemokine secretion profiles of M2 cells. Treatment with TNF-α was effective at inducing CXCL1 and CXCL8, while TGF-β1 was not. Treatment with TGF-β1 suppressed CXCL1 secretion from the cotreated M2 cells, supporting previous findings (Rodríguez *et al.*, 2015). On the other hand, TGF-β1 boosted the amount of secreted CXCL8 in cotreated M2 cells, suggesting cross-talk between the two signaling pathways. Additionally, the positive correlation we measured between CXCL8 and a number of TGF-β1 and TNF-α–associated genes in a large dataset of breast cancer cell lines validates our in vitro findings on a positive regulation of CXCL8 by TGF-β1 and TNF-α. Our findings also suggest that the relative availability of TGF-β1 and TNF-α in the tumor niche controls the abundance of key neutrophil-recruiting chemokines, and thus regulates the degree of neutrophil recruitment to breast tumors. Unlike in M2 cells, we found that TGF-β1/TNF-α treatment does not improve the neutrophil-recruiting ability of TNBC cell lines. As these cells produce massive amounts of neutrophil-recruiting chemokines (SenGupta *et al.*, 2021b), we reason that chemokine receptor signaling is saturated and therefore not mediating chemotactic responses. Whether TGF-β1/TNF-α treatment further modifies the robust expression and secretion of the chemokines from TNBCs remains to be determined.

To seek mechanistic insight, we evaluated the status of signaling pathways known to impact EMT programs, including Smad3, NF-κβ, p38MAPK, ERK, JNK, and AKT (Gonzalez and Medici, 2014). Of all the pathways tested, we found more robust activation of NF-κβ and p38MAPK pathways with dual TGF-β1/TNF-α treatment compared with single treatments. Yet, we found a key and specific role for p38MAPK-mediated signaling in the secretion of CXCL1 and CXCL8 from the CM isolated from TGF-β1/TNF-α–treated M2 cells. Active p38MAPK is already known to regulate the production of a number of proinflammatory cytokines and chemokines from both immune cells and nonimmune cells, such as epithelial and endothelial cells (Hashimoto *et al.*, 1999; Parhar *et al.*, 2003; Petrova *et al.*, 2020; Canovas and Nebreda, 2021). Such regulation can occur through the modulation of NF-κB activation or by promoting mRNA stability (Baeza-Raja and Muñoz-Cánoves, 2004; Suswam *et al.*, 2008). Furthermore, a regulatory effect of p38MAPK on chemokines and Fn expression has been reported in breast cancer (Limoge *et al.*, 2017). Our studies shed additional light into this. Indeed, we found that, while Vim and N-Cad expression levels remained unchanged, the presence of a pan-P38MAPK inhibitor (Kuma *et al.*, 2005) resulted in reduced secretion of CXCL1 and CXCL8 as well as a decrease in Fn expression. Other proinflammatory factors, for example, IL-1β, have been shown to regulate Fn expression (Tunali *et al.*, 2023). Our observation of a partial reduction in Fn expression following inhibitor treatment suggests that Fn can be regulated by EMT-independent mechanisms. Future studies should determine which isoform/s of p38MAPK are specifically involved in regulating the secretion of neutrophil guidance cues with the TGF-β1/TNF-α treatment and elucidate the mechanisms underlying the regulation.

Neutrophil recruitment to tumors enhances breast cancer progression by increasing tumor angiogenesis, promoting EMT signatures and cancer aggressiveness (Martins-Cardoso *et al.*, 2020; Yu *et al.*, 2024; Camargo *et al.*, 2025). Elucidating the mechanisms underlying neutrophil recruitment in breast tumors, therefore, is of immense clinical value. The findings we report here establish that the regulation of neutrophil recruitment by TGF-β1/TNF-α is mediated via the secretion of CXCL1/8 and can occur independently of EMT. We further establish that the TGF-β1/TNF-α/p38MAPK signaling axis is a potential predictor of neutrophil recruitment in the breast tumor niche. Future studies will investigate whether the TGF-β1/TNF-α axis also regulates neutrophil recruitment in poorly aggressive breast tumors using HR^+^ breast cancer cell lines that show minimum neutrophil recruitment abilities (SenGupta *et al.*, 2021b) and whether tumor-derived CXCL1/8 modulates neutrophil function to promote cancer cell aggressiveness.

## MATERIALS AND METHODS

### Materials

TGF-β1 from R&D Systems (7754-BH-005) or Peprotech (100-21); TNF-α from Peprotech (300-01A) or Prospec (cyt-114); human recombinant CXCL8 from ProSpec (CHM349); Formyl-methionyl-leucyl-phenylalanine (fMLF) from Sigma-Aldrich (F3506); DMSO from Sigma-Aldrich; DMPM from Cayman Chemicals (10460), human CXCL-8 antibody from R&D systems (MAB208-100), and isotype antibody from Invitrogen (02-6100 or 31903) were used in the study.

### Isolation of human neutrophils

Blood was obtained from healthy human male and female subjects ages 19 to 65 years, through the Platelet Pharmacology and Physiology Core at the University of Michigan. The Core maintains a blanket IRB for basic science studies, where HIPAA information is not required. Therefore, although the IRB-approved Core enrolls healthy subjects that conform to the protection of human subject standards, we did not have access to this information. The samples that we received were fully deidentified. Neutrophils were purified using dextran-based sedimentation followed by histopaque-based density gradient centrifugation as described previously (SenGupta *et al.*, 2021b). Cells were more than 99% viable immediately following isolation. To address donor-to-donor variability of neutrophil response, cells were routinely tested for minimum basal activity and a robust response to fMLF stimulation as described before (SenGupta *et al.*, 2021b).

### Cell lines

We used a panel of 10 cell lines of human breast epithelial origin described in Table 1. MCF10A (M1), MCF10AT (M2), and MCF10CA1a (M4) were obtained from the Karmanos Research Institute. The human TNBC cell lines BT549 and MDA-MB-231 were purchased from the ATCC. M2-GFP, M2-snail, M2-twist, M2 twist KO, M4 twist KO, and BT549 twist KO cell lines were generated and validated in the laboratory for the expression of snail, twist, and GFP (see Supplemental Figure S1). All cell lines were maintained using representative culture media (Table 1) and incubated at 37°C in 5% CO_2_ humidified tissue culture incubator. All cell lines tested negative for *Mycoplasma* contamination using the Mycoalert detection kit (Lonza). All cell lines were verified and authenticated based on short tandem repeat markers (Biomedical Research Core Facilities).

**Table 1.** Cell lines and culture media used on the study.

Cell lines	Culture media	Supplemented components
M1	DMEM/F12 (Gibco)	5% heat inactivated horse serum (HI HS), 100 ng/ml cholera toxin, 20 ng/mL EGF, 0.01 mg/ml insulin, 500 ng/ml hydrocortisone
M2, M2 GFP, M2 snail, M2 twist	DMEM/F12 (Gibco)	5% HI HS, 100 ng/ml cholera toxin, 20 ng/mL EGF, 0.01 mg/ml insulin, 500 ng/ml hydrocortisone
M4, M4 twist KO	DMEM/F12 (Gibco)	5% HI HS
BT549, BT549 twist KO, BT549 snail1 sh	RPMI (Gibco)	10% FBS
MDA-MB-231	DMEM (Gibco)	10% FBS

### Plasmid constructs

The pBABE-Puro-twist, -eGFP-snail6SA, or -GFP constructs were used to generate stable M2 cell lines expressing twist, eGFP-snail6SA or GFP control. Twist from pWZL-Blast-twist construct (a kind gift from Jing Yang, UCSD) or eGFP-snail6SA from the eGFP-Snail6SA construct [Addgene plasmid #16228, deposited by Mien-Chie Hung; (Zhou *et al.*, 2004)] was subcloned into pBABE-Puro backbone from the pTK92 [Addgene plasmid #46356, deposited by Iain Cheeseman; (Kiyomitsu and Cheeseman, 2013)]. pBABE-GFP was generated from pTK92. For CRISPR-based KOs, twist1 sgRNA CGGGAGTCCGCAGTCTTACGAGG was cloned into pLentiCRISPRv2 construct as described [Addgene plasmid #52961, deposited by Feng Zhang, (Sanjana *et al.*, 2014)]. The pGipZ V3LHS_328731 and pGipZ scramble (SCR) constructs, obtained from the Vector core at the University of Michigan, were used to generate stable BT549 cell lines expressing snail1 and SCR shRNA, respectively.

### Stable cell line generation

For generating M2 cell lines stably expressing exogenous proteins, pBABE-based constructs were transfected using Lipofectamine 3000 (Invitrogen) into Phoenix 293T packaging cells. Retroviral particles released from the packaging cells were collected 48 h posttransfection and concentrated using PEG-8000 (V3011, Promega), and then used to infect M2 cells in the presence of 10 µg/ml polybrene. The clones expressing the constructs were then selected in 2 µg/ml puromycin and verified using Western blotting and fluorescence microcopy. For generating CRISPR-based KO or shRNA-based KD cell lines, pLentiCRISPRv2 construct carrying twist1 sgRNA or pGipZ construct carrying snail1/SCR shRNA was produced by the Vector Core at the University of Michigan and were infected as described above. Clones expressing the construct were selected with puromycin and verified by Western blotting and sequencing.

### Treatment for harvesting CM

Cell lines were seeded at a density of 0. 03X10^6^/ml in 2 ml volume of complete medium in each well of a 6-well tissue culture plate and incubated overnight to allow cell adhesion. The following day, the cells were either left untreated, or treated with 20 ng/ml TGF-β1 and 100 ng/ml TNF-α for 72 h at 37°C. For inhibiting p38MAPK phosphorylation, cells were pretreated with DMPM or vehicle control for 2 h followed by TGF-β1/TNF-α treatment. The culture medium was then removed, and cells were gently washed twice with calcium and magnesium-free sterile DPBS (Life Technologies) to remove left-over serum-containing media. Cells were then incubated with fresh medium without serum and incubated for an additional 48 h. The media were harvested and filtered through 0.22-µm membrane filters to remove dead cell debris. Aliquots were frozen at –30°C until analyzed.

### Harvesting CM from WT versus KO or KD cancer cell lines

To generate CM, breast cancer cell lines were seeded at a density of 0.15X10^6^/ml in 2 ml (6-well tissue culture plate) of complete medium for 24 h, at which point they reached ∼70% confluence. The culture medium was then removed, and cells were gently washed as mentioned above and incubated with serum-free fresh medium for an additional 48 h. The CM was collected and stored as described above.

### Bright-field microscopy

To assess morphological changes with treatment, cell lines were treated as before for 72 h. Bright-field images in three randomly selected fields per condition were captured using a ×10 objective lens on a Zeiss Axiovert microscope.

### Immunofluorescence microscopy

For analyzing changes in EMT markers and cytosolic/nuclear area, cells were seeded at a density of 0.04X10^6^/ml in 0.3 ml media in an 8-well glass-bottom chamber coated with Type 1 collagen (Purecol) (100 µg/mL). For assessing nuclear translocation of p65, cells were seeded at a density of 0.2X10^6^/ml in 0.3 ml media in a coated chamber as described above. The following day, the cells were treated as before for 72 h (for markers) or 30 min (for p65), fixed with 4% paraformaldehyde (Electron Microscopy Sciences) for 15 min at 37°C, washed in DPBS (Life Technologies) and permeabilized/blocked with blocking solution (0.3% TritonX-100 and 3% BSA-containing DPBS) for 1 h at room temperature (RT). Cells were then stained with primary antibodies against E-Cad, N-Cad, Fn, Vim, or p65 diluted in the blocking solution supplemented with 1% goat serum and incubated at 4°C for overnight. The dilutions used for the primary antibodies and their sources are presented in Table 2. The next day, cells were washed in DPBS and stained with AF488 or AF568 fluorochrome–conjugated goat anti-mouse and anti-rabbit secondary antibodies (dilution 1:500, Invitrogen), as well as DAPI (D9542, Sigma-Aldrich) and/or Phalloidin (P1951, Sigma-Aldrich). Cells were imaged using a ×63 or ×20 objective lens on a Zeiss LSM880 Airyscan confocal microscope. Cells in three to five different fields across the well were captured randomly per condition in each experiment.

**Table 2.** Antibody information for western blotting and IF.

Antibody against	Species	Dilution WB	Dilution IF	Distributor/catalog number
Vim	Rabbit	1:500	1:500	Cell Signaling Technology, 5741S
E-Cad	Mouse	1:10,000	1:500	BD Biosciences, 610181
N-Cad	Mouse	1:500		Santa Cruz Biotechnology, sc-59987
N-Cad	Rabbit		1:500	Proteintech 22018-1-AP
Snail	Rabbit	1:500	1:500	Cell Signaling, 3879S
Twist	Mouse	1:650	1:500	Santa Cruz Biotechnology, sc- 81417
Fibronectin	Mouse	1:500	1:500	Santa Cruz Biotechnology, sc-271098
NFkB p65	Rabbit		1:100	Invitrogen, 14-6731-81
P-SMAD3	Rabbit	1:1000		Boster Bio P00059-1
P-p38MAPK (Thr180/Tyr182)	Rabbit	1:1000		Cell Signaling Technology, 4511
P-ERK (Thr202/Tyr204)	Rabbit	1:2000		Cell Signaling Technology, 4370
P-JNK (Thr183/Tyr185)	Rabbit	1:1000		Cell Signaling Technology, 9251
P-AKT (Thr308)	Rabbit	1:1000		Cell Signaling Technology, 4056
SMAD3	Rabbit	1:1000		Cell Signaling Technology, 9513
p38MAPK	Rabbit	1:1000		Cell Signaling Technology, 9212
ERK	Rabbit	1:1000		Cell Signaling Technology, 4695
JNK	Rabbit	1:1000		Cell Signaling Technology, 9252
AKT	Rabbit	1:1000		Cell Signaling Technology, 4691
GAPDH	Mouse	1:1000		Santa Cruz Biotechnology, sc-166574
Alpha-tubulin	Mouse	1:2000		Proteintech, HRP-66031
GFP	Rabbit	1:1000		Abcam, ab290

### Western blotting

Cells following treatments in a 6-well plate were lysed using radio immunoprecipitation assay buffer supplemented with Halt protease inhibitor (Thermo Fisher Scientific, #87786). Collected lysates were clarified by centrifugation at 13,000 rpm for 15 min at 4°C. The protein contents of each sample were then estimated using the BCA Protein Assay Kit (Thermo Fisher Scientific, #23225). An equal amount of protein was resolved by 10% SDS–PAGE transferred onto polyvinylidene fluoride (PVDF) or nitrocellulose membranes (Millipore), blocked with 5% nonfat dry milk for 1 h and probed with primary antibodies against phospho- and total p44/42 MAPK (ERK1/2), p38MAPK, JNK, AKT, SMAD3, GAPDH, and α-Tubulin. The dilutions used for the primary antibodies and the sources are described in Table 2. Bands were visualized using horseradish peroxidase (HRP)-conjugated secondary antibodies (dilution 1:8000, Jackson ImmunoResearch), SuperSignal West Pico PLUS Chemiluminescent Substrate, and a C600 digital imaging system (Azure). Integrated density for each band was measured using ImageJ software.

### Transwell assay

To quantify the ability of CM to induce neutrophil migration, Transwell migration chambers with membrane inserts of 3-µm diameter pore size (Greiner Bio-One) were used. Both inserts and bottom wells were coated with 2% tissue culture grade BSA for 1 h at 37°C to prevent strong neutrophil adhesion. Coated inserts and wells were rinsed with DPBS twice to remove residual BSA. Freshly isolated neutrophils resuspended in Ca^++^, Mg^++^ HBSS (Life Technologies) at a density of 4X10^6^/ml were seeded onto the inserts (100 µl) and placed in a 24-well plate. Control chemoattractants or CM were gently added in 600 µl volume to the bottom wells of the 24-well plate. For the neutralization assay, CM or chemoattractant was incubated with 5 µg/ml antibodies for 30 min at 37°C with gentle rotation before they were added to the bottom wells. Migration was allowed to take place in 37°C/5% CO_2_ for 2 h. The percentage of neutrophils migrated to the bottom chamber was calculated from the cell counts obtained using a hemocytometer.

### Cancer cell migration and invasion assay

Endpoint migration and invasion assays were performed using a Transwell system in 24-well plates as described before (SenGupta *et al.*, 2021b). Briefly, fluoroBlock filter inserts (351152, Corning) with 8 micron (um) pore size were left uncoated for the migration assays or coated for the invasion assays with 0.2 mg/ml type I bovine collagen PureCol of which the pH was neutralized to 7.2 to 7.4 using sodium bicarbonate. Cell lines were serum-starved for 24 h and then seeded onto the inserts at 0.5 × 10^5^ cells/ml in 100 µl of serum-free media. Cells were allowed to migrate toward the bottom chamber containing 500 microliter (ul) of full-serum media as the chemoattractant or serum-free media as the negative control. After 24 h incubation at 37°C/5% CO_2_, the inserts were transferred to a fresh 24-well plate with black walls containing 500 µl of 4 µM Calcein AM (Biotium) in Ca^++^, Mg^++^ HBSS per well. Cells were incubated for 1 h at 37°C, and the fluorescence reading of migrated/invaded cells was measured from the bottom at wavelengths of 495/515 nm (Excitation/Emission) by a SpectraMax M5 Multi-Mode Microplate Reader (Molecular Devices). The fluorescence value of the negative control was subtracted from the fluorescence value obtained for the full-serum media condition.

### ELISA Assay

The presence of neutrophil-recruiting chemokines; CXCL1, CXCL2, CXCL8, and activating factor; TGF-β1 in the CM was quantified by ELISA through the Rogel Cancer Center Immunology Core. Samples were tested with dilutions in quadruplicate. The protein concentration for each target analyte was quantified by comparing the colorimetric signal from the sample with the individual standard curve generated by known concentrations of each protein.

### Image quantification and data representation

CellProfiler (ver.4.2.5) was used to segment cells with “IdentifySecondaryObjects” module and nuclei with “IdentifyPrimaryObjects” module based on “Minimum Cross-Entropy or Otsu” algorithms for intensity thresholding. Cytosol was segmented by subtracting nuclei from cell with “IdentifyTertiaryOjects” module. The integrated intensities of EMT markers were quantified with “MeasureObjectIntensity” module using “Minimum Cross-Entropy or Otsu” algorithms for intensity thresholding. Cytosolic area as presented in Figure 1Bi was quantified from the maximum intensity projections (MIP) of the z-stacks of phalloidin-TRITC and DAPI stained cells as in Figure 1, Aiii and iv. Nuclear area as presented in Figure 1Bii was quantified from representative *z*-stacks of DAPI-stained nuclei as in Figure 1, Av and vi. Ratio of cortex to cortex-free cytosolic signal intensities for E-Cad were calculated from representative z-stacks of E-Cad/Vim/DAPI-stained cells as in Figure 1, Av and vi. Signals of Fn, Vim, and N-Cad were quantified from a representative *z*-stack of individual images as in Figure 1, Bv–viii or the MIP of the z-stacks of individual images as in Figure 5, Civ–ix. For measuring nuclear translocation of p65, ratio of nuclear to cytosolic signal intensities for p65 were calculated from the MIPs of the z-stacks of phalloidin-TRITC/p65/DAPI-stained cells as in Figure 4B.

### Gene expression dataset analysis

Havard Medical School LINCS (Library of Integrated Network-based Cellular Signatures) dataset ID:20348 was downloaded through the LINCS DB database website (March 2023). Cell lines were then annotated by their respective tumor subtype. Correlations between all genes in the dataset were calculated using R v.4.2.2, cor function and *P* values adjusted using the FDR method.

DepMap 23Q2 mRNA expression data (1450 cell lines) were downloaded through the DepMap Portal (https://depmap.org/portal) and analyzed using R v.4.2.2. Cell types were then annotated by their lineage (“OncotreeLineage”) to identify differential correlations between breast cancer cell lines versus the entire database. All correlations were calculated using *r* and *P* values adjusted using the FDR method. The top 100 correlating genes were uploaded to the PANTHER Classification System API (https://www.pantherdb.org/, (Thomas *et al.*, 2022), and were analyzed for statistical overrepresentation test (PMID 30804569), using Reactome pathways annotation (Milacic *et al.*, 2024). Pathways with FDR *P*_adj_ < 0.05 were plotted for their fold-enrichment using GraphPad Prism.

### Statistical analysis

GraphPad Prism software was used for data plotting and conducting statistical analysis by tests that are described in the respective figure legends along with the size of the samples. Tests used included two-tailed paired *t* test, unpaired *t* test, 1-way ANOVA with Dunnett's multiple comparisons test or two-way ANOVA with Sidak's or Turkey multiple comparisons test.

## Supporting information







## Data Availability

All the raw data presented have been provided within Supplemental Materials for the respective figures. The raw microscopy images are available from the corresponding author upon reasonable request.

## Abbreviations used:

AKT: phosphatidylinositol 3-kinase (PI3K)-protein kinase B
CM: conditioned media
DMPM: doramapimod
E-Cad: e-cadherin
EMT: epithelial-to-mesenchymal transitions
ERK: extracellular signal–regulated kinase
Fn: fibronectin
fMLF: formyl-methionyl-leucyl-phenylalanine
HR^+^: hormone receptor–positive
JNK: C-Jun NH2-terminal kinase
KD: knockdown
KO: knockout
MAPK: mitogen-activated protein kinase
N-Cad: n-cadherin
NF-κB: nuclear factor κ-light-chain enhancer of activated B cells
TF: transcription factor
TGF-β1: transforming growth factor-β1
TNBC: triple-negative breast cancer
TNF-α: tumor necrosis factor-α
Vim: vimentin.
