# Supplementary material for: TGFβ1–TNFα-regulated secretion of neutrophil chemokines is independent of epithelial–mesenchymal transition in breast tumor cells
Source: Mol Biol Cell. 2025 Sep 12;36(10):ar122. doi: 10.1091/mbc.E25-07-0340 (PMC12483376; doi:10.1091/mbc.E25-07-0340)
Supplement: Supplementary file 1 [file mbc-36-ar122-s001.pdf]

Supplemental Materials

Molecular Biology of the Cell

SenGupta *et al*.

SUPPLEMENTARY INFORMATION

TGFβ1-TNFα regulated secretion of neutrophil chemokines is independent of epithelial-mesenchymal transition in breast tumor cells

Shuvasree SenGupta[1,2], Erez Cohen[3], Joseph Serrenho[2], Kaleb Ott[1,4],

Pierre A. Coulombe[3,5,6], Carole A. Parent[1,2,3,5*]

[1] Life Sciences Institute, University of Michigan, Ann Arbor, MI

[2] Department of Pharmacology, University of Michigan Medical School, Ann Arbor, MI

[3] Department of Cell & Developmental Biology, University of Michigan Medical School, Ann Arbor, MI

[4] Undergraduate Research Opportunity Program, University of Michigan, Ann Arbor, MI

[5] Rogel Cancer Center, University of Michigan Medical School, Ann Arbor, MI

[6] Department of Dermatology, University of Michigan Medical School, Ann Arbor, MI

Figure S1

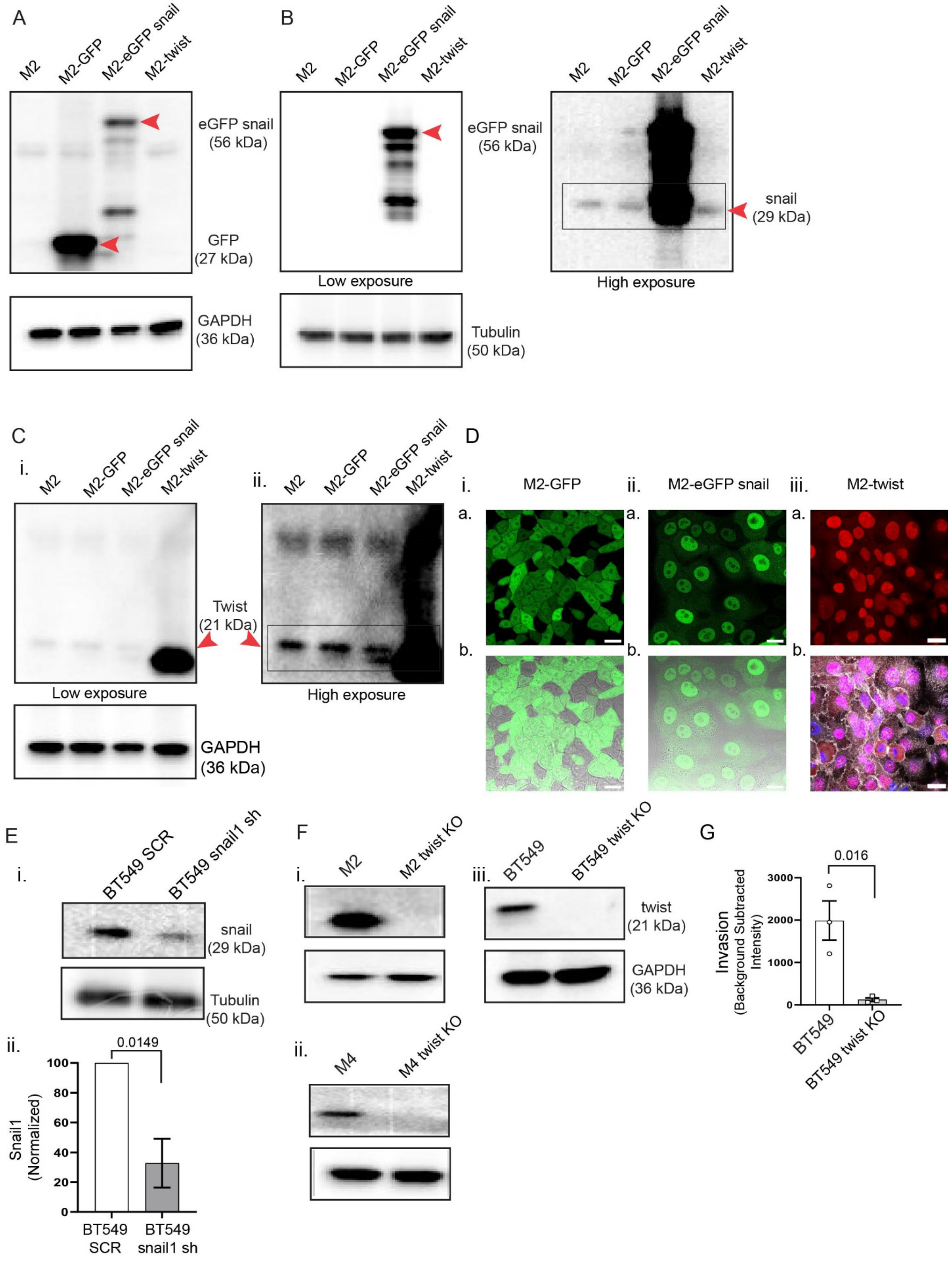

A
M2
M2-GFP
M2-eGFP snail
M2-twist
eGFP snail (56 kDa)
GFP (27 kDa)
GAPDH (36 kDa)
B
M2
M2-GFP
M2-eGFP snail
M2-twist
eGFP snail (56 kDa)
Low exposure
Tubulin (50 kDa)
snail (29 kDa)
High exposure
C
i.
M2
M2-GFP
M2-eGFP snail
M2-twist
Twist (21 kDa)
Low exposure
GAPDH (36 kDa)
ii.
High exposure
D
i. M2-GFP
ii. M2-eGFP snail
iii. M2-twist
a.
b.
E
i.
BT549 SCR
BT549 snail1 sh
snail (29 kDa)
Tubulin (50 kDa)
ii.
0.0149
Snail1 (Normalized)
0
20
40
60
80
100
BT549 SCR
BT549 snail1 sh
F
i.
M2
M2 twist KO
ii.
M4
M4 twist KO
iii.
BT549
BT549 twist KO
twist (21 kDa)
GAPDH (36 kDa)
G
0.016
Invasion (Background Subtracted Intensity)
0
1000
2000
3000
BT549
BT549 twist KO

Figure S1. Snail and twist expression in engineered cell lines

A-C. Representative western blots showing the expression of GFP or eGFP-snail with anti-GFP (A) or anti-snail Ab (B; low (left) or high (right) exposures) and twist with anti-twist Ab (C; low (left) or high (right) exposures) in the respective cell lines (n=2).

D. Representative IF images (n=2) showing the GFP signal in M2 GFP cells (ia) and M2-eGFP snail cells (iia) or of fixed M2-twist cells stained with twist Ab (red) (iiia). IF images merged with corresponding bright field images of M2 GFP cells (ib) and M2-eGFP snail cells (iib) or IF image of DAPI stained nucleus (blue), phalloidin-TRITC (grey) stained F-actin, and twist (red) in M2-twist cells (iiib). Scale bar 20 μm.

E. Representative western blot showing the expression of snail in BT549 snail sh compared to SCR cell lines (i). Bars showing band intensities of snail normalized to the loading control (mean values +/- SEM from n=3).

F. Representative western blot showing the expression of twist from M2 (i), M4 (ii), and BT549 (iii) twist KO cell lines (n=2).

G. Graphs showing the invasion of BT549 WT and twist KO cell lines (mean values +/- SEM from n=3). P values were determined using unpaired t test (Eii and G).

Figure S2

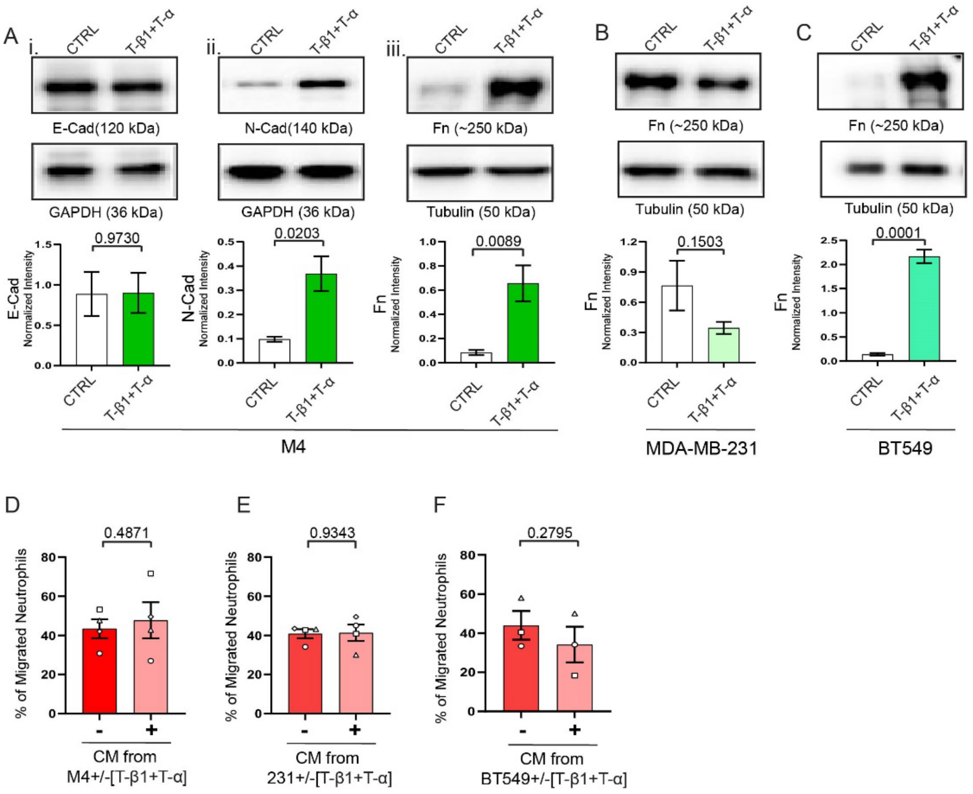

Figure S2. TGF-β1/TNF-α treatment does not boost neutrophil recruiting activity of TNBC cells

A-C. Top: Representative western blots showing E-Cad (Ai), N-Cad (Aiii) or Fn (Aiii, B,C) expression of CTRL or T-β1/T-α treated M4, MDA-MB-231 and BT549 cells. Bottom: Graphs showing band intensities of the respective markers normalized to loading controls are presented (mean values +/- SEM from n=3-4).

D-F. Graphs depicting the percentage of neutrophils that migrated into the bottom chamber of transwells containing equal volume of CM from CTRL or T-β1/T-α treated TNBCs (mean +/- SEM form n=3-4). Each dot represents response of neutrophils from an independent donor.

P values were obtained using Unpaired (A-C) or Paired (D-F) t test.

Figure S3

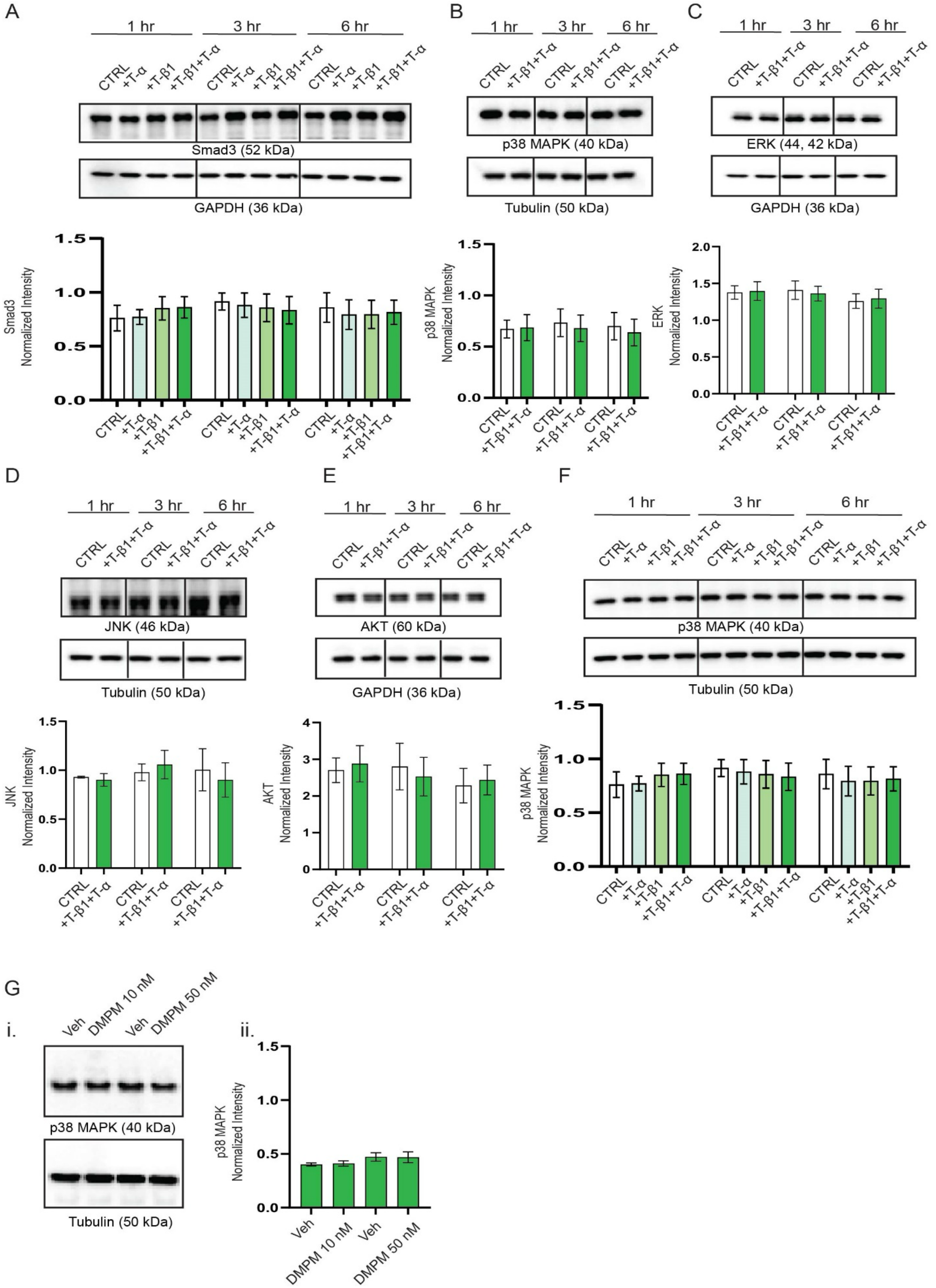
A
1 hr
3 hr
6 hr
CTRL
+T-α
+T-β1
+T-β1+T-α
Smad3 (52 kDa)
GAPDH (36 kDa)
Smad3 Normalized Intensity
B
p38 MAPK (40 kDa)
Tubulin (50 kDa)
p38 MAPK Normalized Intensity
C
ERK (44, 42 kDa)
GAPDH (36 kDa)
ERK Normalized Intensity
D
JNK (46 kDa)
Tubulin (50 kDa)
JNK Normalized Intensity
E
AKT (60 kDa)
GAPDH (36 kDa)
AKT Normalized Intensity
F
p38 MAPK (40 kDa)
Tubulin (50 kDa)
p38 MAPK Normalized Intensity
G
i.
ii.
Veh
DMPM 10 nM
DMPM 50 nM
p38 MAPK (40 kDa)
Tubulin (50 kDa)
p38 MAPK Normalized Intensity

Figure S3. TGF-β1/TNF-α treatment does not affect total protein levels of the activated signaling pathways in M2 cells

A-F. Representative western blots showing Smad3 (A), p38MAPK (B, F), ERK (C), JNK (D) or AKT (E) expression of CTRL M2 cells or M2 cells treated with T-β1 or T-α or T-β1+T-α over the course of 1, 3, and 6 hrs of treatments. Graphs depicting band intensities of specified proteins normalized to loading control (mean +/- SEM from n≥3).

G. (i) Representative western blots showing p38MAPK expression of M2 cells pre-treated with DMPM or vehicle control and stimulated with T-β1+T-α for 72 hrs. (ii) Graph depicting band intensities of p38 MAPK normalized to loading control (mean +/- SEM from n=3).

Figure S4

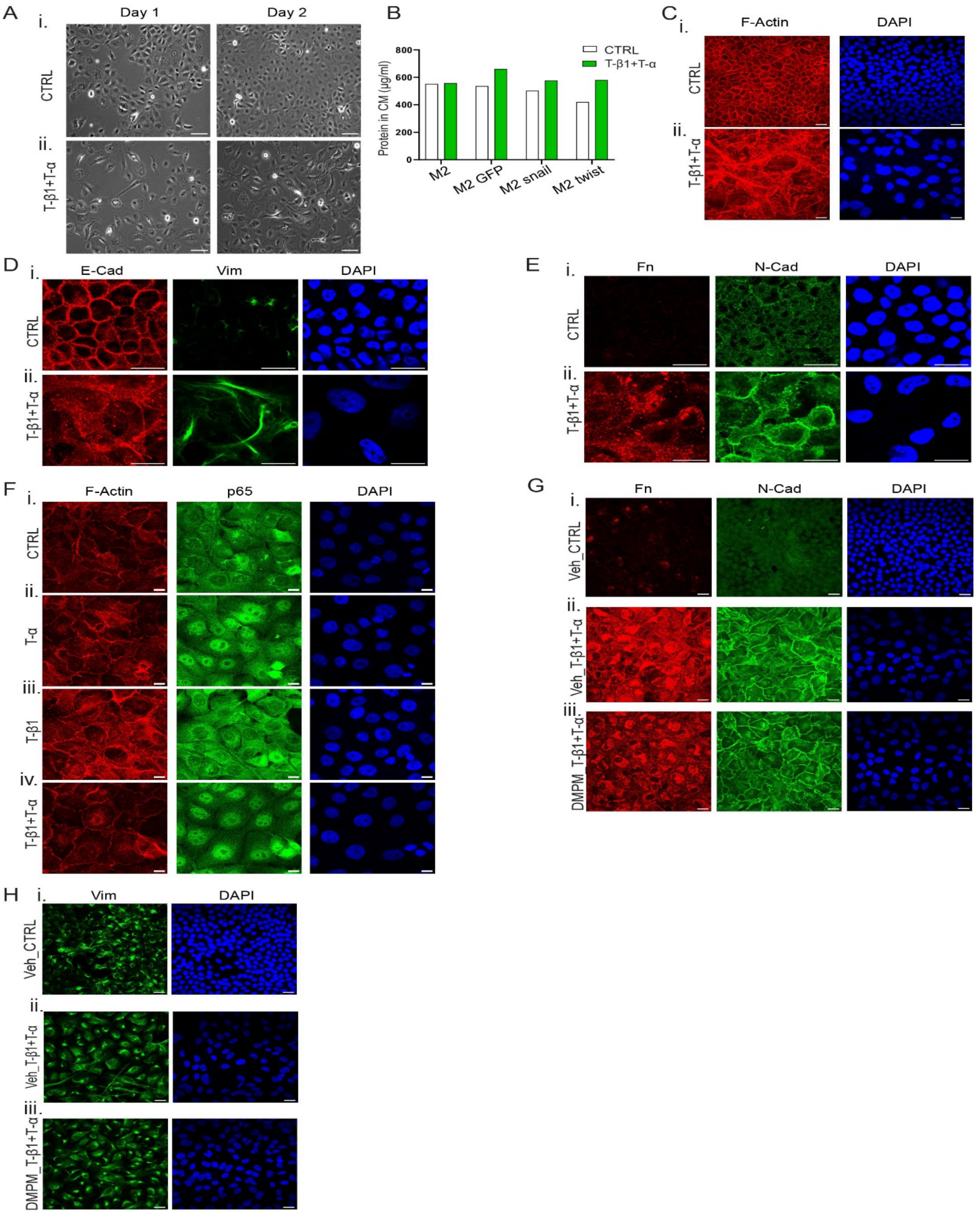
A
i.
Day 1
Day 2
CTRL
ii.
T-β1+T-α
B
Protein in CM (µg/ml)
800
600
400
200
0
CTRL
T-β1+T-α
M2
M2 GFP
M2 snail
M2 twist
C
i.
F-Actin
DAPI
CTRL
ii.
T-β1+T-α
D
i.
E-Cad
Vim
DAPI
CTRL
ii.
T-β1+T-α
E
i.
Fn
N-Cad
DAPI
CTRL
ii.
T-β1+T-α
F
i.
F-Actin
p65
DAPI
CTRL
ii.
T-α
iii.
T-β1
iv.
T-β1+T-α
G
i.
Fn
N-Cad
DAPI
Veh_CTRL
ii.
Veh_T-β1+T-α
iii.
DMPM_T-β1+T-α
H
i.
Vim
DAPI
Veh_CTRL
ii.
Veh_T-β1+T-α
iii.
DMPM_T-β1+T-α

Figure S4. Morphology and EMT markers in M2 cells treated with TGF-β1/TNF-α

A. Representative sub-confluent and confluent bright field images (i, ii) of CTRL M2 cells (i) or M2 cells treated with T-β1+ T-α for 24 and 48 hrs (ii). Scale bar 50 μm.

B. Graph showing the protein content in CM collected from CTRL or T-β1+ T-α treated M2, M2 GFP, M2 snail, and M2 twist cells.

C-E. Representative individual channel IF images of CTRL or M2 cells treated with T-β1+ T-α for 72 hrs for merged images presented in Fig 1A. Individual channel images from Airyscan confocal microscopy showing MIP (C) or a single z image (D, E) of fixed M2 cells stained for F-actin with phalloidin-TRITC (C; see Fig. 1Aiii&iv), E-Cad/Vim (D; see Fig. 1Av&vi), N-Cad/Fn (E; see Fig. 1Avii&viii) and nuclei with DAPI. Scale bar 20 μm.

F. Representative individual channel IF images showing MIPs of fixed CRTL M2 cells or M2 cells treated for 30 min with T-β1, T-α, or T-β1/T-α and stained for p65, F-actin with phalloidin-TRITC, and nuclei with DAPI (i-iv). Merged images are presented in Fig. 4B. Scale bar =10 μm.

G-H. Representative individual channel IF images showing MIPs of M2 cells pre-treated with DMPM or vehicle control and stimulated with T-β1+T-α for 72 hrs. Cells were stained for N-Cad/Fn (Gi-iii) or Vim (H i-iii) and nucleus with DAPI. Merged images are presented in Fig. 5C. Scale bar 20 μm.

Figure S5

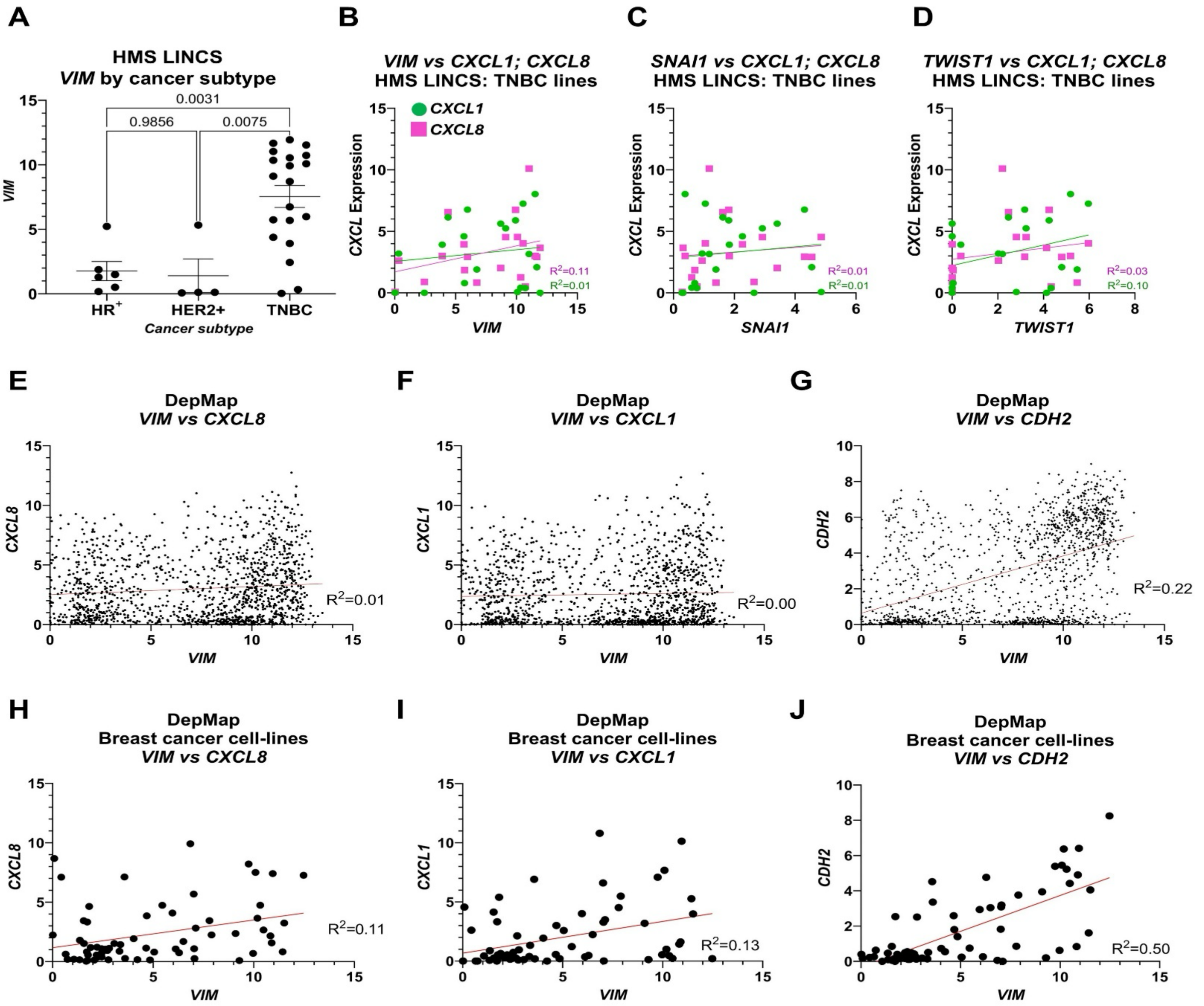
A
HMS LINCS
VIM by cancer subtype
0.0031
0.9856
0.0075
VIM
HR+
HER2+
TNBC
Cancer subtype
B
VIM vs CXCL1; CXCL8
HMS LINCS: TNBC lines
CXCL1
CXCL8
CXCL Expression
R2=0.11
R2=0.01
VIM
C
SNAI1 vs CXCL1; CXCL8
HMS LINCS: TNBC lines
CXCL Expression
R2=0.01
R2=0.01
SNAI1
D
TWIST1 vs CXCL1; CXCL8
HMS LINCS: TNBC lines
CXCL Expression
R2=0.03
R2=0.10
TWIST1
E
DepMap
VIM vs CXCL8
CXCL8
R2=0.01
VIM
F
DepMap
VIM vs CXCL1
CXCL1
R2=0.00
VIM
G
DepMap
VIM vs CDH2
CDH2
R2=0.22
VIM
H
DepMap
Breast cancer cell-lines
VIM vs CXCL8
CXCL8
R2=0.11
VIM
I
DepMap
Breast cancer cell-lines
VIM vs CXCL1
CXCL1
R2=0.13
VIM
J
DepMap
Breast cancer cell-lines
VIM vs CDH2
CDH2
R2=0.50
VIM

Figure S5. Analysis of gene expression datasets identifies no association between the expression of *CXCL8* or *CXCL1* and *VIM*

A. Expression level of *VIM* in HR+, HER2+, or TNBC cell lines available in the LINCs dataset.

B-D. Individual gene correlation between *CXCL8* or *CXCL1* against EMT markers *VIM* (B), *SNAI1* (C) and *TWIST1* (D) in 20 TNBC cell lines in the LINCs dataset.

E-G. Correlation between *VIM* and *CXCL8* (E), *CXCL1* (F) or *CDH2* (G) across all available cell lines in the DepMap dataset.

H-J. Correlation between *VIM* and *CXCL8* (H), *CXCL1* (I) or *CDH2* (J) across breast cancer cell lines in the DepMap dataset.

Table S1.

Treatment condition	T-β1 in CM (pg/ml)
CTRL	0
T-β1+T-α	0
CTRL	0
T-β1+T-α	0
CTRL	4
T-β1+T-α	5

Table S1

TGF-β1/TNF-α treatment does not induce TGF-β1 secretion from M2 cells

Table showing the amount (pg/ml) of TGF-β1 secreted by CTRL or T-β1/T-α treated M2 cells from three independent experiments.

Fig 1 Ci

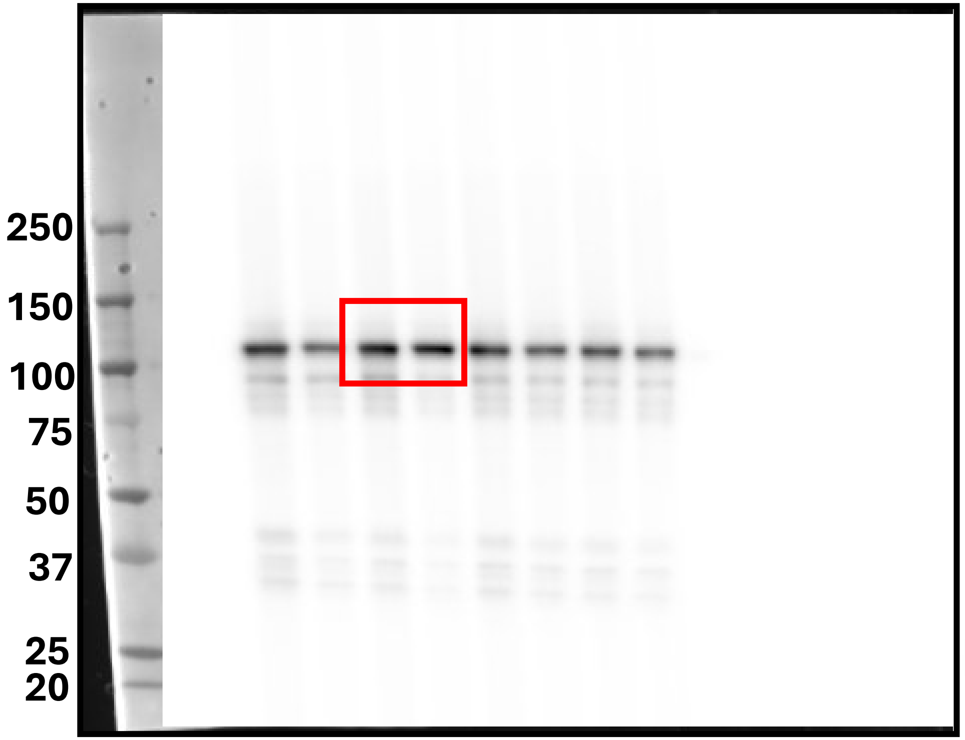

E-Cad (120 kDa)

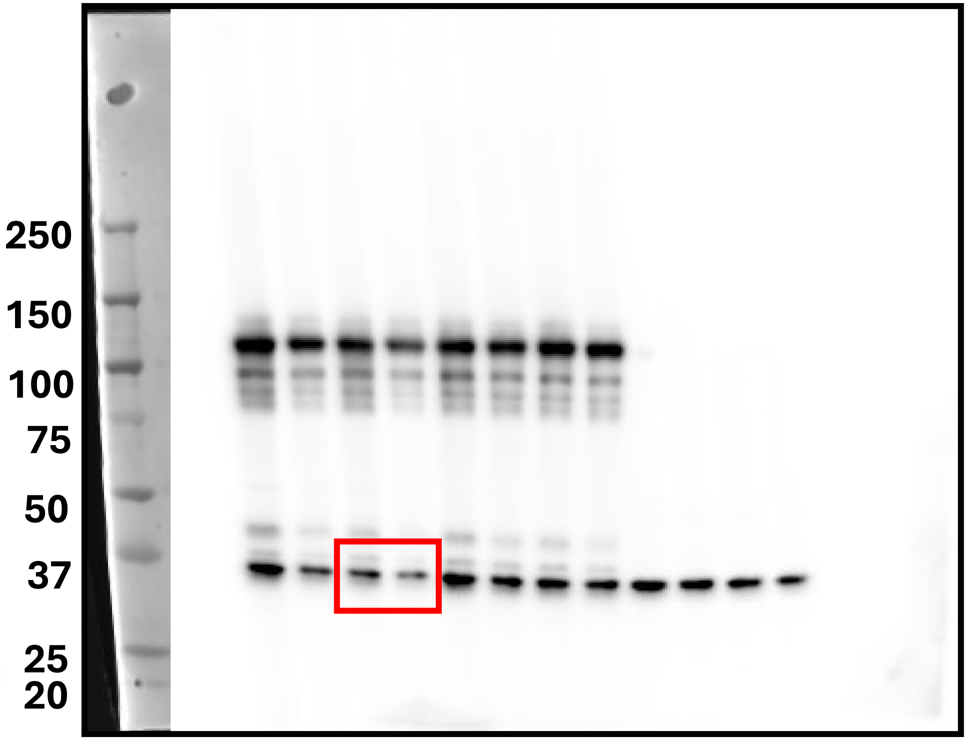

GAPDH (36 kDa)

Fig 1 Cii

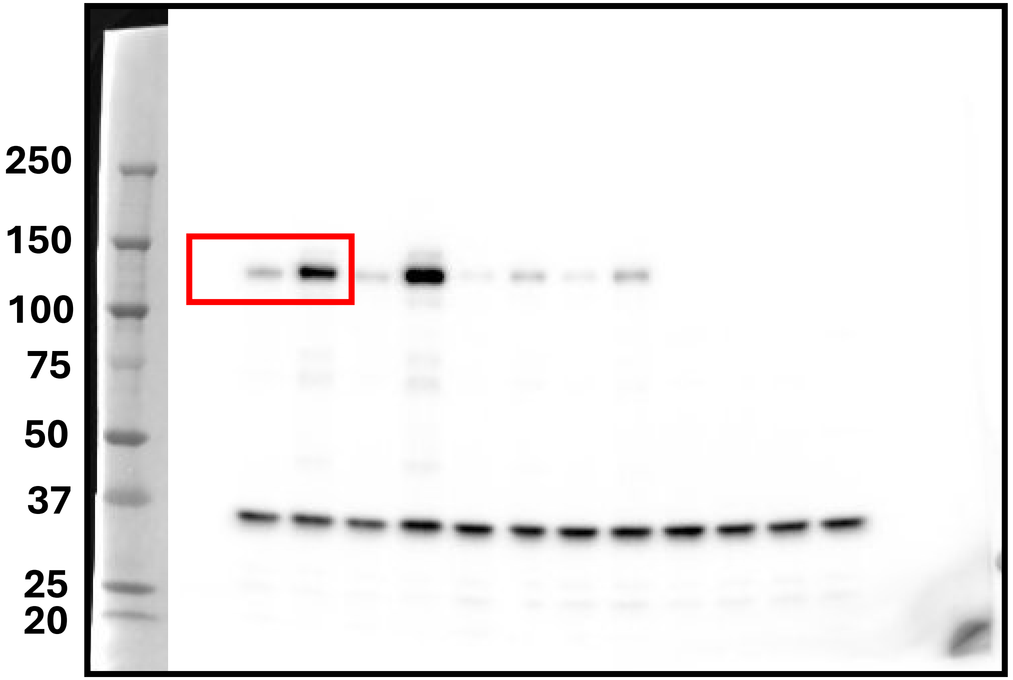

N-Cad (140 kDa)

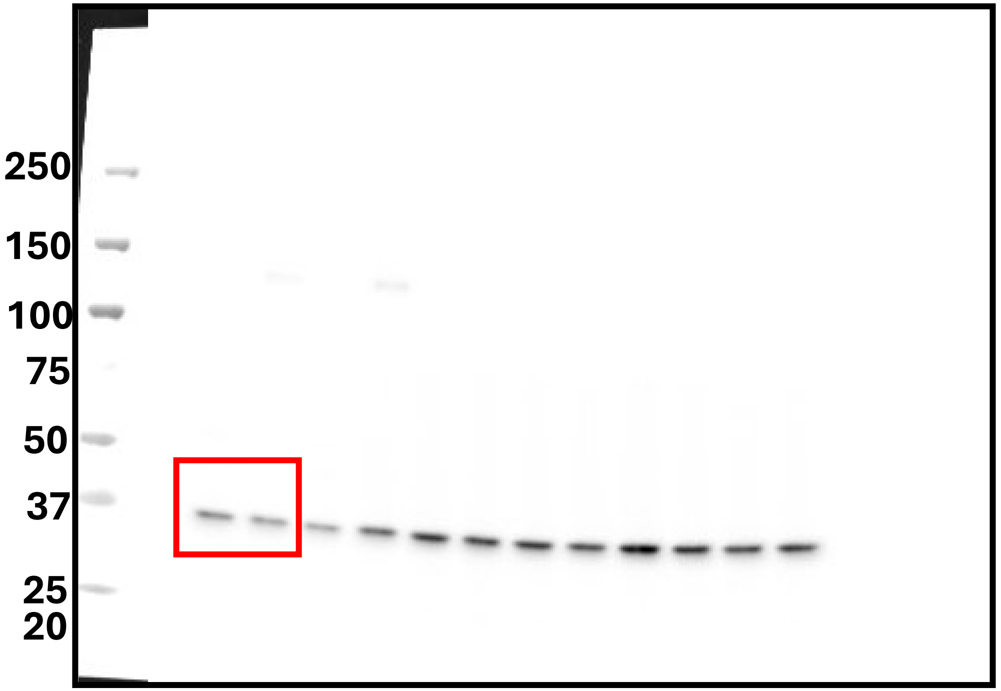

GAPDH (36 kDa)

Fig 1 Ciii

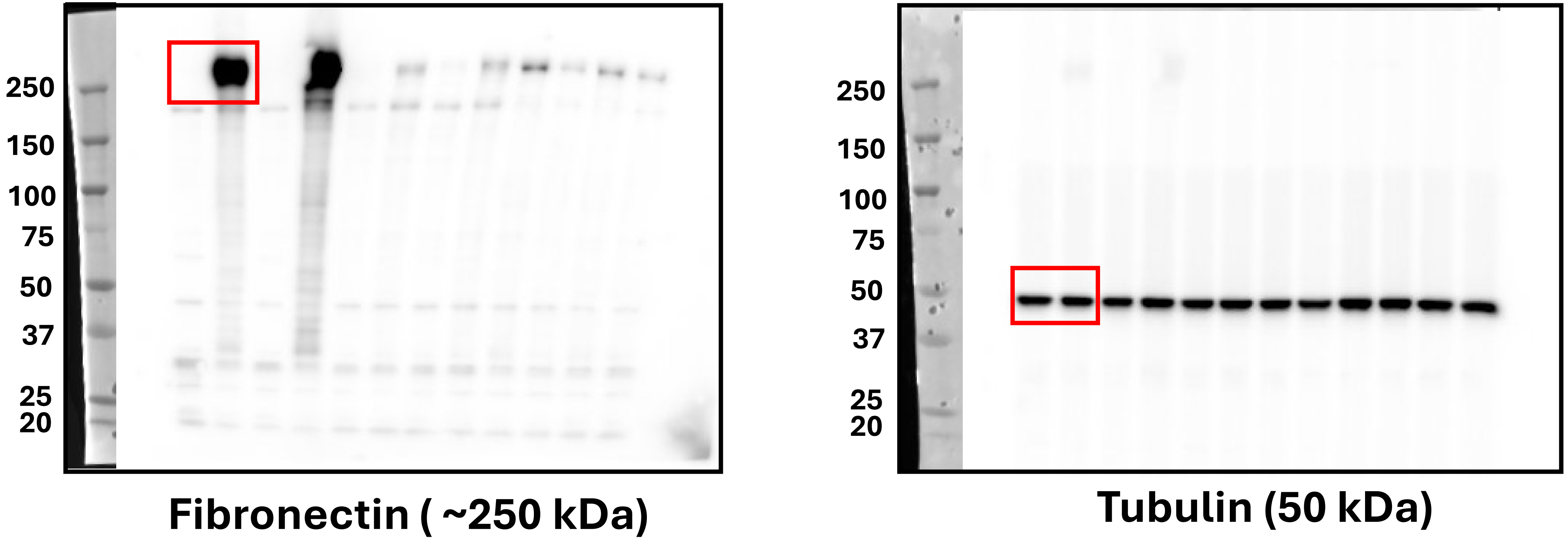
250
150
100
75
50
37
25
20
Fibronectin (~250 kDa)
250
150
100
75
50
37
25
20
Tubulin (50 kDa)

Fig 2 Ai

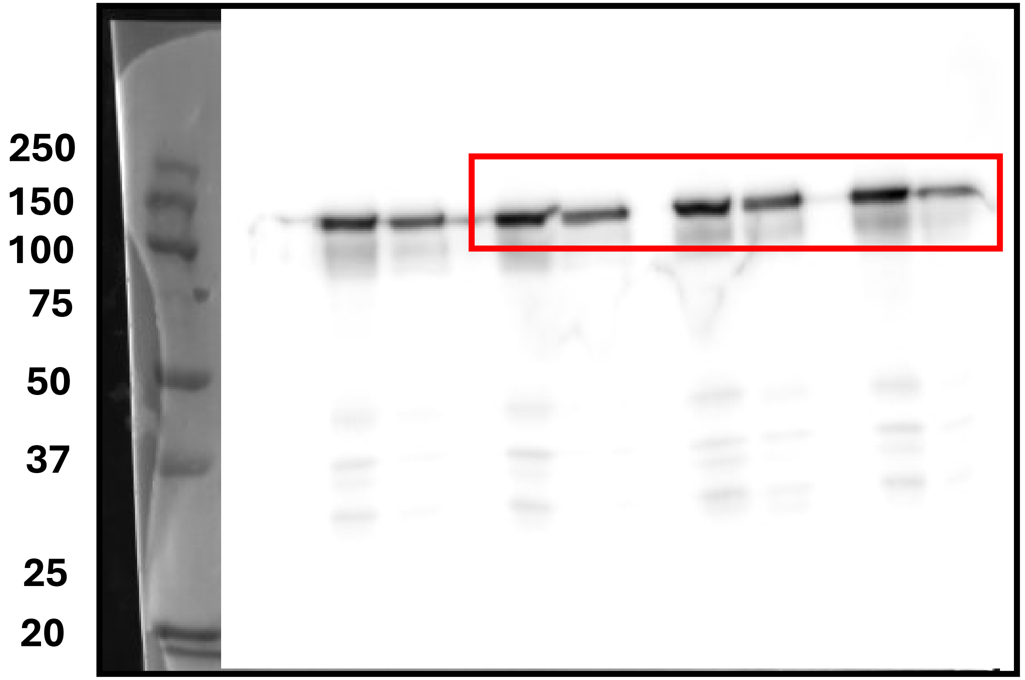

E-Cad (120 kDa)

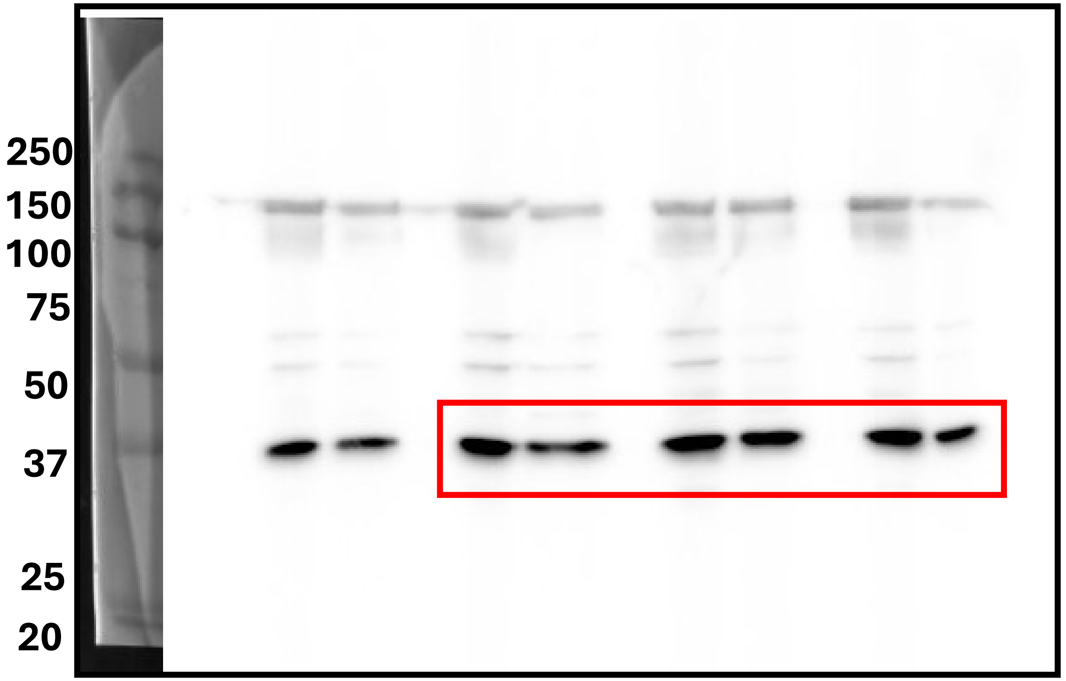

GAPDH (36 kDa)

Fig 2 Aii

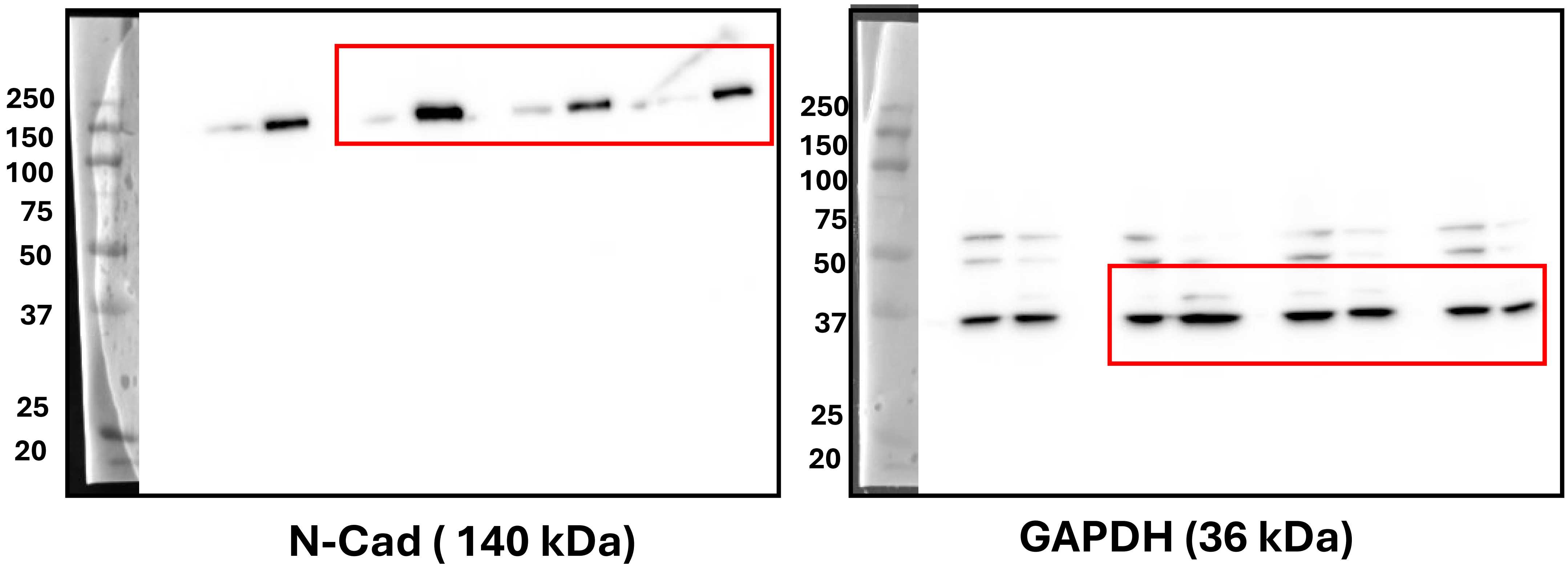
250
150
100
75
50
37
25
20
N-Cad (140 kDa)
250
150
100
75
50
37
25
20
GAPDH (36 kDa)

Fig 2 Aiii

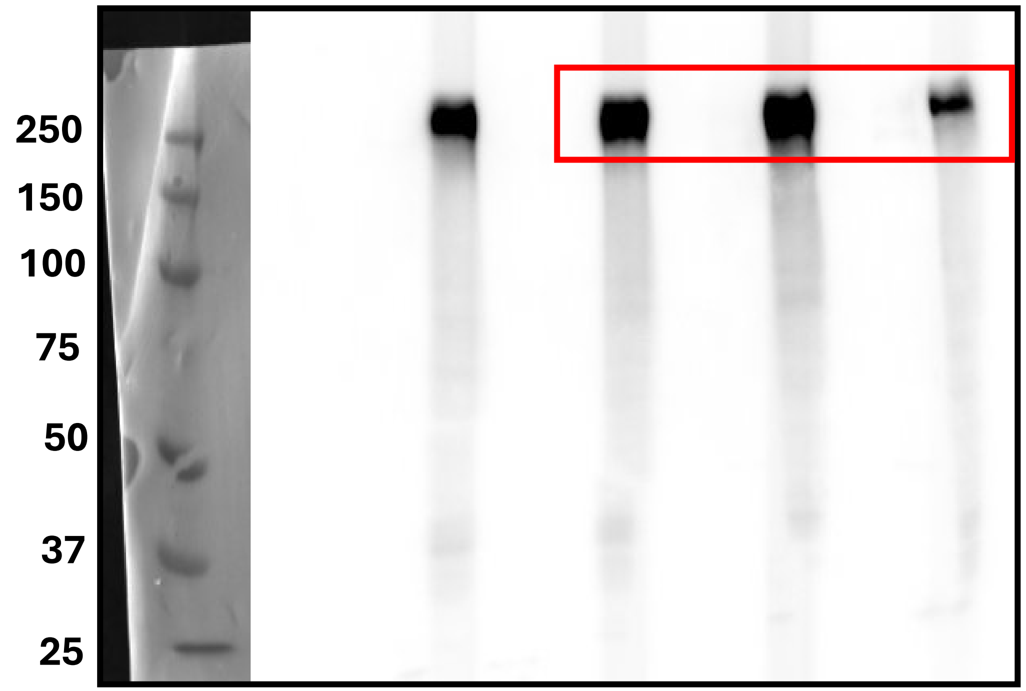

Fibronectin (~250 kDa)

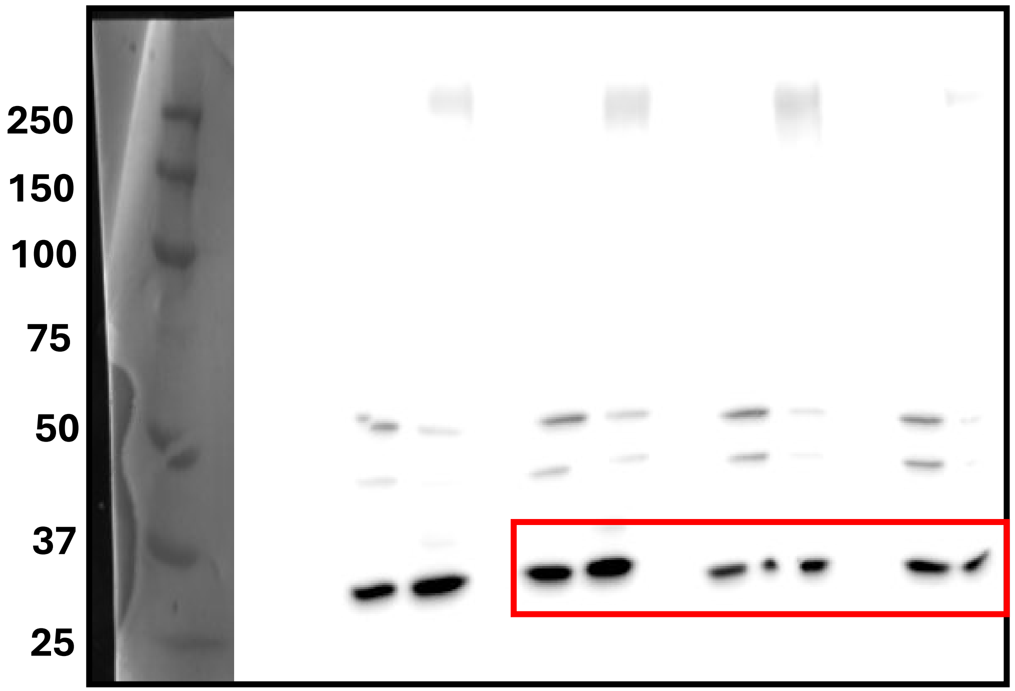

GAPDH (36 kDa)

Fig 2 D

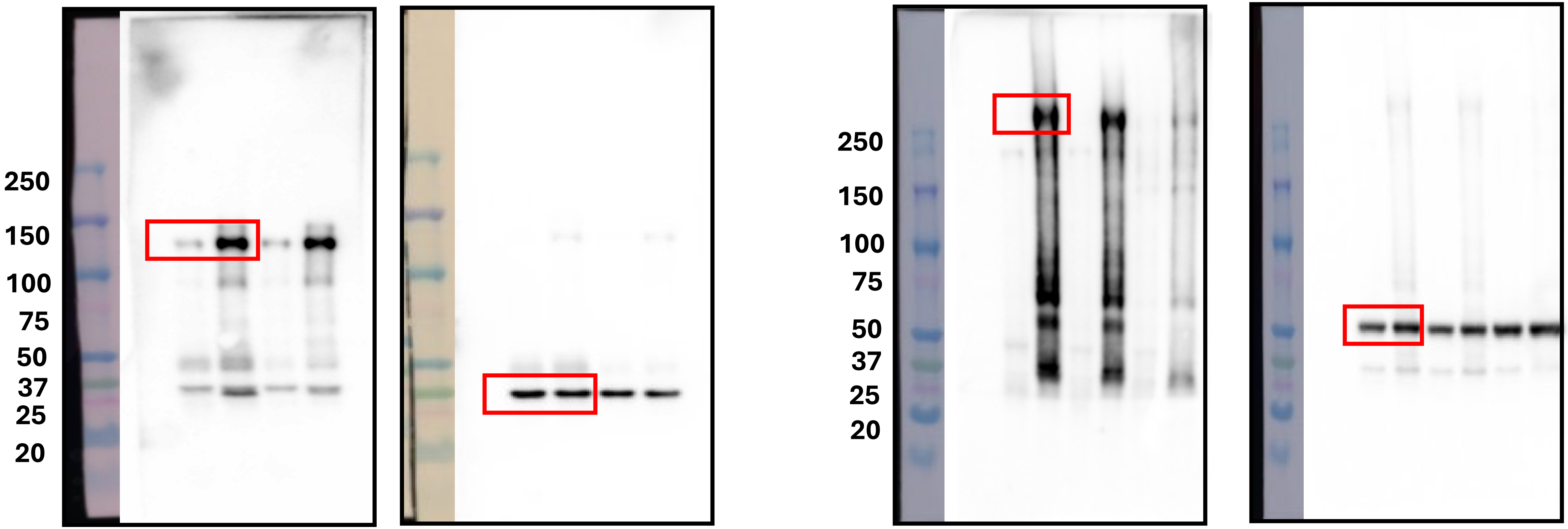

N-Cad (140 kDa) GAPDH (36 kDa) Fibronectin (~250 kDa) Tubulin (50 kDa)

Fig 3 Bi

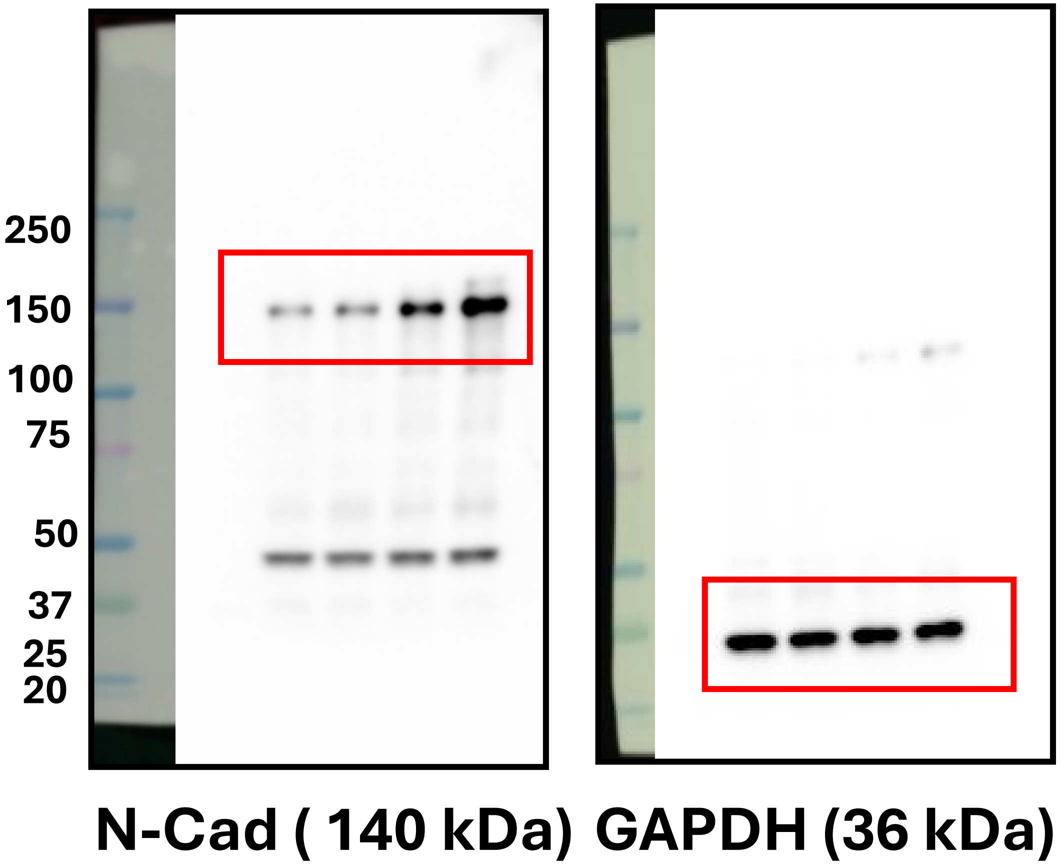

Fig 3 Bii

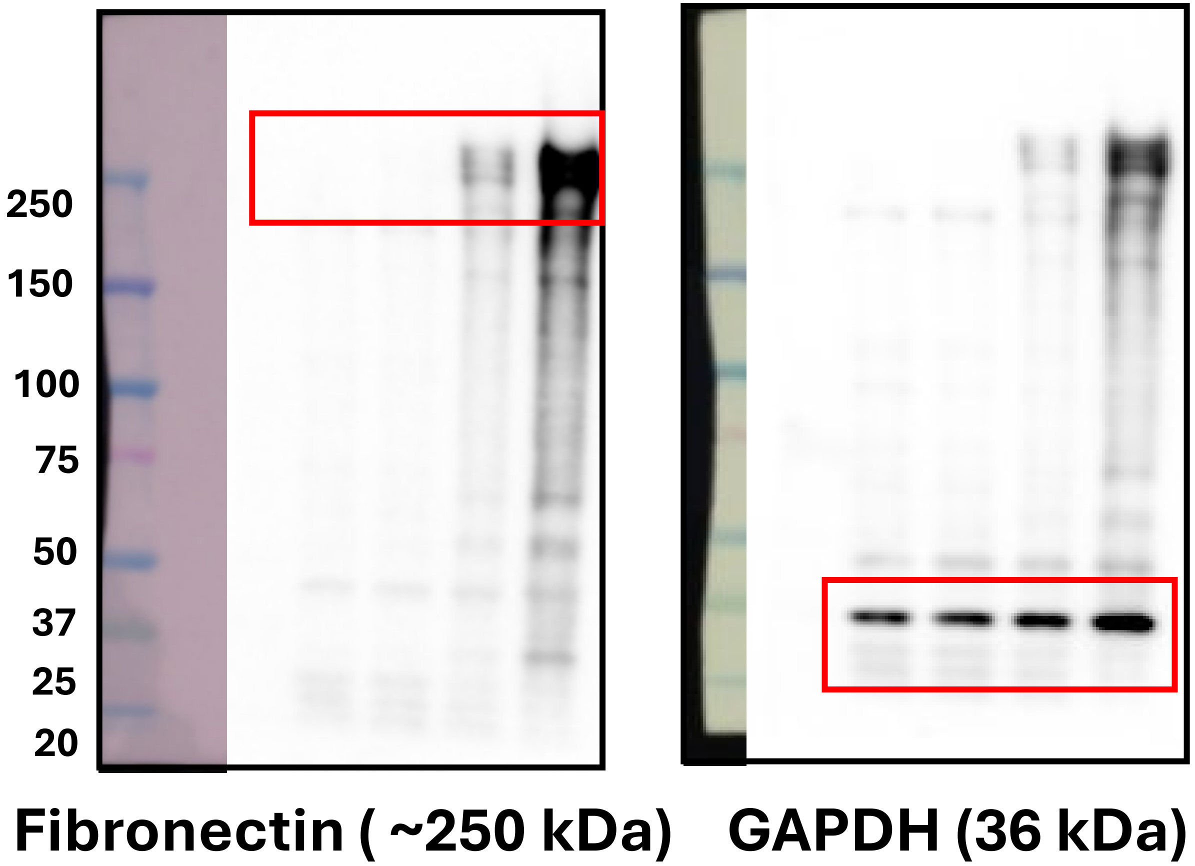

Fig 4 A

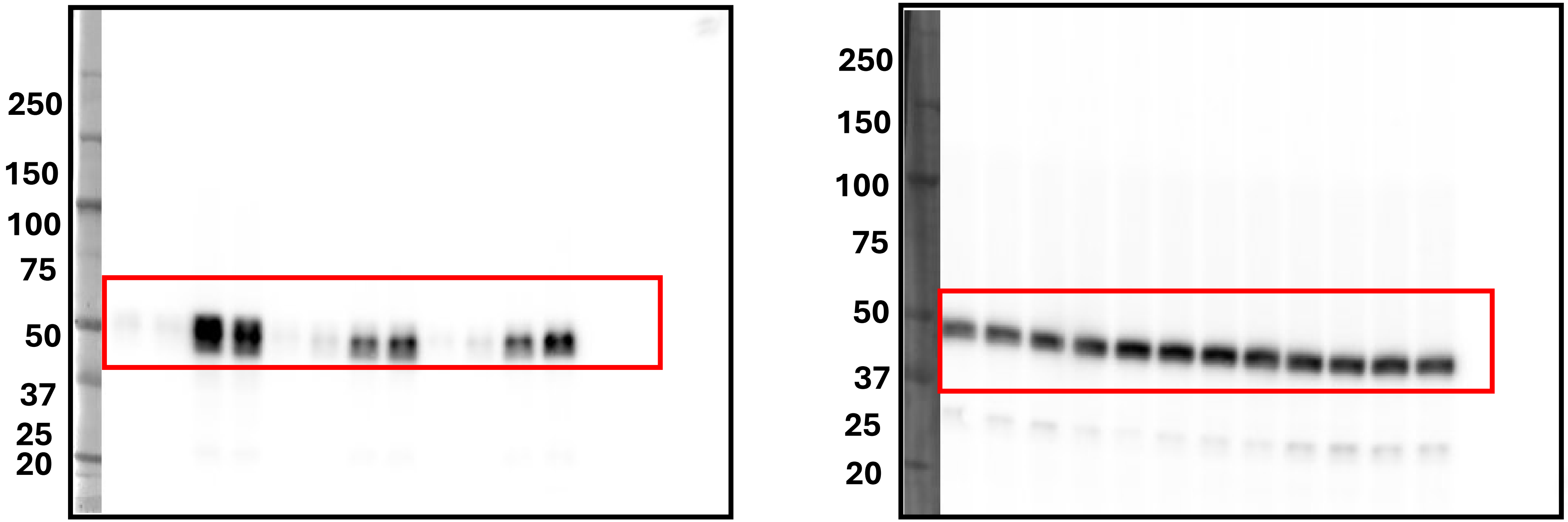

P-SMAD3 (52 kDa)

Tubulin (50 kDa)

Fig 4 Ci

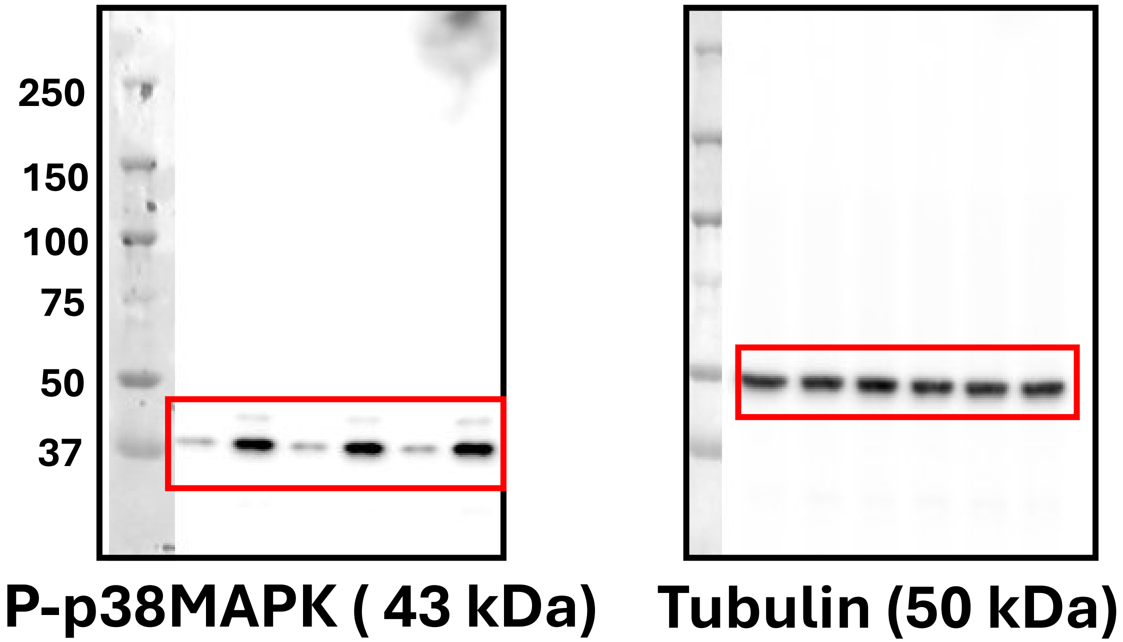

Fig 4 Cii

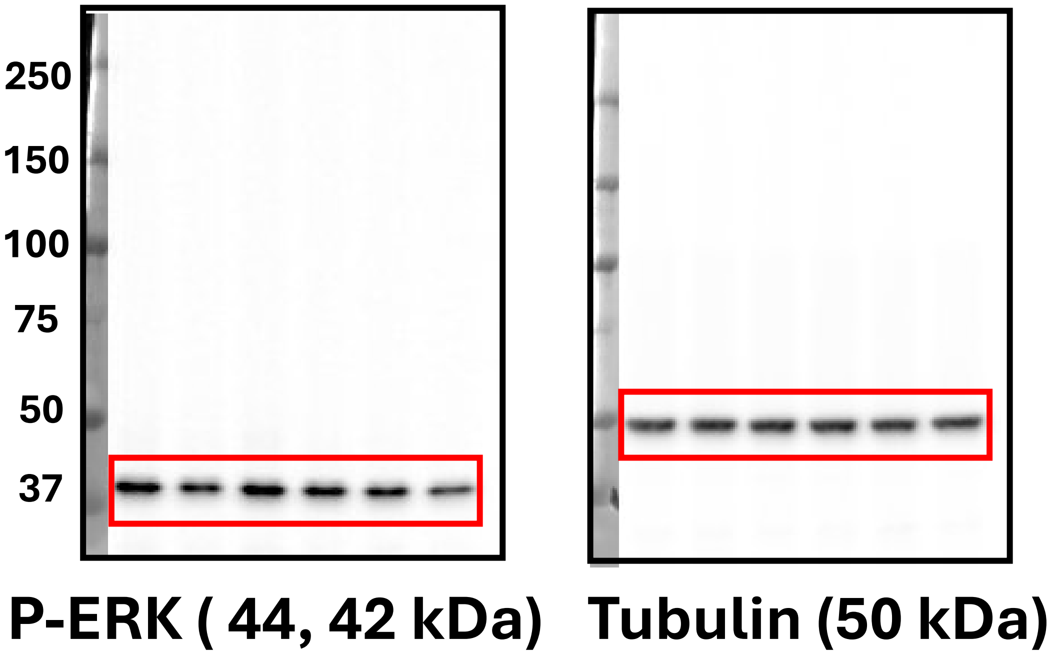

Fig 4 Ciii

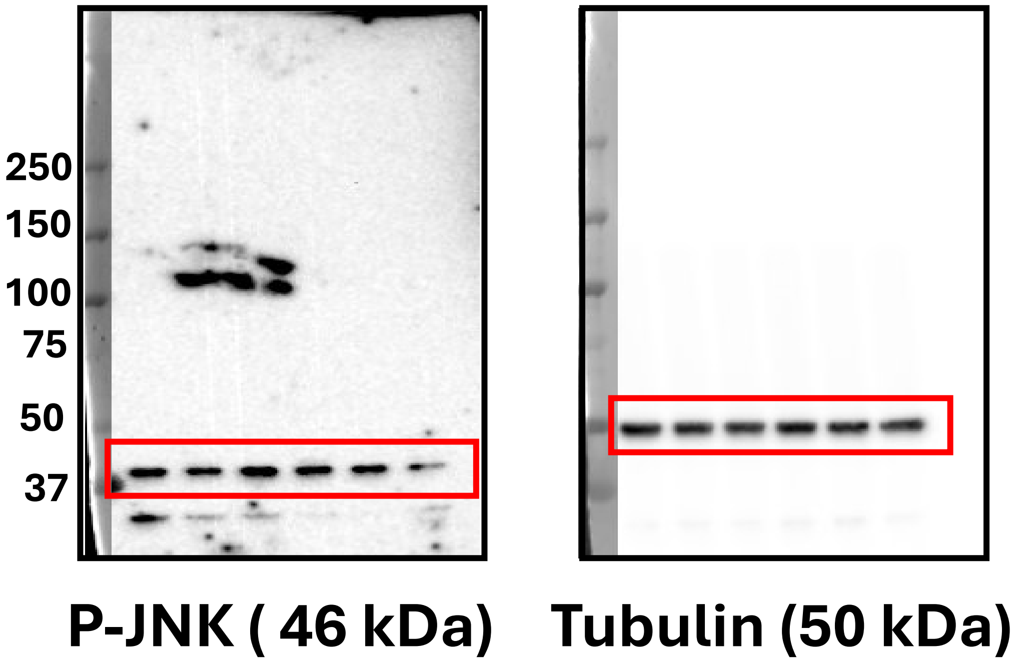

Fig 4 Civ

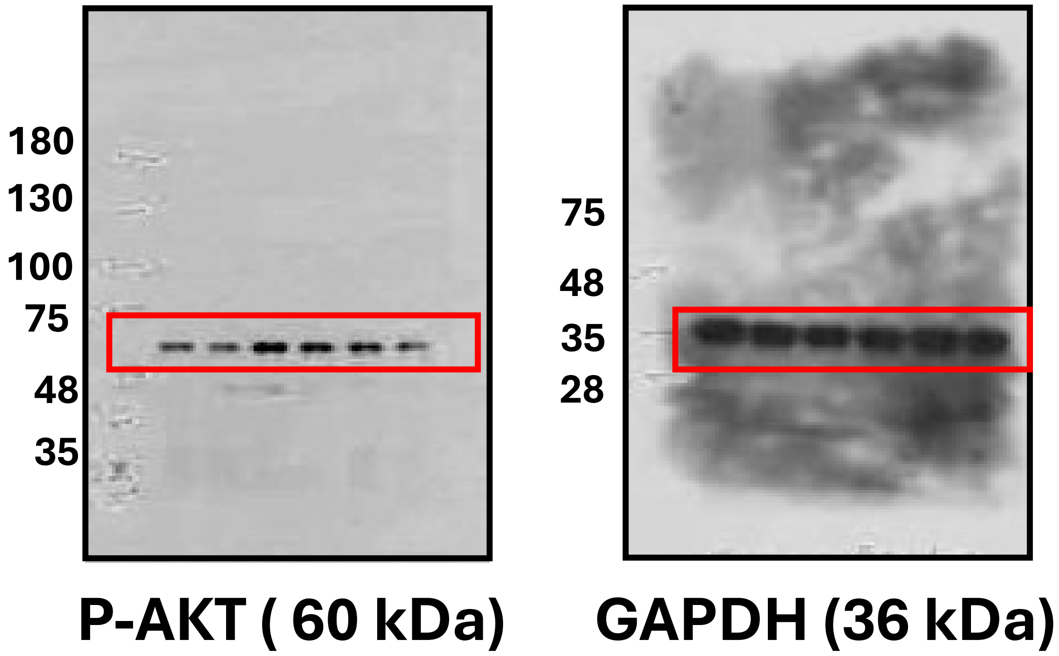

Fig 5A

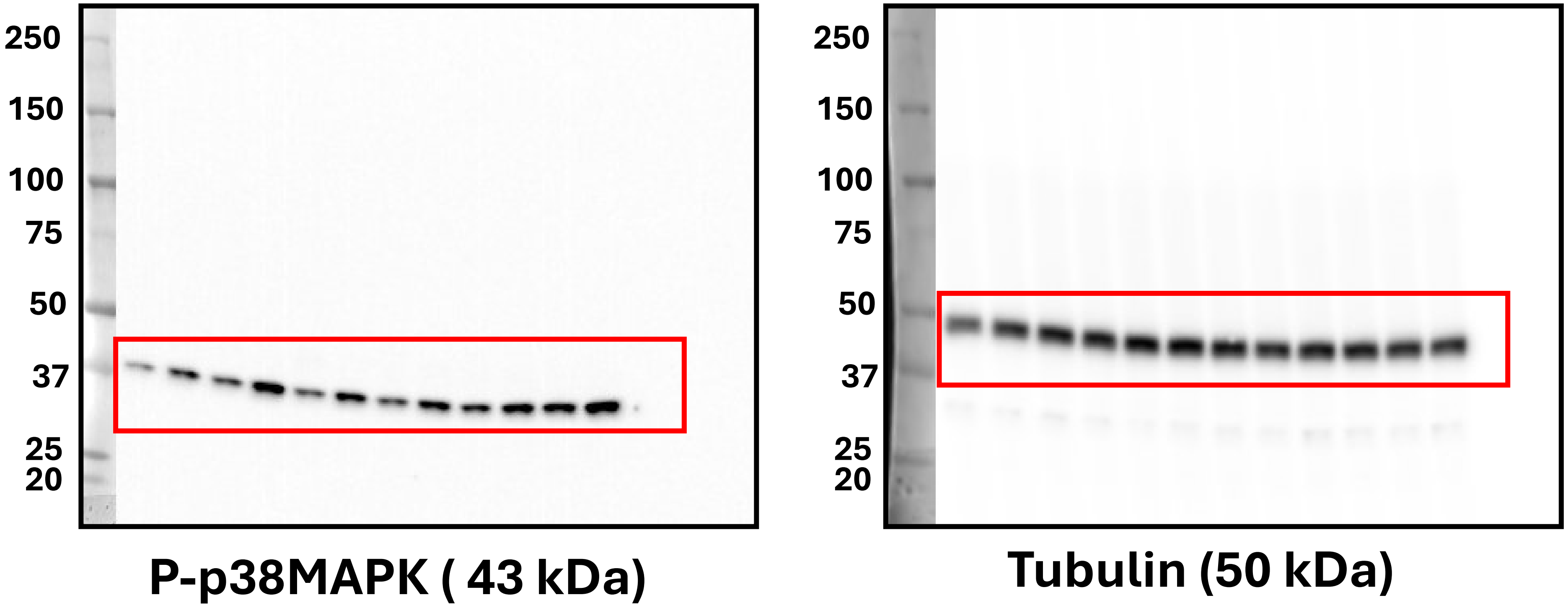
250
150
100
75
50
37
25
20
P-p38MAPK (43 kDa)
250
150
100
75
50
37
25
20
Tubulin (50 kDa)

Fig 5B

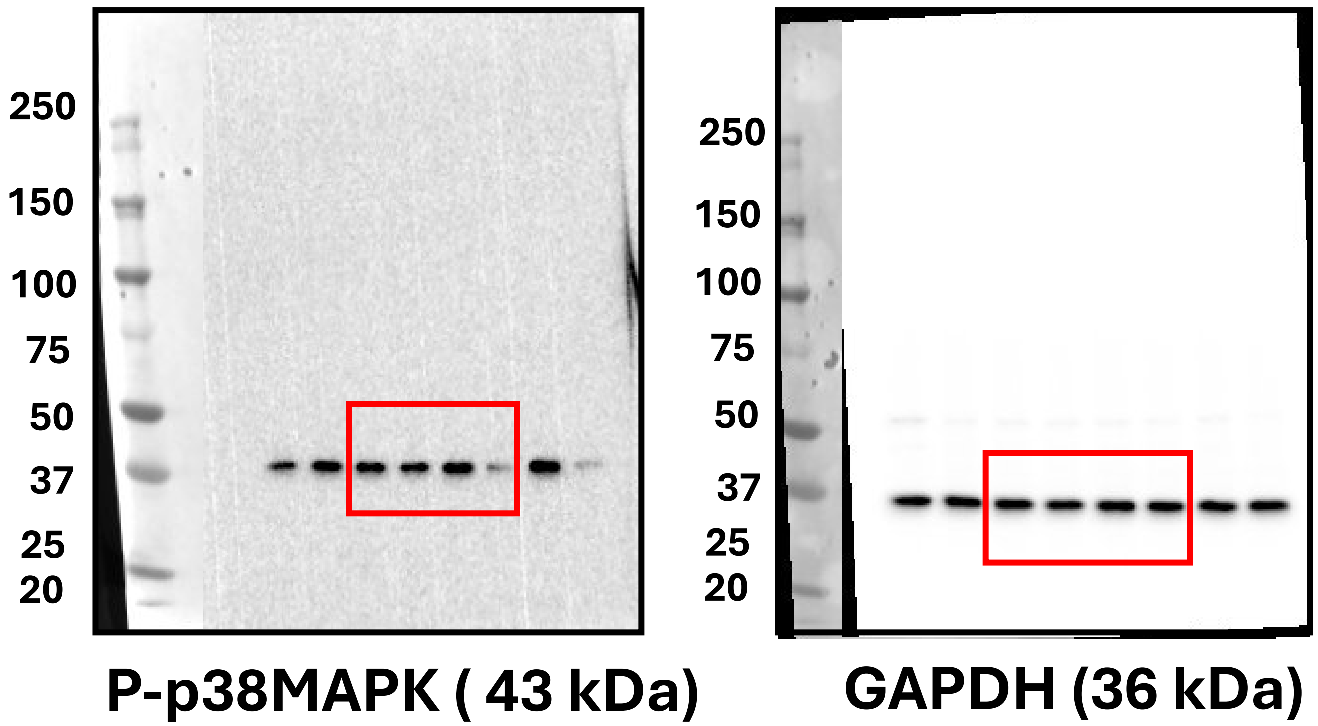
250
150
100
75
50
37
25
20
P-p38MAPK (43 kDa)
250
150
100
75
50
37
25
20
GAPDH (36 kDa)

Fig S1 A

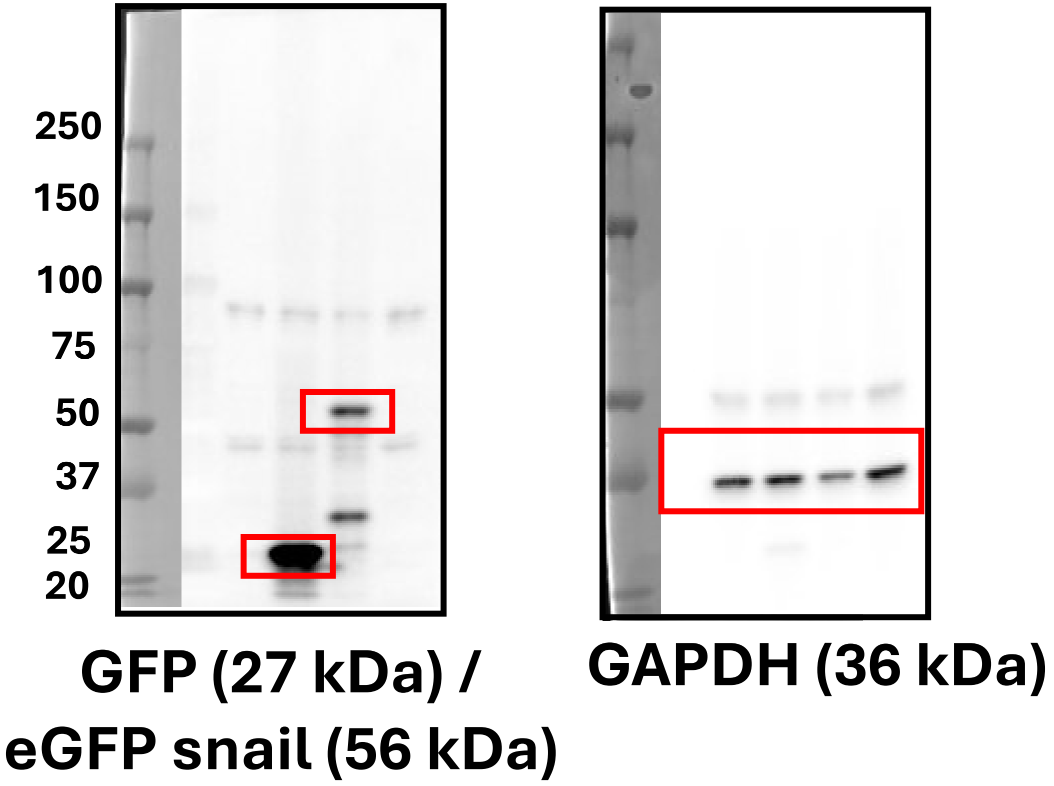

Fig S1 B

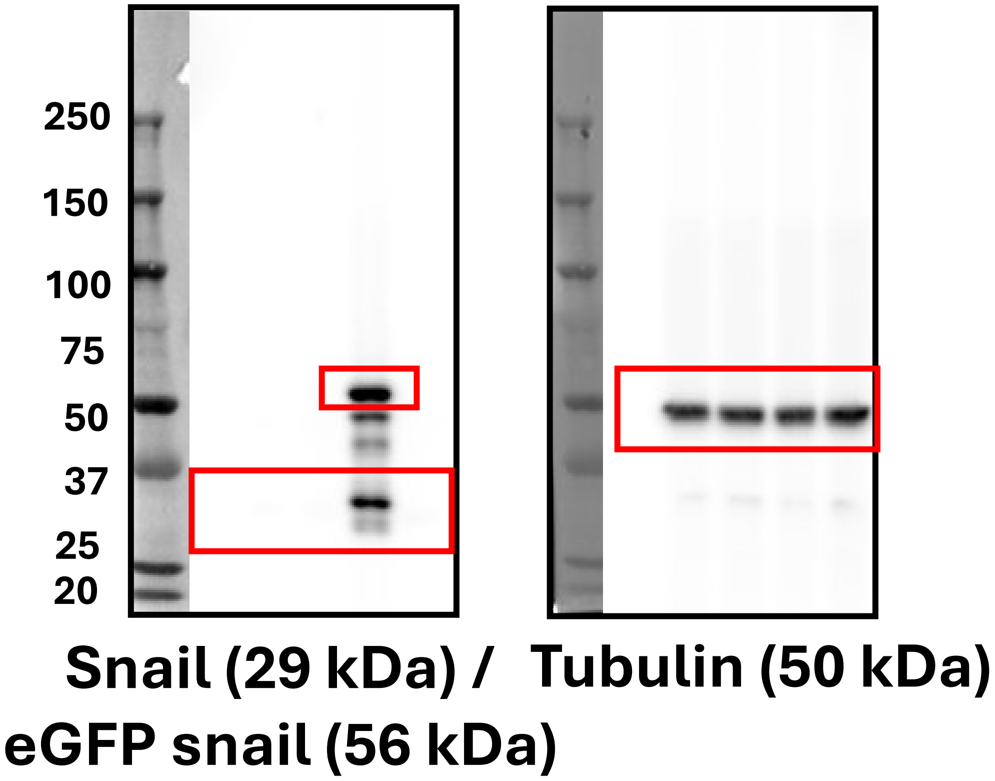

Fig S1 C

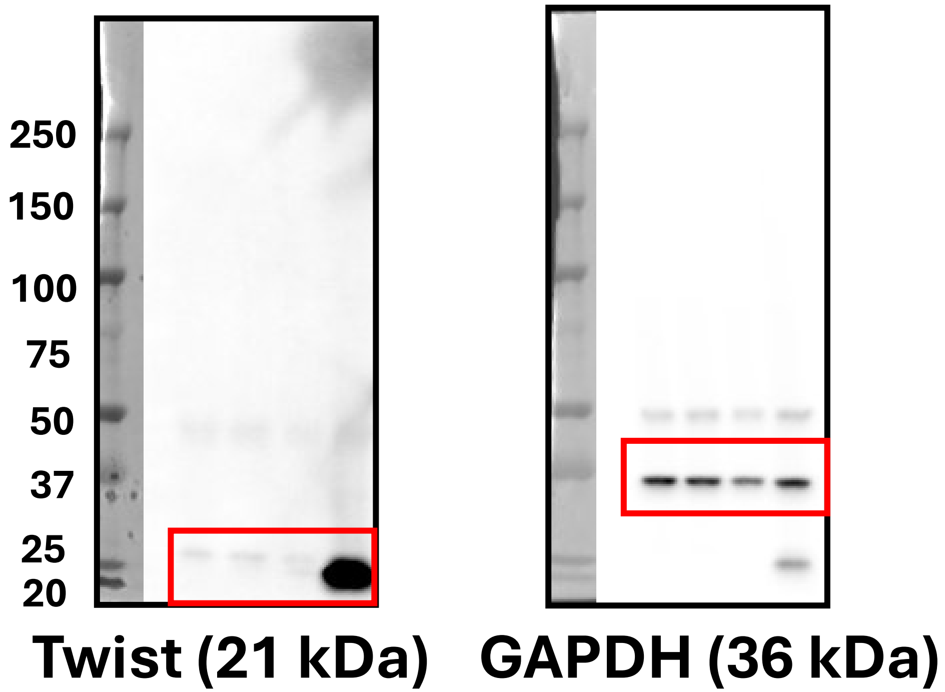

Fig S1 E

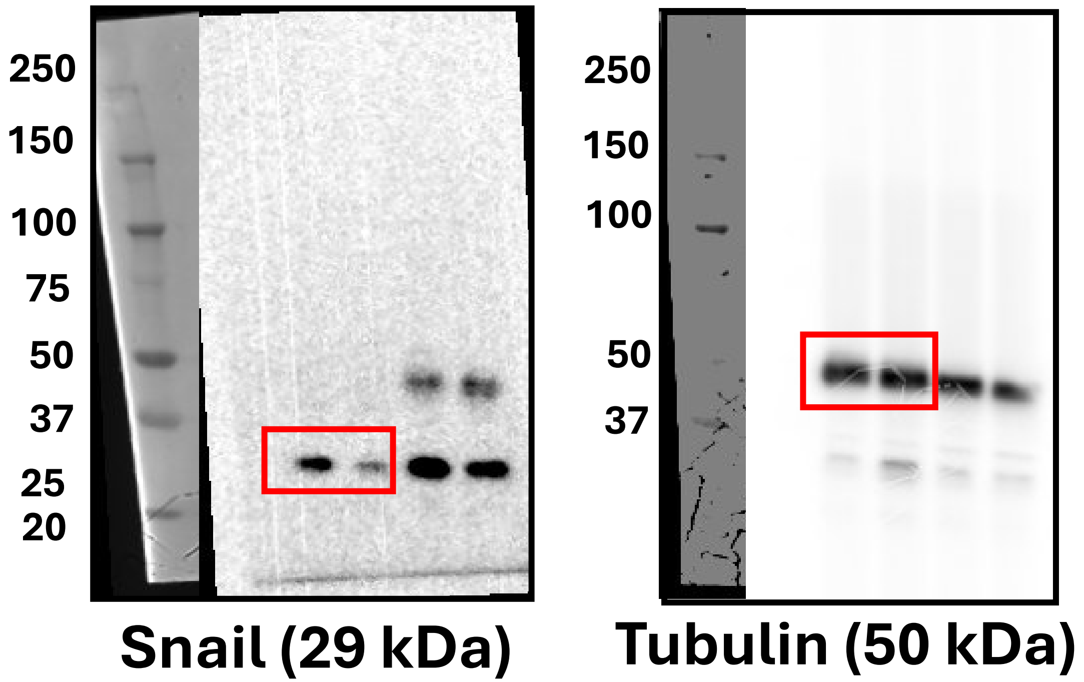

Fig S1 Fi

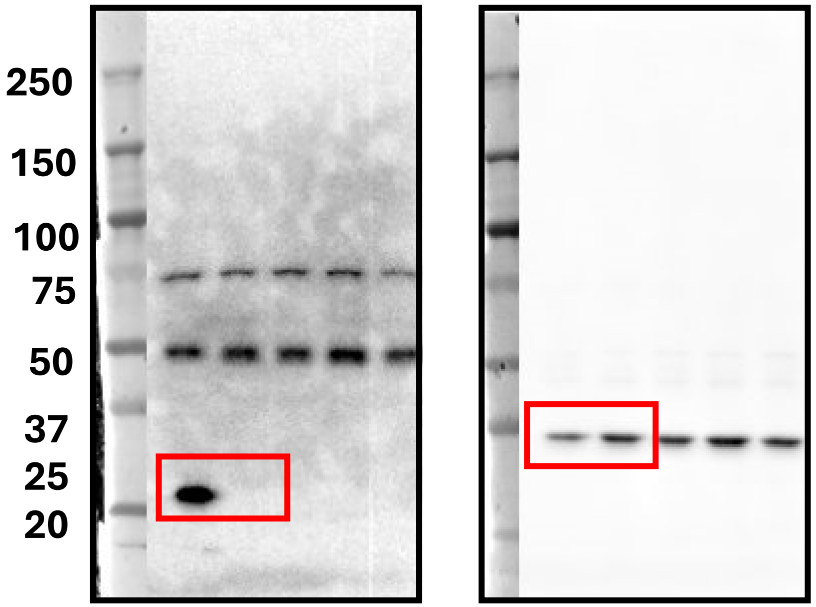

Twist (21 kDa) GAPDH (36 kDa)

Fig S1 Fii, iii

Fig S1 Fii, iii

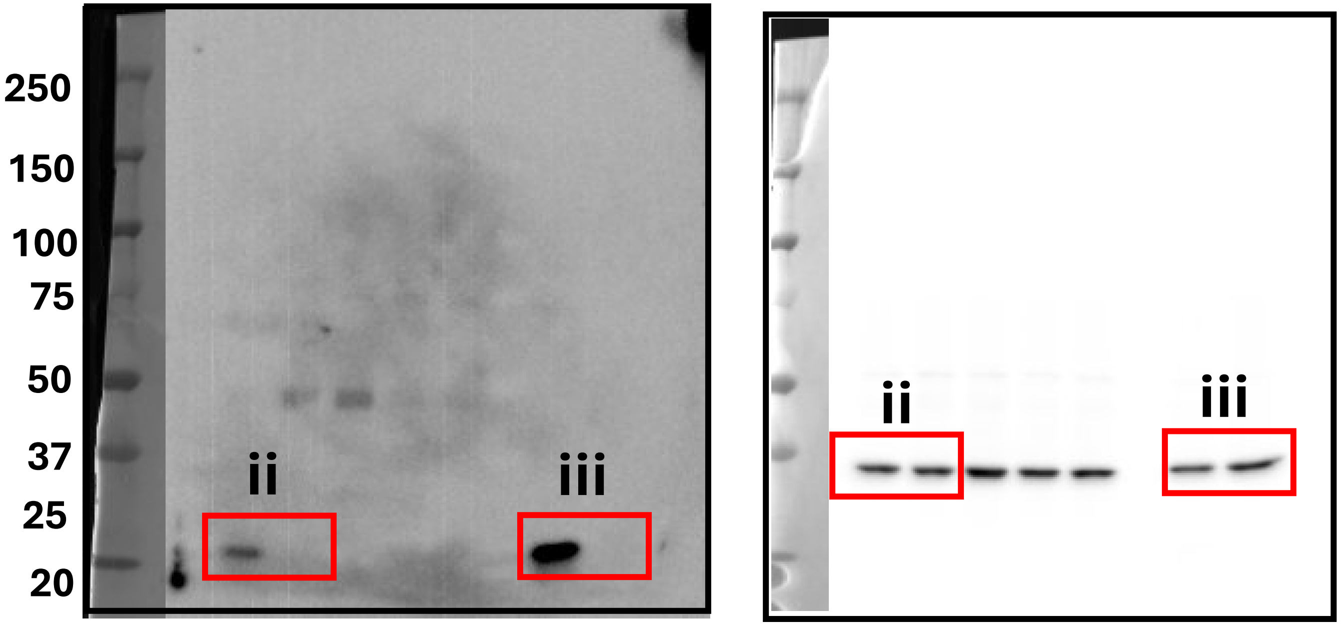

Twist (21 kDa) GAPDH (36 kDa)

Fig S2 Ai

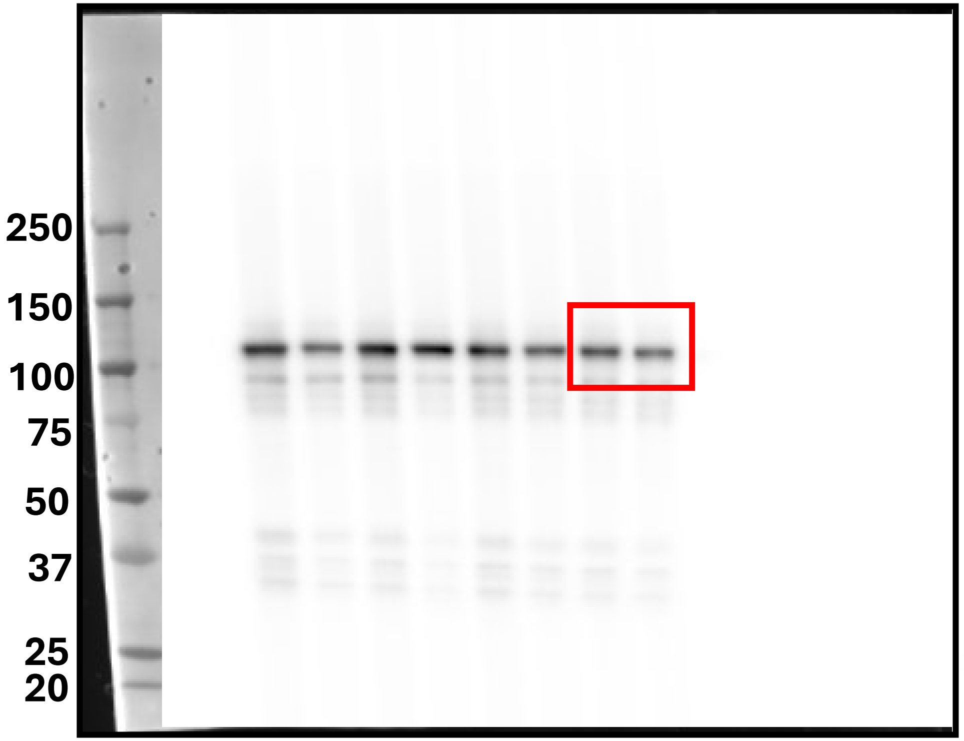

E-Cad (120 kDa)

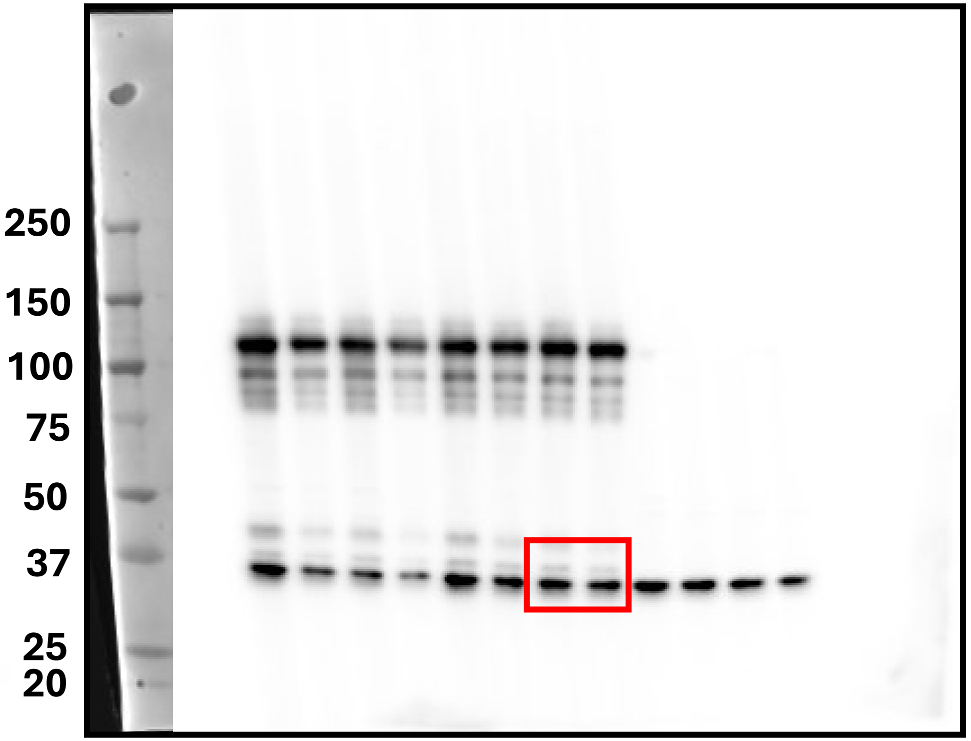

GAPDH (36 kDa)

Fig S2 Aii

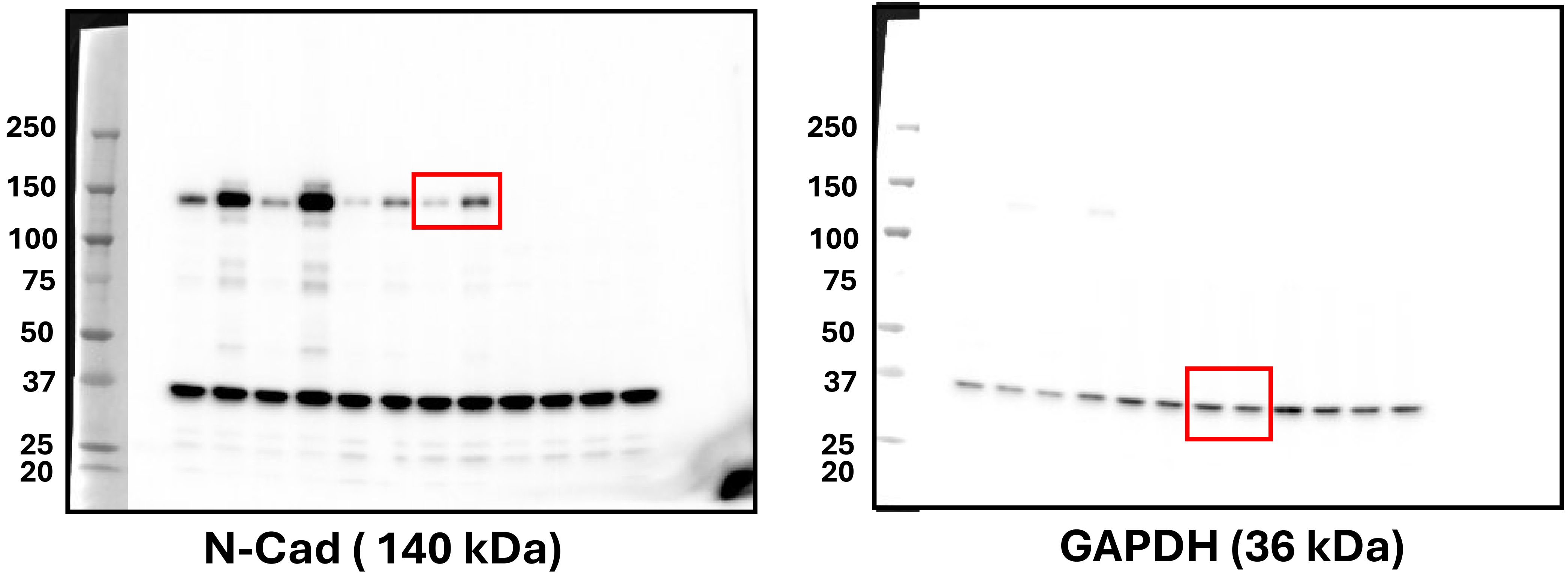
250
150
100
75
50
37
25
20
N-Cad (140 kDa)
250
150
100
75
50
37
25
20
GAPDH (36 kDa)

Fig S2 Aiii

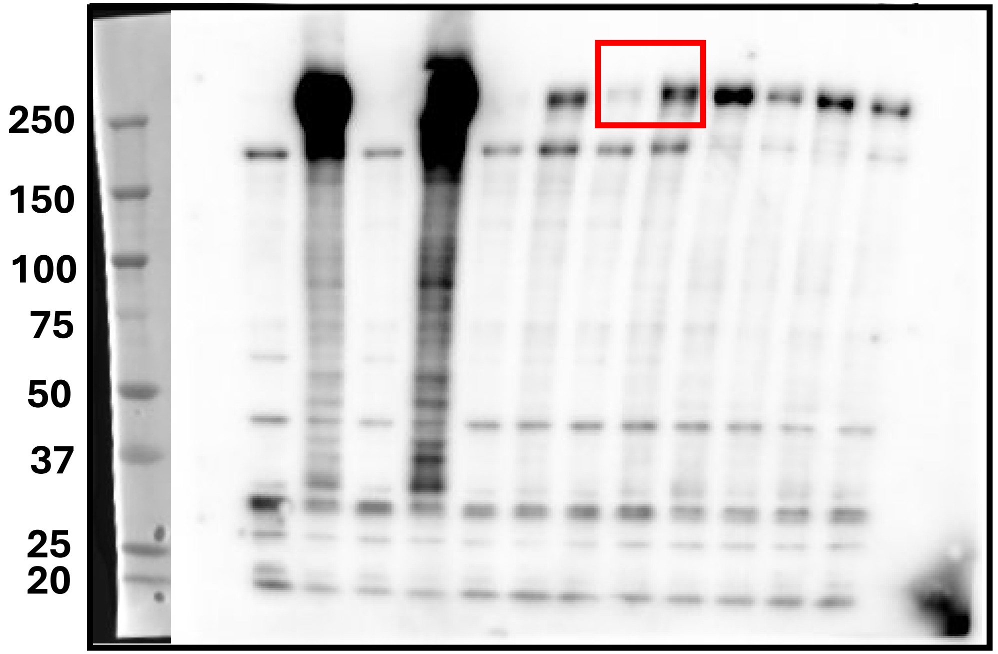

Fibronectin (~250 kDa)

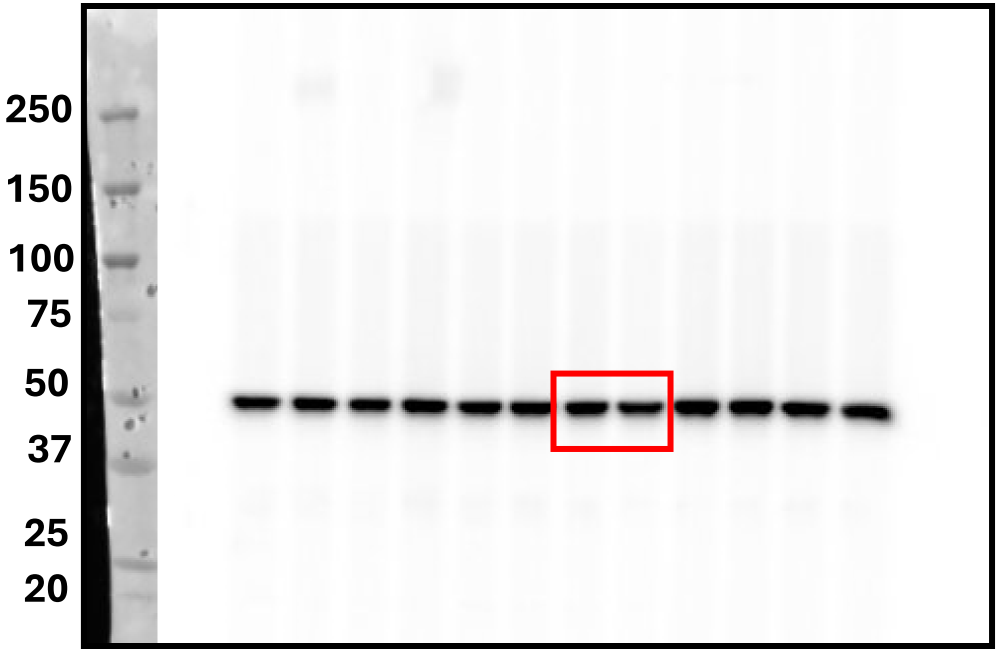

Tubulin (50 kDa)

Fig S2 B

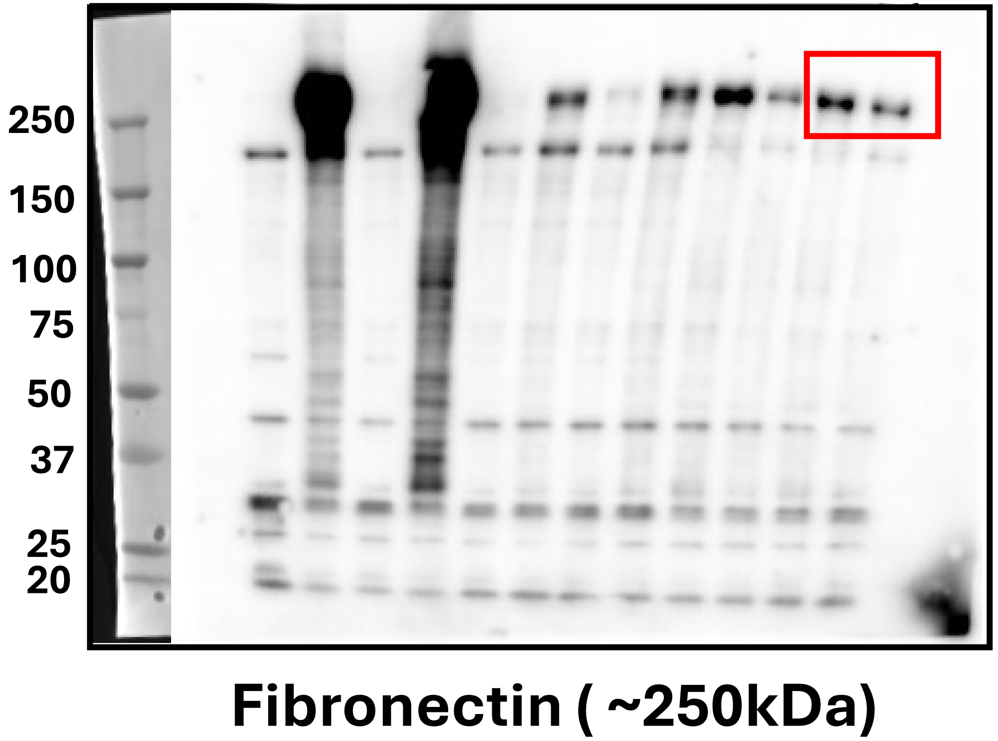
250
150
100
75
50
37
25
20
Fibronectin (~250kDa)

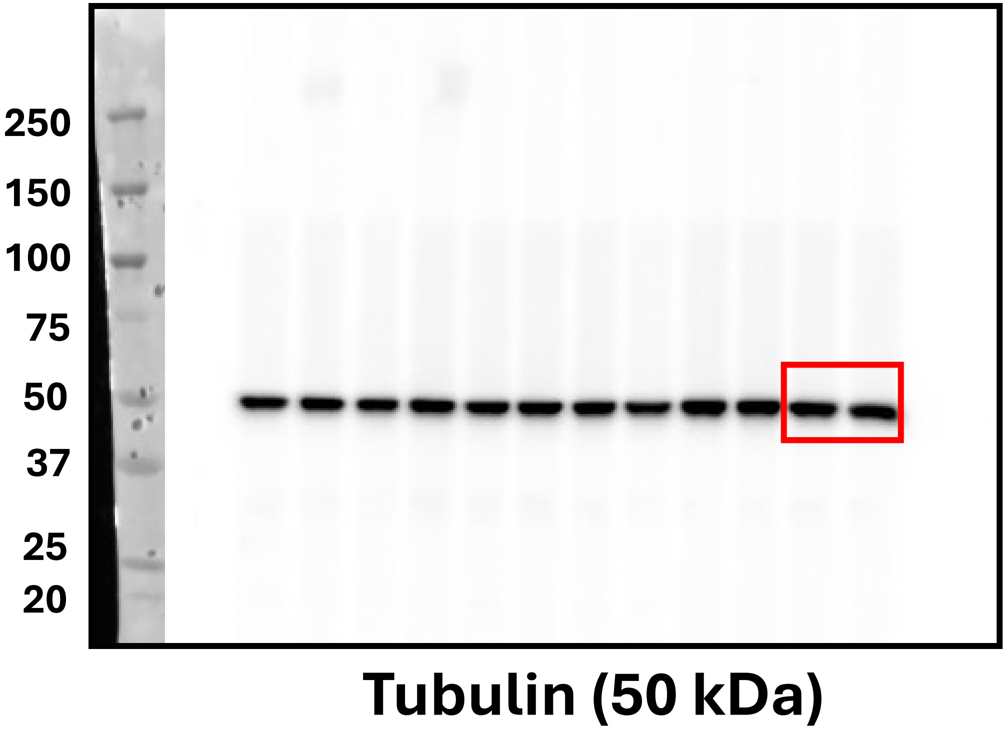
250
150
100
75
50
37
25
20
Tubulin (50 kDa)

Fig S2 C

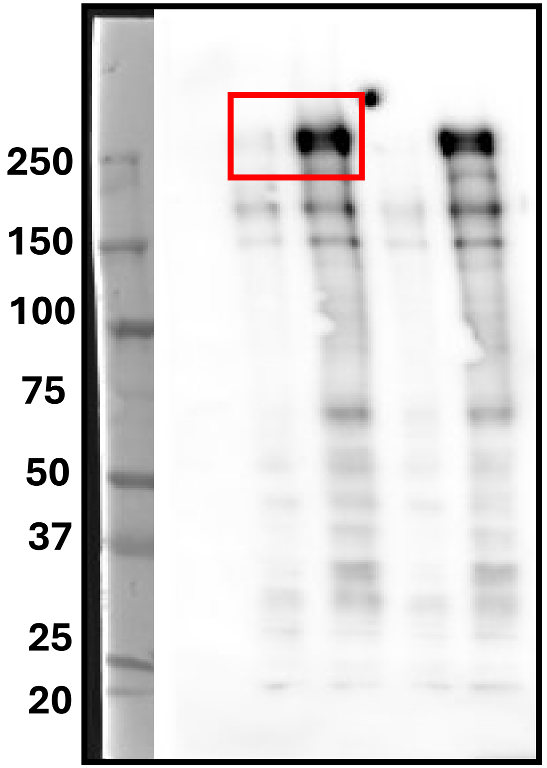

Fibronectin (~250 kDa)

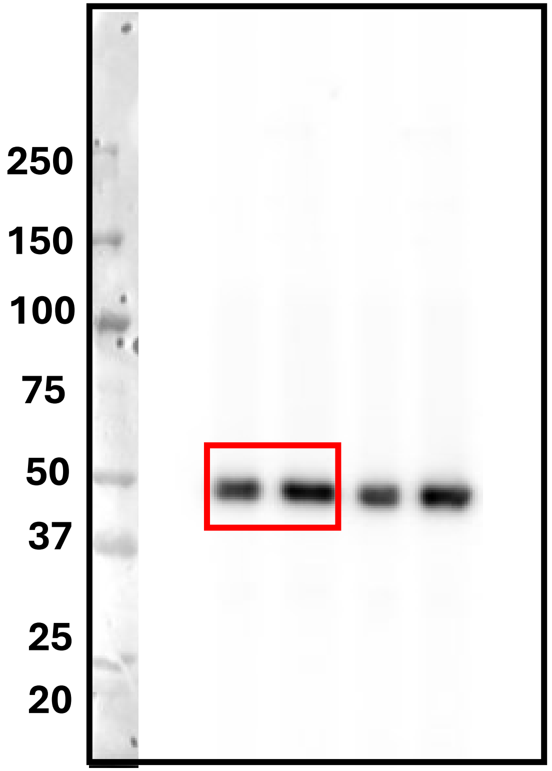

Tubulin (50 kDa)

Fig S3 A

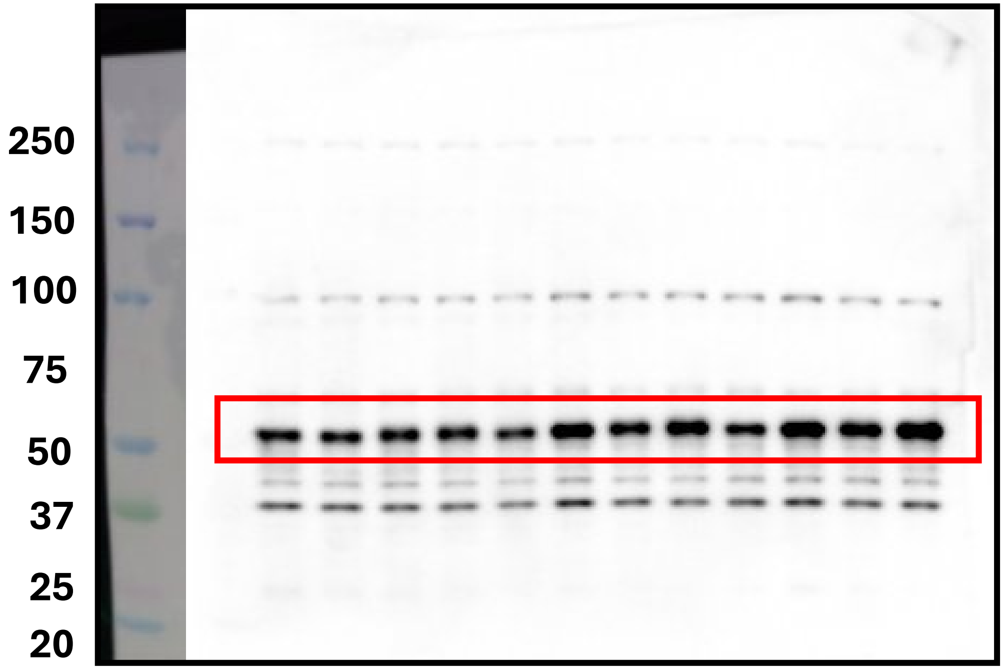

Smad3 (52 kDa)

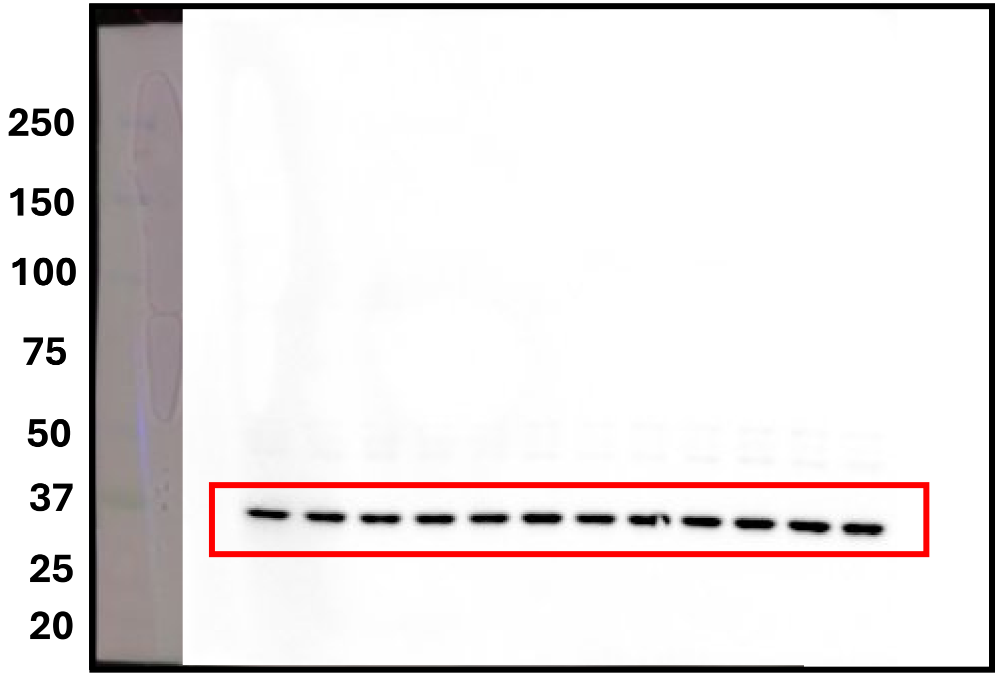

GAPDH (36 kDa)

Fig S3 B

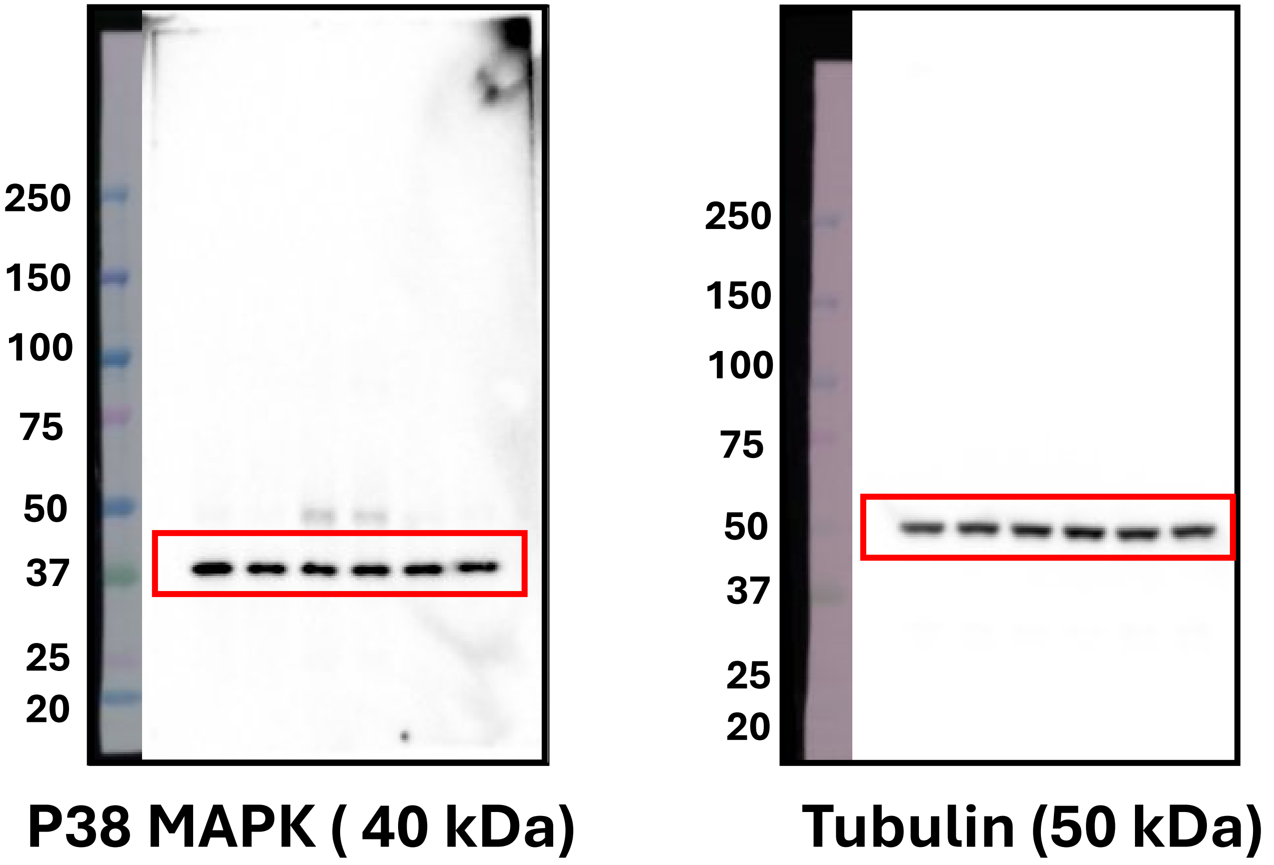
250
150
100
75
50
37
25
20
P38 MAPK (40 kDa)
250
150
100
75
50
37
25
20
Tubulin (50 kDa)

Fig S3 C

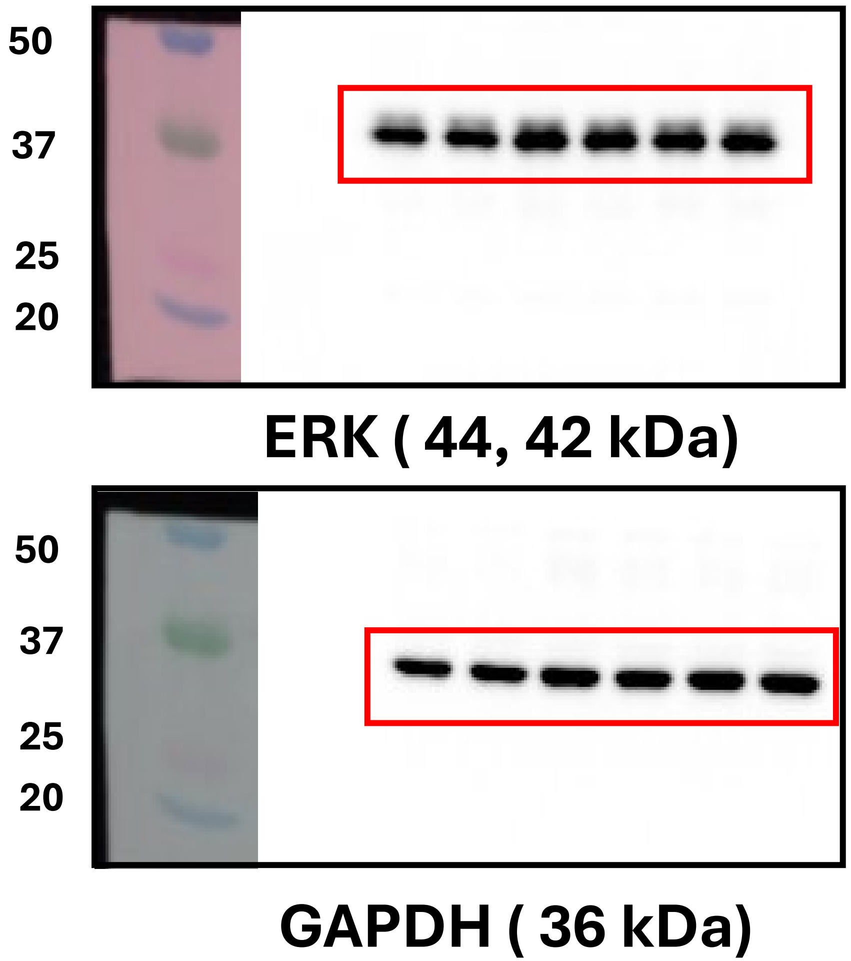

Fig S3 D

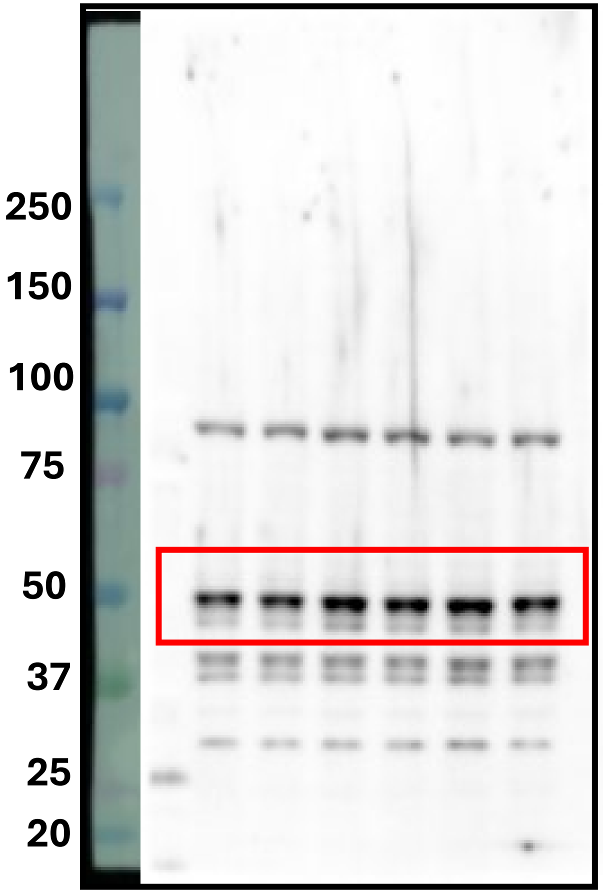

JNK (46 kDa)

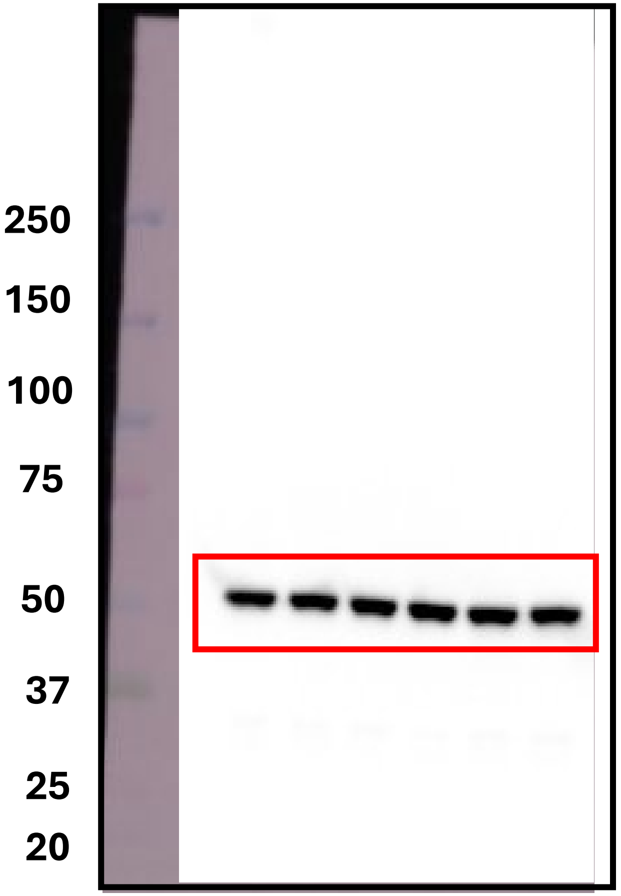

Tubulin (50 kDa)

Fig S3 E

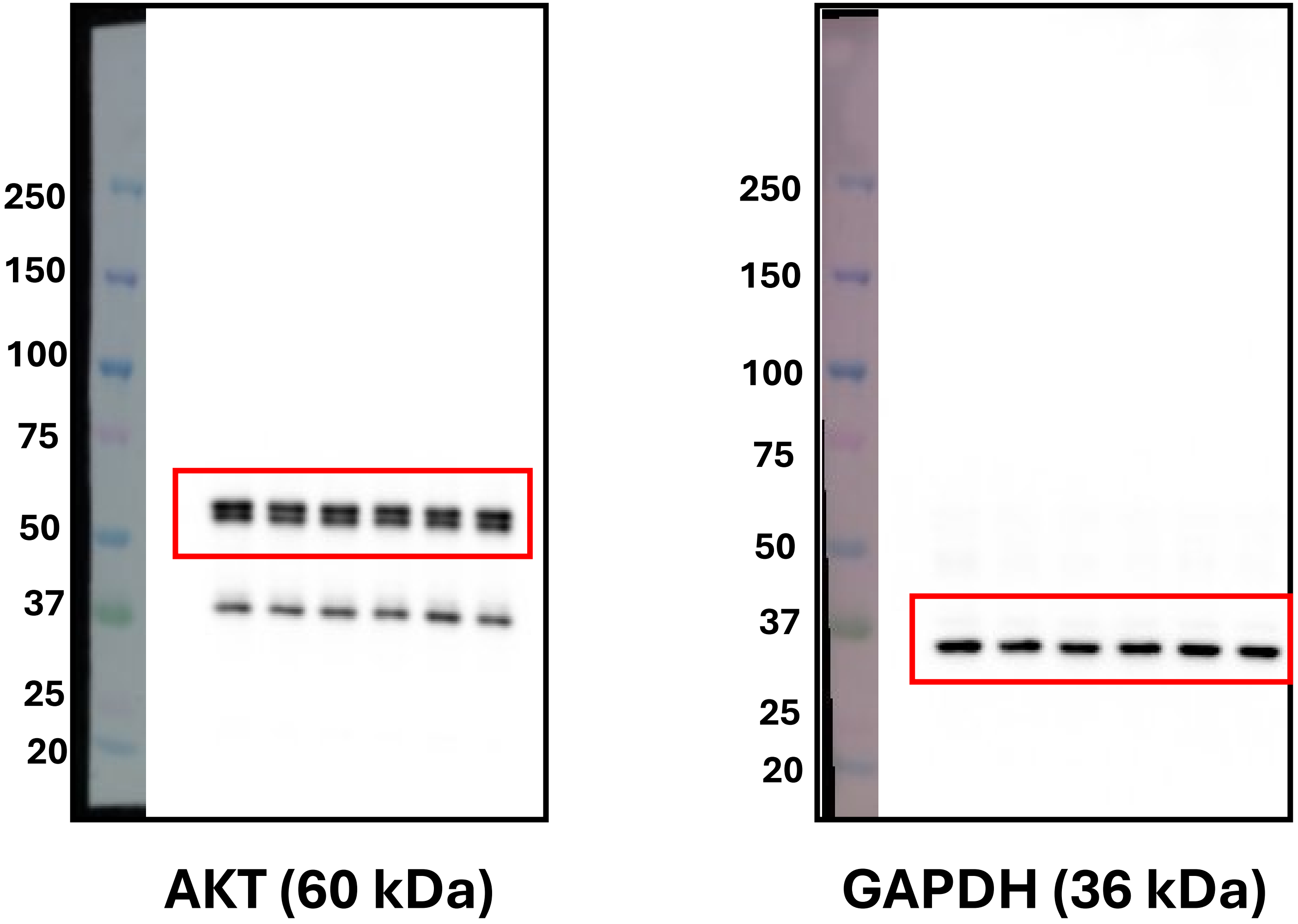
250
150
100
75
50
37
25
20
AKT (60 kDa)
250
150
100
75
50
37
25
20
GAPDH (36 kDa)

Fig S3 F

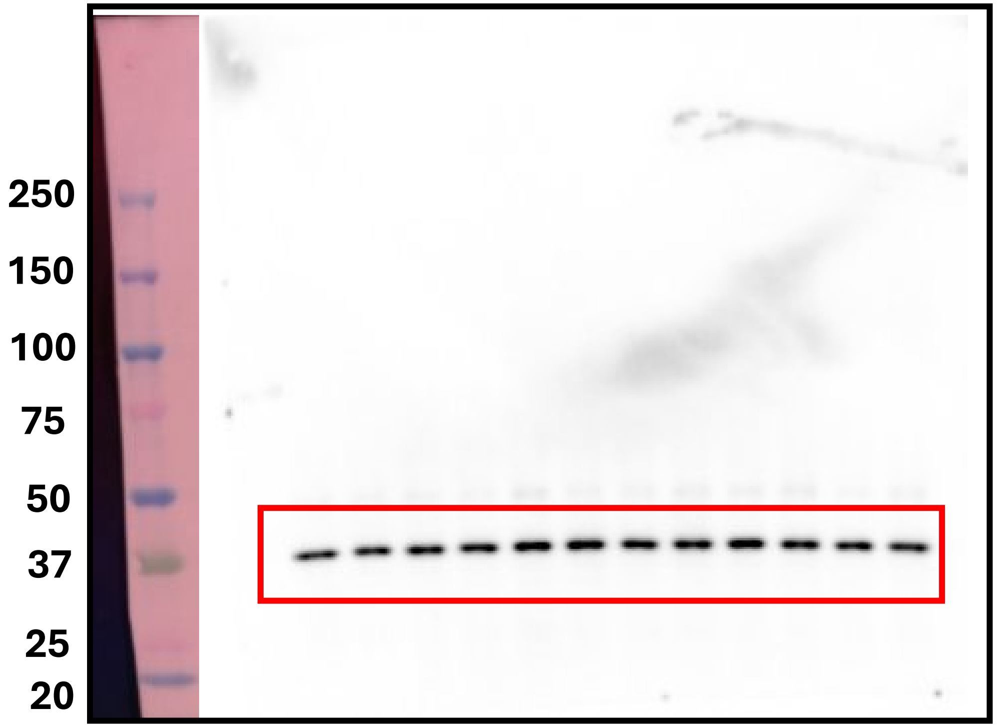

P38 MAPK (40 kDa)

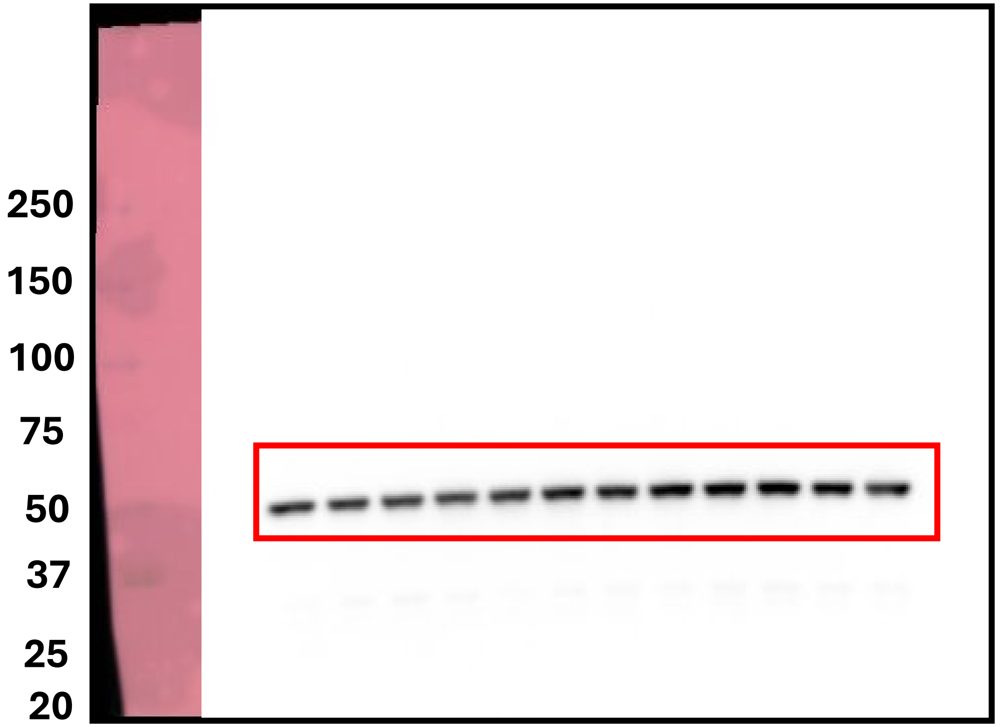

Tubulin (50 kDa)

Fig S3 G

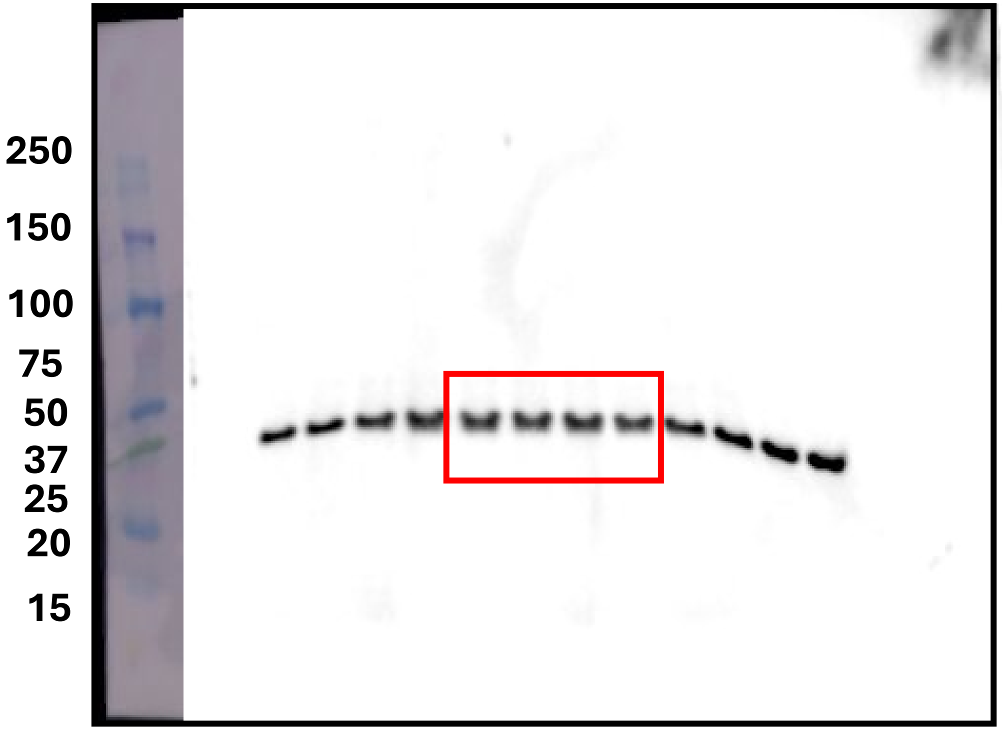

P38 MAPK (40 kDa)

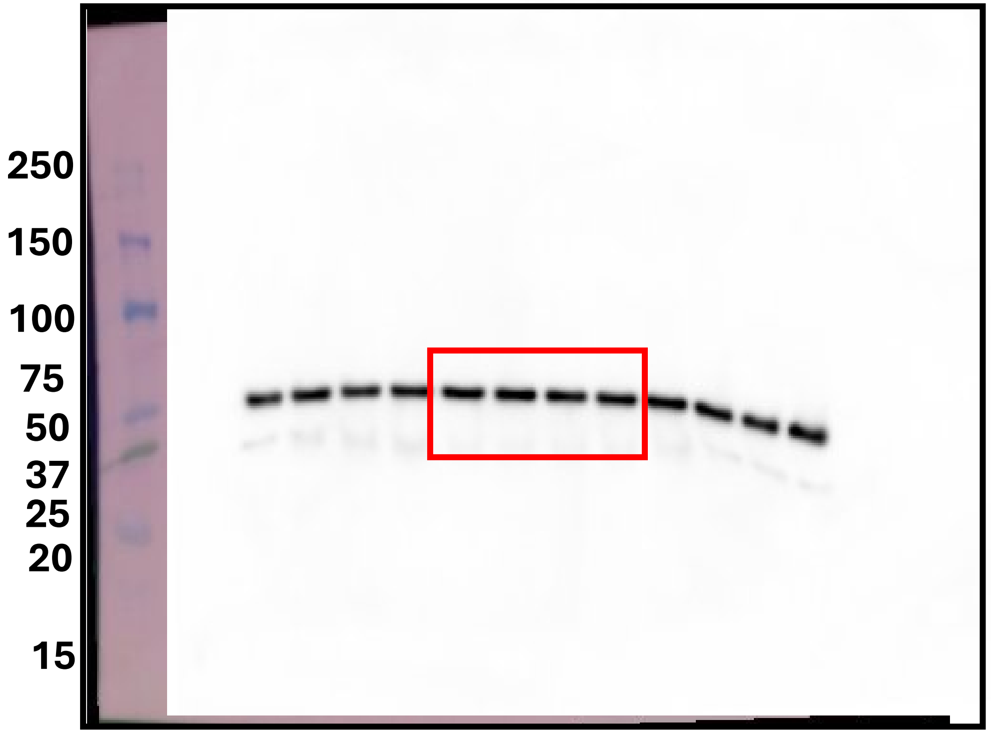

Tubulin (50 kDa)
